# Combination of hsa-miR-21-3p/ sTNF-RI/ IL12-p40 /CCL25 serves as a promising panel of diagnostic biomarkers for distinguishing malignant from benign nodules in papillary thyroid cancer

**DOI:** 10.1007/s12020-026-04612-9

**Published:** 2026-04-27

**Authors:** Abdulmelik Aytatli, Abdulkadir Sahin, Neslisah Barlak, Hasan Onur Caglar, Betul Gundogdu, Arzu Tatar, Omer Faruk KARATAS

**Affiliations:** 1 Molecular Biology and Genetics Department, Erzurum Technical University, Erzurum, Turkey; 2 Molecular Cancer Biology Laboratory, High Technology Application and Research Center, Erzurum Technical University, Erzurum, Turkey; 3 Department of Otorhinolaryngology Diseases, Faculty of Medicine, Ataturk University, Erzurum, Turkey; 4 Department of Medical Pathology, Faculty of Medicine, Ataturk University, Erzurum, Turkey

**Keywords:** Papillary thyroid cancer, microRNA, cytokine, miR-21-3p, proteome

## Abstract

**Purpose:**

Papillary thyroid cancer (PTC) is the most common thyroid malignancy and is often difficult to distinguish from benign nodules using current diagnostic tools, resulting in unnecessary surgical interventions. This study aimed to identify robust, reliable, and non-invasive circulating biomarkers capable of accurately discriminating benign from malignant PTC nodules by elucidating the relationship between thyroid tissue and circulating microRNA/cytokine profiles.

**Methods:**

Paired arterial plasma supplying the thyroid gland and venous plasma draining the gland were collected from patients with benign thyroid nodules and malignant PTC. In addition, preoperative and postoperative peripheral plasma samples were obtained. MicroRNA profiles were compared between arterial inflow and venous outflow to assess thyroid-derived contributions to circulating biomarkers. Differentially expressed microRNAs were validated in preoperative and postoperative peripheral plasma samples and formalin-fixed, paraffin-embedded (FFPE) thyroid tissue sections. Subsequently, cytokine profiles associated with target microRNAs were investigated using cytokine arrays, and selected target proteins were further validated by ELISA.

**Results:**

Arterial–venous comparisons revealed distinct microRNA and cytokine signatures associated with malignant PTC compared with benign nodules, reflecting tumor-specific release into the circulation. These findings were consistently validated in FFPE tissues and peripheral plasma samples. Importantly, the combined panel of hsa-miR-21-3p, sTNF-RI, IL-12p40, and CCL25 demonstrated high diagnostic performance in distinguishing malignant PTC from benign thyroid lesions.

**Discussion:**

This integrated tissue-to-circulation approach provides direct evidence that the thyroid microenvironment significantly contributes to the circulating biomarker landscape. The identified biomarker panel represents a promising non-invasive diagnostic tool with the potential to improve PTC diagnosis, reduce unnecessary surgical procedures, and support clinical decision-making.

**Supplementary Information:**

The online version contains supplementary material available at 10.1007/s12020-026-04612-9.

## Introduction

Thyroid cancer (TC) is the most common malignancy of the endocrine system, accounting for 2.2% of all cancer cases, with an estimate of 44,020 new cases in 2025 [1]. The global incidence of TC has increased significantly over the past few decades, primarily due to advances in imaging technology and enhanced detection of small, subclinical tumors [2]. Among differentiated TCs, papillary thyroid carcinoma (PTC) is the most common subtype, accounting for approximately 80–85% of all thyroid malignancies [3]. Although PTC typically exhibits slow growth, its steadily increasing incidence and potential for lymph node metastasis make it clinically significant [4]. Initial treatment options generally include surgical intervention and radioactive iodine therapy, with surgery remaining the cornerstone of management. The decision to proceed with surgery is often informed by diagnostic modalities such as fine-needle aspiration biopsy (FNAB), serum thyroid-stimulating hormone levels, and imaging techniques including high-resolution ultrasonography and computerized tomography scans [5]. However, despite frequent use of these conventional tools, preoperative cytology often fails to reliably differentiate between benign and malignant nodules, leading to unnecessary surgical resection (total thyroidectomy or lobectomy) even in cases that ultimately prove benign [6]. Therefore, there is an urgent need for development of more accurate diagnostic approaches that can discriminate benign from malignant thyroid nodules and predict tumor aggressiveness prior to surgery.

MicroRNAs (miRNAs) are short, single-stranded, non-coding RNAs with the potential to post-transcriptionally regulate gene expression. They bind to complementary sequences in the 3’ untranslated regions (3’ UTR) of their target mRNAs and then lead to mRNA degradation or suppression of protein translation [7]. Beyond their essential roles in physiological processes such as cell proliferation, differentiation, and apoptosis, miRNAs play critical roles in tumorigenesis [8]. Due to their remarkable stability in body fluids like serum, plasma, urine, saliva and cerebrospinal fluid, miRNAs have emerged as promising non-invasive biomarkers with potential applications in cancer diagnosis, estimation of prognosis, and therapeutic monitoring [9]. Their low molecular complexity, lack of post-transcriptional modifications, high conservation among species, and tissue-specific expression profiles further strengthen their suitability as diagnostic markers [10].

Several circulating miRNAs have been identified as potential biomarkers for TCs, particularly for detection of PTCs. Studies profiling serum miRNA expression have revealed numerous dysregulated miRNAs that can distinguish benign from malignant nodules [11]. However, despite extensive profiling efforts, inconsistencies across studies and the lack of standardized validation criteria have limited their clinical use. Beyond miRNAs, several protein-based biomarkers have been proposed to enhance the accuracy of PTC diagnosis. However, these protein biomarker candidates have not achieved universal clinical implementation due to variable sensitivity and specificity across different cohorts. Therefore, integrative approaches that combine molecular miRNA and proteomic markers may provide a more robust strategy for accurately diagnose malignant thyroid nodules preoperatively.

In this study, we aimed to identify novel plasma biomarkers capable of effectively distinguishing benign thyroid nodules from malignant lesions, thereby reducing unnecessary surgical interventions in patients with benign disease. It is well established that both miRNAs and cytokines reflect the molecular characteristics of their tissue of origin and are subsequently released into the circulation. To explore this tissue-to-circulation relationship, we collected paired arterial and venous blood (plasma) samples directly supplying and draining the thyroid gland during surgery, along with preoperative and postoperative peripheral plasma samples. By comparing the profiles of miRNAs and cytokines between arterial inflow and venous outflow in both benign and malignant cases, we delineated how the thyroid microenvironment contributes to the circulating biomarker landscape. Furthermore, candidate microRNAs and cytokines identified from arterial–venous plasma comparisons were validated in formalin-fixed, paraffin-embedded (FFPE) tissue sections and peripheral blood samples. Through this integrated approach, we proposed a panel of highly reproducible and diagnostically powerful plasma biomarkers for the accurate differentiation of benign and malignant thyroid diseases.

## Materials and methods

### Datasets and Identification of Differentially Expressed microRNAs

5 microarray (GSE113629, GSE151180, GSE103996, GSE73182, and GSE191117) and 4 RNA-Seq datasets (GSE63511, GSE124653, GSE159330, and GSE116196) deposited in the GEO database (https://www.ncbi.nlm.nih.gov/geo/) were utilized for in silico analysis of miRNA expression profiles across thyroid samples. Differentially expressed miRNAs (DE-miRs) between PTC tumor and normal samples from the five microarray datasets were identified using online GEO2R tool (http://www.ncbi.nlm.nih.gov/geo/geo2r/) [12]. RNA-Seq datasets were analyzed using the Subio Platform (Subio Inc., Kagoshima, Japan; https://www.subioplatform.com) [13]. In brief, raw RNA-Seq count data were imported to Subio Platform for normalization and analysis. In the normalization step, low-count filtering was applied by setting the lower limit between 5 and 10 read counts, and the noise range was defined between 0 and 20 to eliminate background fluctuations. Normalized expression values were then log2-transformed relative to the geometric mean of the control group. To exclude genes with minimal expression changes or technical noise, genes with log2 fold-change (log2FC) values between − 0.5 and 0.5 across all samples were filtered out. DE-miRs analysis between benign/normal thyroid samples and malignant thyroid specimens were conducted by GEO2R online web tool. All DE-miRs were exported as an xls file containing gene symbols, log fold change (logFC), and p-values. Genes with a p-value of < 0.05 and logFC ≥ 0.5 or logFC ≤ -0.5 were considered significantly differentially expressed. Genes with negative logFC values were classified as downregulated, while those with positive logFC values were classified as upregulated. After analyzing the DE-miRs from all collected datasets, Orange Canvas Software v3.30 was used to create heatmaps [14]. Common up- and downregulated miRNAs were identified and visualized in R using the “UpSet” function from the “ComplexHeatmap” Bioconductor package [15]. In silico analysis of miRNA expression in normal organs and circulation were carried out using the online miRGator database to prioritize the DE-miRs for their selection for further analysis [16].

### MicroRNA Target Prediction and Gene Ontology Analysis

miRDB (https://mirdb.org/), miRWalk (http://mirwalk.umm.uni-heidelberg.de/), TargetScan (https://www.targetscan.org/vert_80/), RNA22 (https://cm.jefferson.edu/rna22/), and miRMap (https://mirmap.ezlab.org/) databases were used to predict microRNA targets [17]. Venn diagrams were created using the Biotools online platform (https://www.biotools.fr/misc/venny). Gene Ontology enrichment analysis for common microRNA targets was performed using the Database for Annotation, Visualization, and Integrated Discovery (DAVID) (https://david.ncifcrf.gov/) [18], with a focus on the molecular function category. P values below 0.05 were considered statistically significant.

### Patients and Clinical Samples

Patients diagnosed with PTC or benign thyroid nodules who presented to the Department of Otorhinolaryngology at the Medical Faculty of Ataturk University were included into this study. The study was reviewed and approved by the Ataturk University Faculty of Medicine Institutional Review Board under decision number B.30.2.ATA.0.01.00/263. Written informed consents were obtained from all participants. All patients included in the study were confirmed not to have received radiotherapy or chemotherapy prior to surgery. In the benign and malignant cases, no evidence of Hashimoto’s thyroiditis, autoimmune thyroiditis, or any active inflammatory condition affecting thyroid function was identified. Patients diagnosed with Hashimoto’s thyroiditis or toxic diffuse goiter leading to thyroid dysfunction were not included in the primary analysis. These patients received appropriate medical management, and surgical intervention was undertaken only after the achievement of a euthyroid state. All samples analyzed in this study were obtained from patients with a definitive diagnosis and a clearly established surgical indication, confirmed during the preoperative, intraoperative, and postoperative periods. Cases presenting with any additional pathology that could potentially influence thyroid function were excluded from the study, and samples derived from such cases were not included in the analyses. The clinicopathological features of the patients are summarized in Table 1.

**Table 1:** Clinicopathological features of the patients

	Overall (70)	Benign (18)	Malign (52)	
**Age**	43.50 ± 14.19	37.83 ± 12.89	45.78 ± 14.24	
**Gender**,** n (%)**				
Female	56 (%80)	15 (%83.33)	41 (%78.84)	
Male	14 (%20)	3 (%16.66)	11 (%21.15)	
**T Classification**,** n (%)**				
T1 and T2	44 (%84.61)		44 (%84.61)	
T3 and T4	8 (%15.38)		8 (%15.38)	
**Lymphatic Metastasis (N stage)**,** n (%)**				
N0	46 (%88.46)		46 (%88.46)	
N1	6 (%11.53)		6 (%11.53)	
**Distant Metastasis**,** n (%)**				
M0	52 (%100)		52 (%100)	
M1	0 (%0)		0 (%0)	
**Histological Grade**				
Grade I	42 (%80.76)		42 (%80.76)	
Grade II	10 (%19.23)		10 (%19.23)	
Grade III	0 (%0)		0 (%0)	
Grade IV	0 (%0)		0 (%0)	

Patient samples included 30 plasma samples collected from thyroid artery of patients with malignant nodule, 30 plasma samples collected from thyroid vein of patients with malignant nodule (MVP), 9 plasma samples collected from thyroid artery of patients with benign nodule (BAP), 9 plasma samples collected from thyroid vein of patients with benign nodule (BVP), 15 peripheral plasma samples collected from patients with benign nodule (BPP), 30 peripheral plasma samples collected from patients with malignant nodule before surgery (Pre-OpPP), and 30 peripheral plasma samples collected from patients with malignant nodule one week after surgery (Post-OpPP).

2 ml of blood were collected from each collection site of a patient. All blood samples were transferred to EDTA tubes and centrifuged at 3,000 rpm for 10 min to prevent hemolysis and ensure that the plasma miRNA profile was not affected by erythrocyte-derived miRNAs. The resulting plasma was divided into 500 µL aliquots in eppendorf tubes and stored at − 80 °C until use. After centrifugation, hemolysis was assessed spectrophotometrically by measuring the optical density at 414 nm [20]. Samples with an optical density above 0.2 were considered as hemolyzed and excluded from the study.

Additionally, a total of 16 FFPE tissue samples (9 benign and 7 malignant) were used for validation purposes. 5-µm-thick sections were collected from each FFPE block and stored at 4 °C until RNA isolation.

### Total RNA Isolation and cDNA Synthesis

Total RNA, including small RNAs, was isolated from 300 µL of plasma using the EcoSpin Liquid Sample Total RNA Kit (EcoTech Biotechnology, Erzurum, Turkiye), following the manufacturer’s protocol. Concentration and purity of the RNA samples were measured using an Epoch 2 plate spectrophotometer. Samples with concentrations below 4 ng/µL were excluded. RNA samples were stored at − 80 °C until use. cDNA was synthesized using the TaqMan MicroRNA Reverse Transcription Kit (Applied Biosystems). Briefly, equal amounts of total RNA samples (20 ng/µL) were reverse-transcribed with sequence-specific stem-loop RT primers following the manufacturer’s protocol. Reactions were carried out in an Applied Biosystems™ MiniAmp™ Plus Thermal Cycler with the following parameters: 30 min at 16 °C, 30 min at 42 °C, and 5 min at 85 °C.

### Quantitative Real Time Polymerase Chain Reaction (qRT-PCR)

Expressions of hsa-miR-30a-3p and hsa-miR-21-3p was quantified using the microRNA-specific TaqMan MicroRNA Assays kit (Applied Biosystems) and TaqMan Universal Master Mix (Applied Biosystems) according to the manufacturer’s protocol in a Rotor-Gene Q system (Qiagen). All reactions were carried out in at least duplicates. miRNA expression levels were normalized to RNU6b [21]. Differential expressions of miRNAs were calculated using the delta delta Ct method as described previously [22].

### High-Throughput Plasma Cytokine Array

Human Cytokine Antibody Array (#AAH-CYT-7-2, RayBiotech, Norcross, GA) was employed to identify potential plasma biomarkers capable of clearly distinguishing benign versus malignant PTC cases. Antibody array analyses were conducted on plasma specimens collected from 30 cases, including six pairs of arterial and venous samples collected from patients with benign nodule, eight pairs of arterial and venous samples collected from patients with malignant nodule, eight pairs of peripheral blood plasma samples collected from patients with benign or malignant nodules, and eight pairs of preoperative and postoperative peripheral blood plasma samples collected from patients with malignant nodule. Proteins in Human Cytokine Antibody Array are listed in Supplementary Table S1. Cytokine profiling experiments were performed according to the manufacturer’s instructions. Briefly, plasma samples were pooled by group prior to analysis. Each pooled sample was diluted 1:1 with the kit’s blocking buffer and applied to nitrocellulose membranes, which were blocked, incubated with diluted plasma, washed, and then incubated with a cocktail of biotin-conjugated detection antibodies specific to the target proteins. Chemiluminescence was detected using a ChemiDoc MP imaging system (Bio-Rad, USA). Signal densitometry was measured using ImageJ software. Densitometric values on each membrane were corrected by subtracting blank values and normalized to positive controls. Fold-change values were calculated for each protein, and proteins meeting the significance threshold (*p* < 0.05) were selected for further analysis.

### Enzyme-Linked Immunosorbent Assay

Plasma concentrations of interleukin-12 p40 (IL-12p40), soluble tumor necrosis factor receptor I (sTNF-RI), and chemokine (C-C motif) ligand 25 (CCL25) were measured using commercially available enzyme-linked immunosorbent assay kits (YL Biont, Shanghai, China), according to the manufacturer’s instructions [23]. Prior to analysis, plasma samples were diluted 1:10 in phosphate-buffered saline. All reagents, standards, and samples were prepared as described in the kit protocols. Briefly, 100 µL of each diluted plasma sample, standard, and blank were added to the appropriate wells of a pre-coated 96-well microplate. The plate was incubated for 1 h at 37 °C, followed by five washing cycles with the kit’s wash buffer to remove unbound material. Subsequently, chromogen solutions A and B were added sequentially and incubated for 10 min at 37 °C to allow color development. The reactions were terminated by adding the stop solution, and the optical densities were immediately measured at 450 nm using an Epoch microplate reader (BioTek Instruments, USA).

Concentration of each analyte (IL-12p40, sTNF-RI, and CCL25) in plasma samples was calculated from the corresponding standard curve generated by four-parameter logistic regression. All measurements were performed in duplicates, and the mean values were used for subsequent statistical analyses.

### Statistical Analysis

Statistical analyses were performed using R software (version 4.3.2; R Foundation for Statistical Computing, Vienna, Austria), GraphPad Prism (version 10.0.3; GraphPad Software, San Diego, CA, USA) or Microsoft Excel. The distribution of continuous variables was evaluated using the Shapiro–Wilk test. Normally distributed data were compared using paired Student’s *t*-test, while non-normally distributed data were analyzed using the Wilcoxon signed-rank test for paired samples or Mann–Whitney U test for independent comparisons. Receiver operating characteristic (ROC) curves were generated to evaluate the power of hsa-miR-21-3p, IL12-p40, CCL25 and sTNF-RI individually and in combination to distinguish malign thyroid samples from benign cases by calculating the area under the ROC curve (AUC) values for each potential biomarker candidates. ROC analyses were conducted in R using the “pROC”[24] and **“**caret”[25] packages and visualized with “ggplot2” [26]. Optimal cut-off points were determined based on the maximum Youden’s *J* statistic. Also, positive predictive value (PPV), negative predictive value (NPV), 95% confidence interval (CI), sensitivity, and specificity values were calculated for each biomarker and for combinations of these biomarkers using the R caret package.

To assess whether the intra-individual changes observed in patients exhibited an increasing or decreasing trend in ELISA assays, standard deviations (SD) were initially calculated for each measurement. Thereafter, the coefficient of variation (CV%) was computed using the formula CV% = (SD / Mean) × 100. The analytical percentage Least Significant Change (%LSC) values were calculated using the equation %LSC = 2.77 × CV₍rms₎% (root mean square), as described previously [27]. LSC is estimated based on measurement error, which is derived from the root-mean-square coefficient of variation (CV₍rms₎%) of precision errors, and incorporates an adjustment factor corresponding to the selected level of statistical confidence. Typically, a two-tailed 95% confidence level is employed, for which a Z-score of 2.77 is applied in the formula %LSC = 2.77 × CV₍rms₎%. The calculated %LSC values were used as thresholds, and significant differences between sample pairs were evaluated based on the percentage differences between these values. Changes surpassing the LSC% threshold were categorized as significant increases, while those falling below the threshold were regarded as significant decreases. To statistically assess the directional significance of intra-individual changes, a one-sample binomial test was conducted using R software. Development of this methodological approach, as well as the review of relevant literature, was facilitated by the ChatGPT-5 Plus artificial intelligence model. To assess discriminative power of combination of biomarkers, a multiple logistic regression model was built. Predicted probabilities from this model were used to generate combined ROC curves and compute AUC values. Statistical significance was defined as *p* < 0.05.

## Results

### Analysis of Microarray and RNA-Seq Datasets Reveals Differentially Expressed miRNAs in TC

**Fig. 1:**
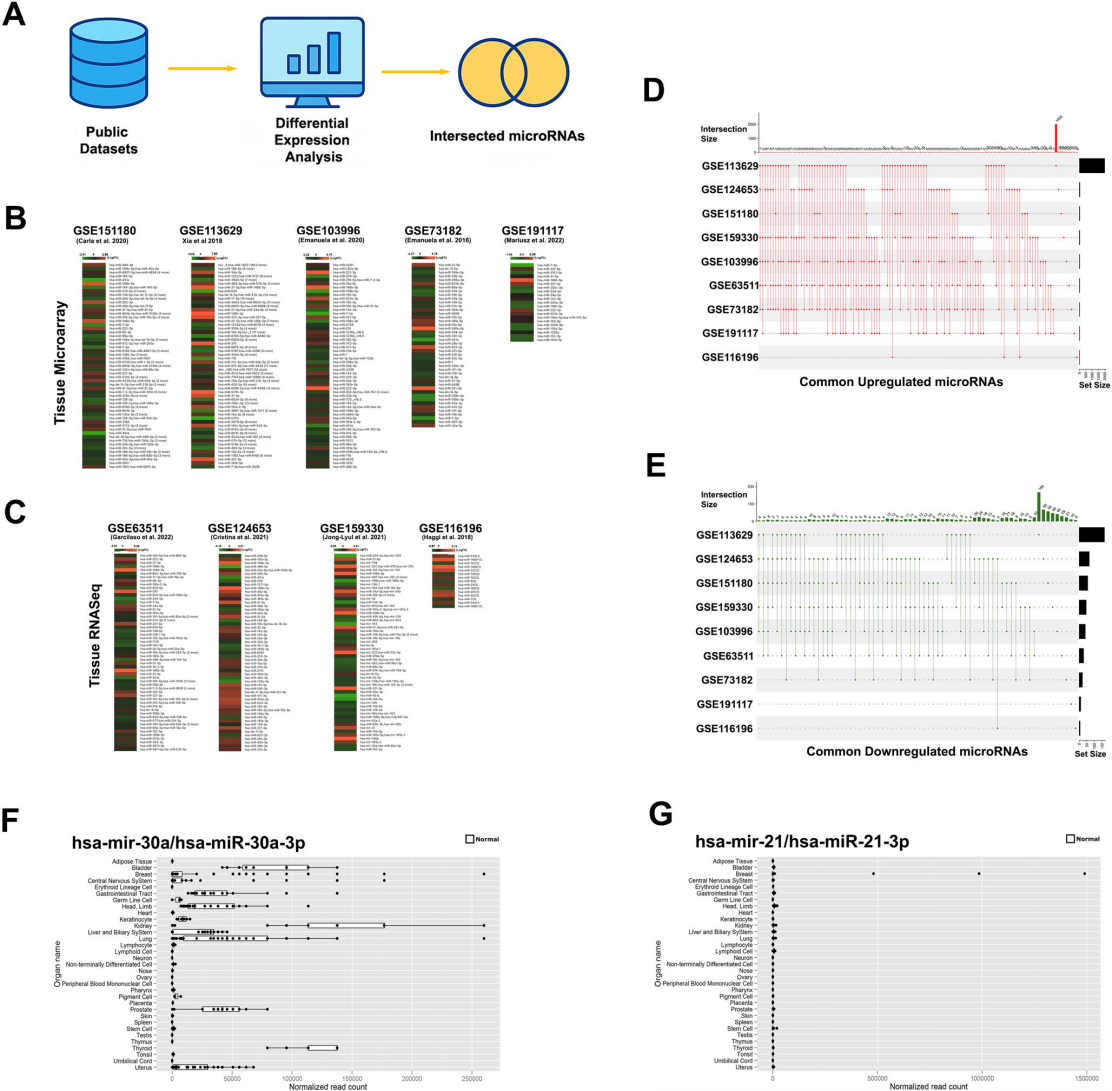
Identification of commonly differentially expressed microRNAs across public datasets and their organ-specific expression profiles in normal human tissues. **(A)** Schematic overview of the study workflow. Publicly available microarray and RNA-seq datasets were collected for differential expression analysis. Significantly altered microRNAs (log fold change > 0.5 for upregulation, log fold change < -0.5 for downregulation, *p* < 0.05) were intersected to identify common candidates across studies. **(B)** Heatmaps displaying differentially expressed microRNAs identified from tissue microarray datasets (GSE151180, GSE113629, GSE103996, GSE73182, and GSE191117). Each column represents an individual dataset, with colors indicating relative expression levels (red for upregulated; green for downregulated). **(C)** Heatmaps displaying differentially expressed microRNAs derived from tissue RNA-seq datasets (GSE63511, GSE124653, GSE159330, and GSE116196), visualized using the same color scale as in panel **(B)**. **(D)** UpSet plot illustrating the intersection of commonly upregulated microRNAs across all included datasets. Horizontal bars indicate the total number of upregulated microRNAs in each dataset, while vertical bars represent the size of intersections shared among datasets. **(E)** UpSet plot illustrating the overlap of commonly downregulated microRNAs across the analyzed datasets, displayed using the same conventions as in panel (**D**). **(F)** Tissue or organ expression distribution of hsa-miR-30a / hsa-miR-30a-3p across normal human tissues. Normalized read counts are shown for each tissue type. **(G)** Tissue-wide expression profile of hsa-miR-21 and hsa-miR-21-3p across normal human tissues, presented as normalized read counts

First, we analyzed the DE-miRs by comparing miRNA profile of PTC malignant cases with benign/normal thyroid nodules using GEO datasets, which included tissue microarrays and RNA-Seq data (Fig. 1A). A total of 2,690 miRNAs were found to be significantly differentially expressed in PTCs compared to normal or benign thyroid specimens, based on both tissue microarray and RNA-Seq datasets. Among these, 474 miRNAs were downregulated (shown in green), and 2216 miRNAs were upregulated (highlighted in red) (Fig. 1B, C). The list of upregulated and downregulated miRNAs in the datasets is provided in Supplementary Table S2.

Subsequently, commonly upregulated and downregulated DE-miRs were classified using the UpSet package in R software (Fig. 1D, E). Specifically, the expression levels of hsa-miR-146-5p/3p and hsa-miR-221-3p/5p were most significantly upregulated in the PTC specimens compared to benign/normal thyroid samples (Supplementary Table S2, Supplementary File 1). Conversely, the levels of hsa-miR-451a, hsa-miR-7-5p, and hsa-miR-204-5p were found to be significantly downregulated in PTC specimens (Supplementary Table S2, Supplementary File 1). These miRNAs exhibited consistent expression changes in at least six of the nine datasets analyzed (Supplementary File 1) and have been extensively reported in previous studies investigating PTCs [28]. However, different studies offered inconsistent results assigning oncogenic or tumor suppressor roles to the same microRNAs [11]. Besides, almost all of the top DE-miRs were found to be profoundly expressed in lymphocytes (Supplementary File 2), which may be misleading for biomarker selection due to potential enrichment of plasma and tissue miRNA profiles with the lymphocyte content. In addition, differential expression of those miRNAs, either downregulated or upregulated in thyroid specimens in different studies, can be due to the heterogenous nature of tumors in terms of lymphocyte infiltration [29]. Therefore, we intended to define a new criterion set for prioritization of commonly deregulated microRNAs for identification of biomarker candidates. Upregulated miRNAs (in tumor tissues) were selected based on their low expression levels in the normal thyroid tissues and circulation (lymphocytes and mononuclear blood cells) (Supplementary File 2), on the other hand, candidate downregulated miRNAs (in tumor tissues) were chosen based on their high expression in the thyroid tissue and relatively low expression in the circulation (lymphocytes and mononuclear blood cells) (Supplementary File 2).

To prioritize the deregulated miRNAs, their expression levels in normal organs and circulation were retrieved from the miRGator database. hsa-miR-30a-3p (miR-30a-3p) was identified as one of the most commonly downregulated miRNAs with high expression levels in the normal thyroid tissues along with relatively low expression in the circulation (Fig. 1F), while hsa-miR-21-3p (miR-21-3p) was among the most commonly upregulated miRNAs with low expression in the thyroid tissue and circulation together (Fig. 1G). Both miRNAs were found to be significantly differentially expressed in at least five of the nine analyzed datasets (Supplementary File 1). Therefore, to identify potential novel biomarkers, we focused our subsequent analyses on miR-21-3p and miR-30a-3p and selected them for further investigation.

### miR-21-3p Serves as a Potential Plasma Biomarker for Detection of Malignant Thyroid Lesions

**Fig. 2:**
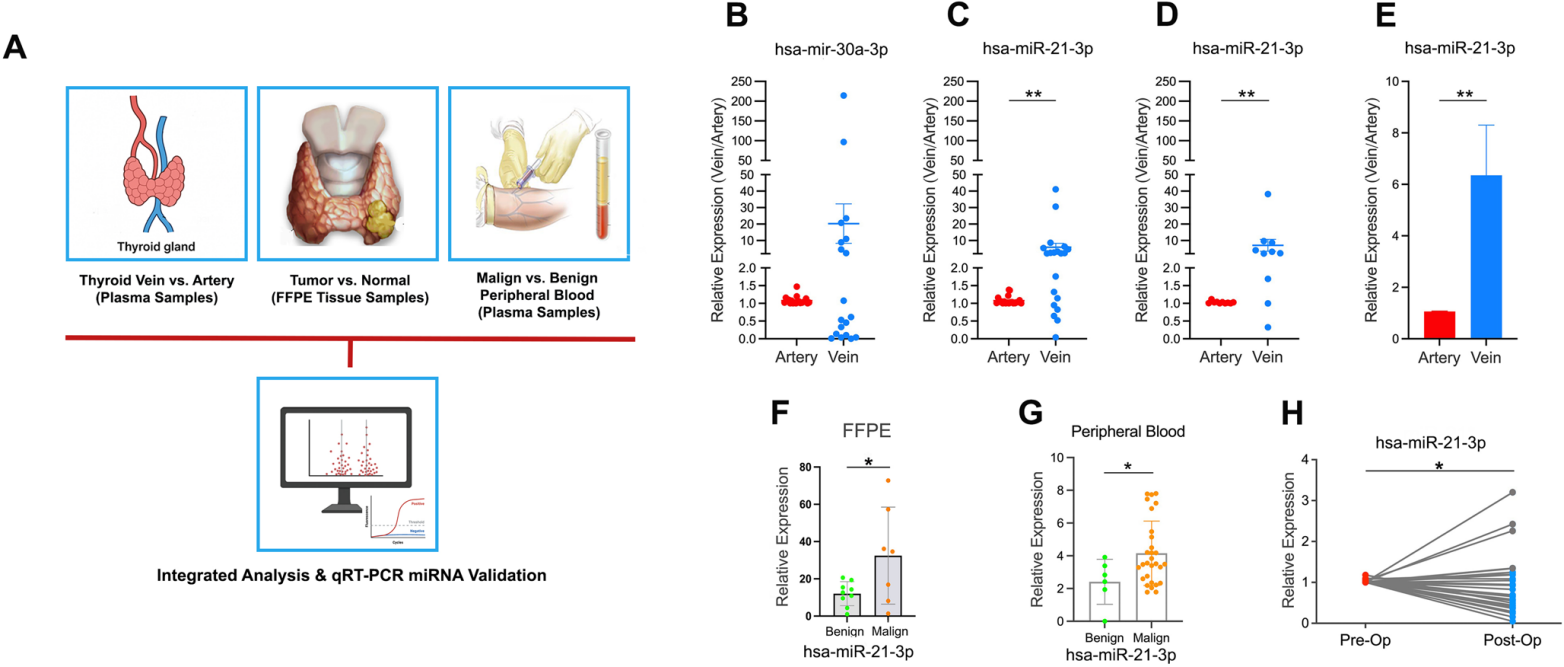
Validation of candidate microRNAs in tissue and plasma samples by integrated analysis and qRT-PCR. **(A)** Schematic presentation of the study design. microRNA expression was evaluated in thyroid vein versus artery plasma samples, tumor versus normal FFPE tissue samples, and malignant versus benign peripheral blood samples, followed by integrated analysis and qRT-PCR–based validation. **(B)** Relative expression levels of hsa-miR-30a-3p in plasma samples from the thyroid artery and vein. Each dot represents an individual sample; horizontal bars indicate the mean ± SD. **(C–E)** Relative expression of hsa-miR-21-3p in plasma samples from the thyroid artery and vein, analyzed in independent cohorts. Scatter plots **(C**,** D)** and the bar graph **(E)** demonstrate increased expression in venous samples compared to arterial samples. Each dot represents an individual sample; horizontal bars indicate the mean ± SD. **(F)** qRT-PCR analysis of hsa-miR-21-3p expression in FFPE thyroid tissue samples comparing benign and malignant lesions. **(G)** Relative expression of hsa-miR-21-3p in peripheral blood samples from patients with benign and malignant thyroid lesions. **(H)** Paired peripheral plasma samples were analyzed to assess hsa-miR-21-3p expression levels before (pre-OpPP) and after (post-OpPP) surgery, demonstrating postoperative changes in expression. Data are presented as mean ± SD. Statistical significance was determined using appropriate paired or unpaired tests; *p < 0.05 and **p < 0.01

To validate our in silico biomarker discovery pipeline, qRT-PCR was utilized to measure the expressions of selected miRNAs in clinical samples (Fig. 2A). As an initial attempt, expression levels of miR-21-3p and miR-30a-3p in 8 pairs of BAP and BVP samples were analyzed to exclude a potential differential expression between these two groups, which can be a sign of deregulation due to the contribution of thyroid organ itself to the bloodstream. Luckily, there were no significant differences in the expressions of miR-21-3p and miR-30a-3p between the plasma samples collected from the vein exiting the thyroid in comparison to the artery carrying blood to the organ. This result suggested that the levels of miR-21-3p and miR-30a-3p in the peripheral bloodstream were not affected by the thyroid organ with benign nodules (Supplementary Fig. 1).

Then, to be able to see whether the levels of miR-21-3p and miR-30a-3p in the plasma is affected by the malignant thyroid nodules, 20 plasma samples collected from the veins draining the blood from thyroid were compared to their pair plasma samples collected from arteries supplying blood to the thyroid in patients with malignant thyroid nodules. Initial evaluation for miR-30a-3p expression demonstrated a similar tendency (an increase with no significance) to what was observed in the comparison of BAP and BVP samples, which led to the conclusion for its insufficiency to be a diagnostic marker (Fig. 2B). Interestingly, same comparison for miR-21-3p in samples of patients with malignant thyroid tumors revealed a significant increase in miR-21-3p levels in MVP samples in comparison to MAP samples, suggesting miR-21-3p as a potential diagnostic marker since increase in its level in the plasma samples after blood visit the thyroid might be related to the presence of malignant lesion in the organ (Fig. 2C). To validate this increase, a second set of 10 plasma sample pairs collected from thyroid vein and artery of patients with malignant nodule was utilized, which confirmed the significant elevation of miR-21-3p levels in MVP samples relative to MAP samples (Fig. 2D). Overall analysis of all 30 samples pairs offered an approximate six-fold increase of miR-21-2p levels in venous plasma samples after they were drained from the thyroid organ (Fig. 2E). To see whether this change in miR-21-3p level in the plasma samples drained from the thyroid with malignant disease is directly attributable to biological differences in thyroid tissue, miR-21-3p expression levels were compared in nine benign and seven malignant thyroid FFPE samples. Its expression was found to be significantly higher in malignant samples compared to benign specimens, providing another level of proof for the diagnostic potential of miR-21-3p (Fig. 2F). Beyond these validation experiments, it is important to see whether the changes in the miR-21-3p level, associated with the malignant lesions present in the thyroid, persist in the peripheral bloodstream, since a powerful diagnostic tool should not require invasive biopsy applications. Therefore, next level of confirmation was carried out with plasma samples collected from peripheral bloods of patients with malignant or benign thyroid lesions. Interestingly, miR-21-3p levels were also found to be increased in peripheral plasma samples of patients with malignant thyroid nodules in comparison to those with benign nodules (Fig. 2G). This increase was found to be reversed in plasma samples after removal of malignant thyroid lesions (Fig. 2H). Altogether, these results provide several levels of proofs demonstrating the potential of miR-21-3p as a powerful diagnostic tool.

### Protein Array Reveals Additional Diagnostic Protein Biomarkers for More Accurate Detection of TC

**Fig. 3:**
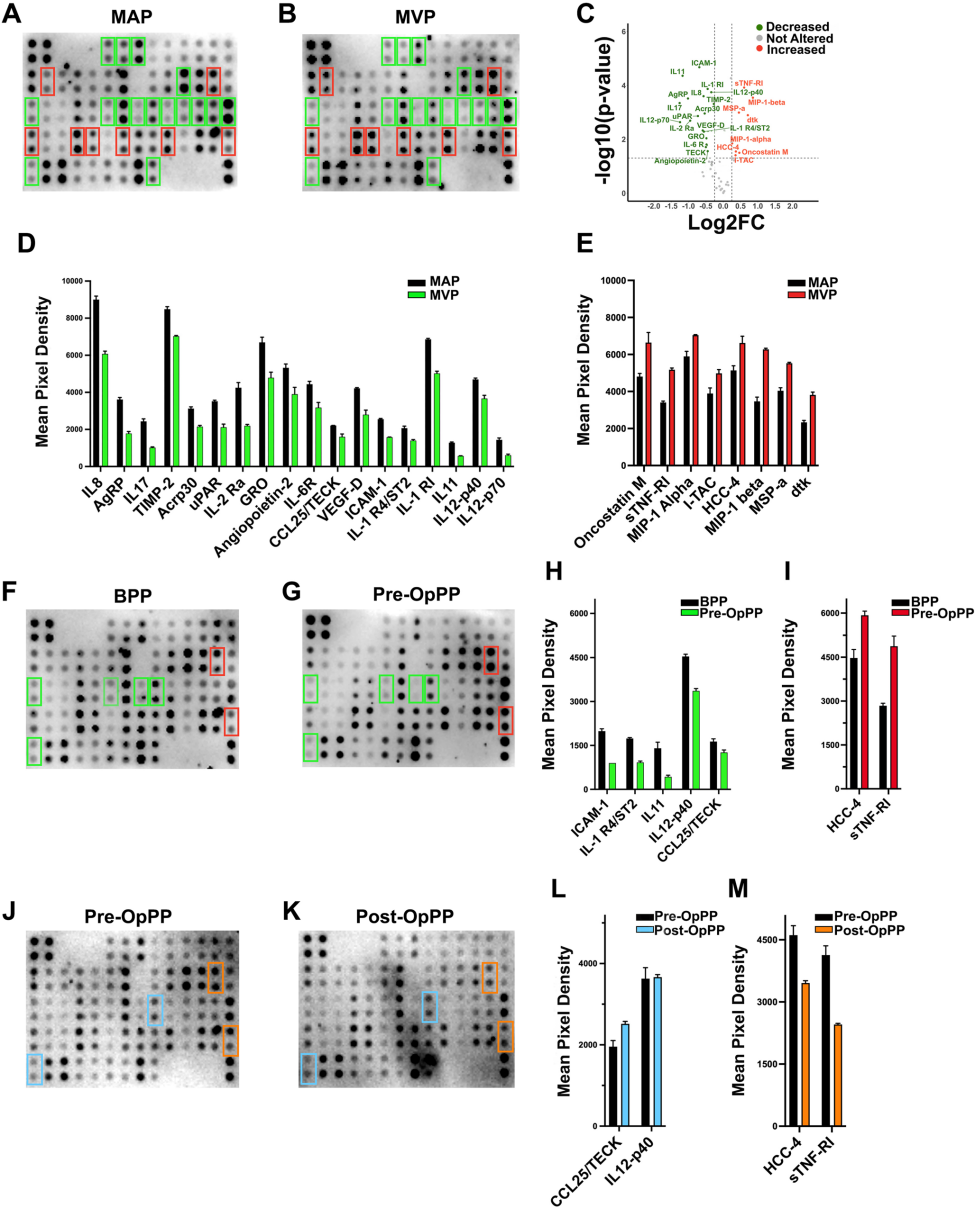
Protein array–based profiling of differentially expressed proteins across plasma sample groups. **(A, B)** Human Cytokine array C7 incubated with the eight pairs of MAP and MVP plasma samples. Each protein spot was represented in the array by two technical replicates. **(C)** The volcano plot represents the quantification of the cytokine profiles in MAP and MVP plasma samples and is used to summarize the results of the differential analysis. The x-axis shows the log₂ fold change, while the y-axis displays the − log₁₀ (p-value). Each point corresponds to protein analyzed. Statistically significant and increased proteins are indicated by a positive log₂ fold change and a high − log₁₀ (p-value), whereas decreased proteins have a negative log₂ fold change. Genes exceeding the defined threshold values (*p* < 0.05 and, -0.5 ≤|log₂ fold change| ≥ 0.5) are considered significantly differentially expressed. Array signals were quantitatively analyzed based on pixel densities, and the signal intensity for each protein was normalized relative to positive control spots, with each protein represented by two technical replicates. Protein expression levels are presented as normalized mean pixel densities ± standard deviation (SD) in bar graphs (panels **D-E**,** H-I**,** L-M).** Fold change values were calculated to assess relative differences between the groups and are detailed in the Supplementary Data file. Paired Student’s t-test was used to compare the groups. **(F-G)** Human Cytokine array C7 incubated with the eight pairs BPP and Pre-OpPP plasma samples. Each protein spot was represented in the array by two technical replicates. Panels **J-K** display representative array images obtained eight pairs of before and after surgery plasma samples, respectively

To further strengthen the power of miR-21-3p as a diagnostic biomarker differentiating the malign thyroid lesions from benign neoplasia, its estimated targets were investigated to see whether cytokines, which serve also as successful plasma markers for diagnosis of diseases, were enriched within the putative miR-21-3p targets. Interestingly, there were several target genes with functions to regulate cytokine expressions (Supplementary Fig. 2A-B). Therefore, to search for additional plasma biomarkers that might be utilized together with miR-21-3p to successfully differentiate malign lesions, we profiled the levels of several cytokines in the plasma samples collected from patients. Cytokine proteome profiling with Human Cytokine Array C7 (RayBiotech) comprised of 60 cytokines revealed that the levels of 26 cytokines were significantly different between the MAP and MVP groups, suggesting their potential to be used as diagnostic markers (*p* < 0.05) (Fig. 3A–C, Supplementary Table S3). Among the deregulated proteins, 18 showed decreased expression, including members of the interleukin and chemokine families such as IL-12p40, IL-12p70, IL-11, IL-6R, IL-2Ra, IL-17, and CCL25, as well as VEGF-D, a previously identified thyroid cancer biomarker (Fig. 3D), whereas, eight proteins were upregulated across the groups (Fig. 3E). To further filter these 26 cytokines, the same cytokine array was used to compare BAP and BVP thyroid plasma samples to find out proteins whose levels did not change significantly between the plasma samples drained from and entering the thyroid tissue of patients with benign lesions, since these proteins will have the capacity to reflect the malignity but not the benign conditions. Post-array analysis of the BAP-BVP plasma samples demonstrated that of the 26 proteins whose expression was significantly altered in the MAP-MVP comparison, 15 cytokines levels either did not change significantly or changed in the opposite direction in the BAP-BVP cohorts (Supplementary Fig. 3A-C), implying their specificity to malign lesions in the thyroid. Further analysis was carried out using peripheral plasma samples collected from patients with malign and benign thyroid lesions to see whether the contribution of malign thyroid lesions to the bloodstream can be reflected to and detected in the systemic circulation. Interestingly, seven of the 15 proteins exhibited significant differences in their levels in the bloodstream of PTC patients in comparison to plasma of patients with benign nodule (Fig. 3F, G, Supplementary Table S3). More specifically, ICAM-1, IL-1R4/ST2, IL-11, IL-12 p40, and CCL25/TECK levels decreased (Fig. 3H), HCC-4 and sTNF-R1 levels increased (Fig. 3I) in the peripheral plasma of patients with malign thyroid nodules, suggesting the fact that the changes in the level of these cytokines between thyroid arterial and venous plasma samples somehow are reflected in the systemic circulation. Then, levels of these seven proteins were investigated utilizing the same protein array to see whether their levels were reversed back to the normal in the peripheral plasma samples after removal of the malign thyroid lesions (Fig. 3J, K, Supplementary Table S3). As another proof of concept, 4 of them had reestablished levels in the plasma samples after thyroidectomy, implying their profound potential as powerful diagnostic biomarkers (Fig. 3L, M).

### ELISA Confirms Differential Levels of IL12-p40, CCL25/TECK and sTNF-RI in Plasma of PTC Patients

**Fig. 4:**
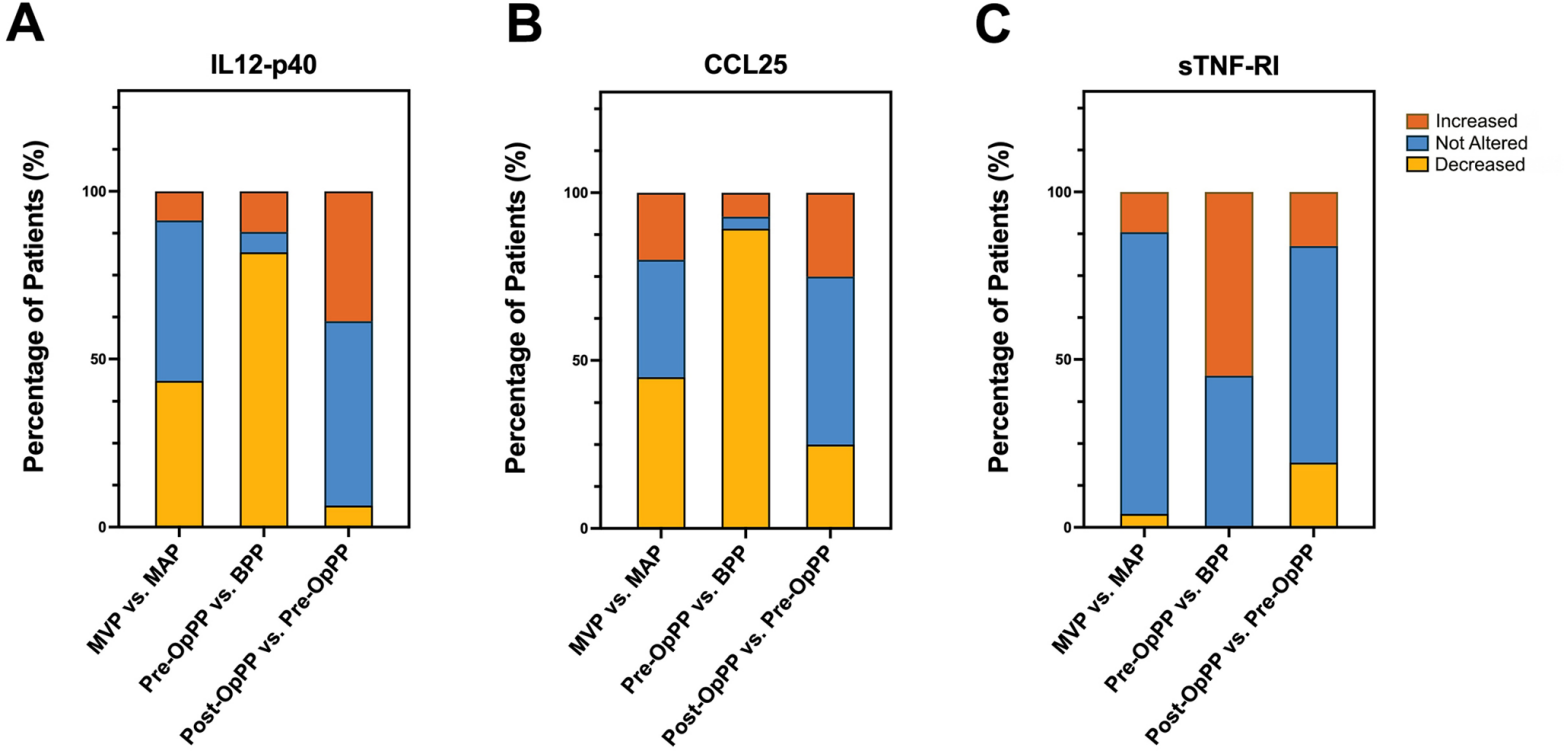
IL12-p40, CCL25, and sTNF-RI serve as potential plasma biomarkers for accurately distinguishing benign from malignant thyroid nodules.**(A)** Serum IL-12p40 levels were measured using ELISA in the MVP vs. MAP, BPP vs. Pre-OpPP, and Pre-OpPP vs. Post-OpPP groups. Analyses were performed within individuals (paired). Patient samples were compared, and the lowest threshold for significant change was determined based on the %LSC value. Using this threshold, patients were classified as having statistically significant increases, decreases, or no change in IL-12p40 levels, and the results were expressed as patient percentages (%). A One-Sample Binomial Test was applied to assess the significance of these percentages. The graph illustrates the distribution of the direction of change in IL-12p40 levels within the MVP vs. MAP, BPP vs. Pre-OpPP, and Pre-OpPP vs. Post-OpPP groups. Similarly, analyses were performed for CCL25 in panel **B** and for sTNF-RI in panel **C**

Further validation using ELISA in larger sample cohorts demonstrated that a significant portion of the patients had decreased IL12-p40 or CCL25 levels in the thyroid vein relative to the artery in patients with malign lesion and in the peripheral plasma samples of those with malign nodules, which is in line with the array results. Besides, the levels of these potential biomarkers did not show statistically significant changes in the benign group (Fig. 4A, B, Supplementary Fig. 4). Although sTNF-RI levels in thyroid vein vs. artery comparison in patients with malign lesion seems to be moderately increased, its levels were higher in more than half of the peripheral plasma samples of those with malign tumors in comparison to those with benign lesions (Fig. 4C). Interestingly, there was no change between the sTNF-RI levels in the plasma samples drained from and entering the thyroid tissue of patients with benign lesions (Supplementary Fig. 4). Besides, after removal of the malign thyroid lesions, sTNF-RI levels were found to be decreased in the some of the peripheral plasma samples of the patients (Fig. 4C), confirming its diagnostic potential.

### miR-21-3p Together with IL12-p40, CCL25/TECK and sTNF-RI Have Profound Power to Differentiate Malign from Benign Lesions

**Fig. 5:**
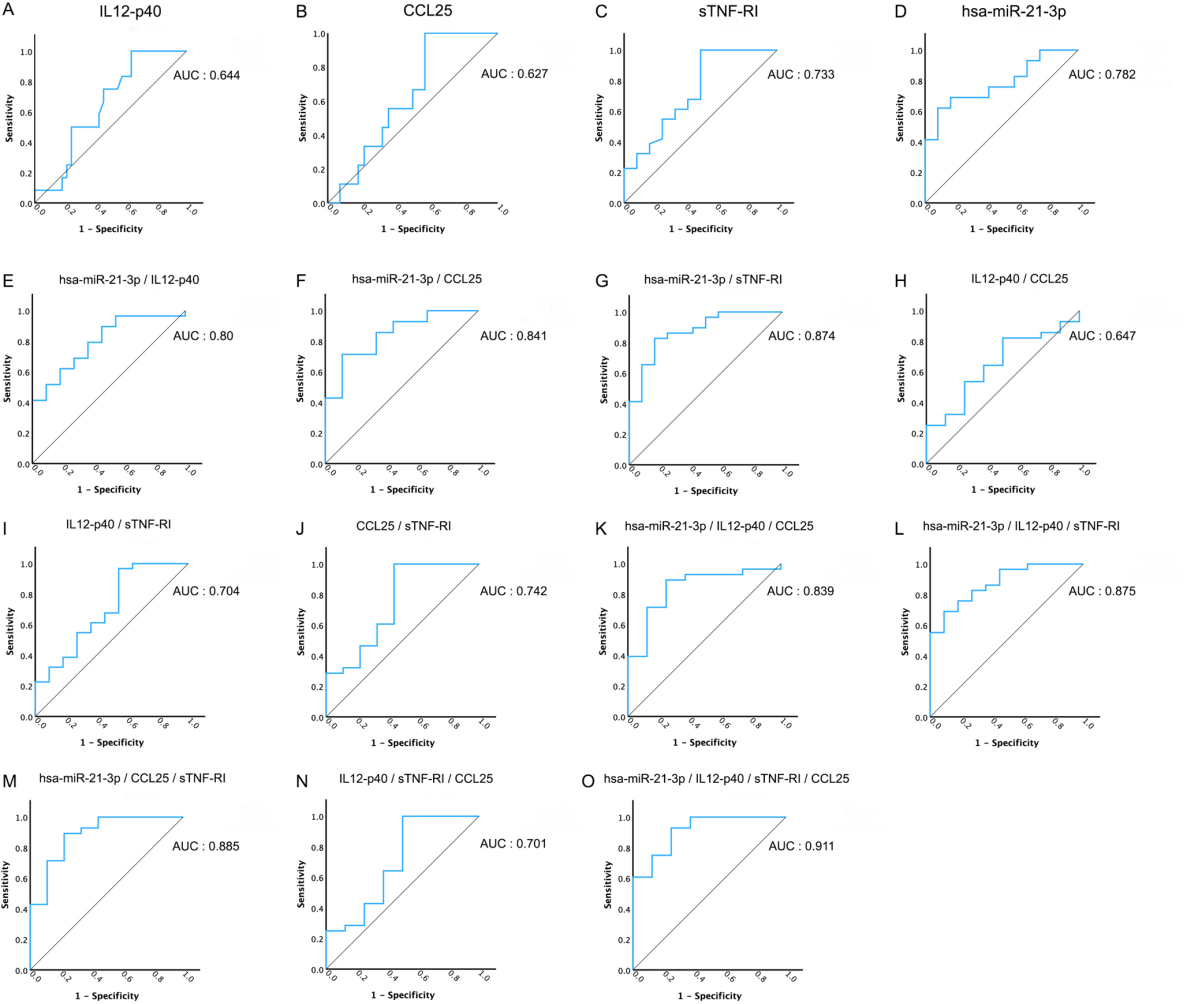
has-miR-21-3p, IL12-p40, CCL25, and sTNFRI combination serve as potential biomarkers for accurately distinguishing benign from malignant thyroid nodules. **(A)** The power of IL12-p40 to discriminate benign and malignant samples on its own. **(B)** The power of CCL25 to discriminate benign and malignant samples on its own. **(C)** The Power of sTNF-RI to discriminate benign and malignant samples on its own. **(D)** The power of has-miR-21-3p to discriminate benign and malignant samples on its own. **(E)** The power of has-miR-21-3p and IL12-p40 together to discriminate benign and malignant samples. **(F)** The power of has-miR-21-3p and CCL25 to discriminate benign and malignant samples. **(G)** The power of has-miR-21-3p and sTNF-RI together to discriminate benign and malignant samples. **(H)** The power of IL12-p40 and CCL25 together to discriminate benign and malignant samples. **(I)** The power of IL12-p40 and sTNF-RI together to discriminate benign and malignant samples. **(J)** The power of CCL25 and sTNF-RI together to discriminate benign and malignant samples. **(K)** The ability of has-miR-21-3p, IL12-p40, and CCL25 to distinguish between benign and malignant samples on combination. **(L)** The ability of hsa-miR-21-3p, IL12-p40, sTNF-RI to distinguish between benign and malignant samples on combination. **(M)** The ability of has-miR-21-3p, CCL25, and sTNF-RI to distinguish between benign and malignant samples on combination. **(N)** The ability of IL12-p40, sTNF-RI, and CCL25 to distinguish between benign and malignant samples on combination. **(O)** The ability of has-miR-21-3p, IL12-p40, sTNF-RI, and CCL25 to distinguish between benign and malignant samples on combination

**Fig. 6:**
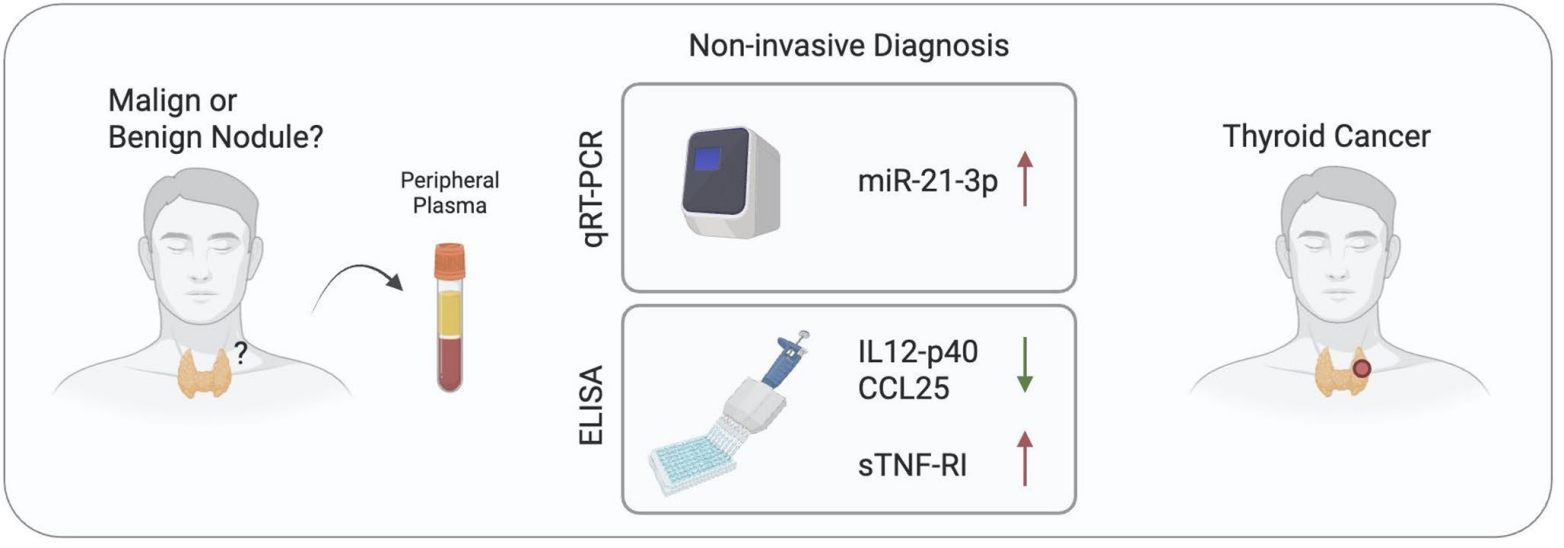
Schematic overview of the diagnostic potential of the tested biomarker candidates

ROC analyses were conducted using biomarker candidate levels measured by qRT-PCR and ELISA in peripheral samples obtained from benign cases and in peripheral samples collected during the pre-surgical period from malignant cases (Supplementary File 4). The ability of miR-21-3p, IL12-p40, CCL25, and sTNF-RI to differentiate malignant from benign lesions was evaluated using ROC analysis, which demonstrated their moderate discriminatory power individually (AUC values ranging from 0.627 to 0.782) (Fig. 5A-D). Their 2- and 3-piece combinations resulted in a considerable increase in their power to more accurately distinguish benign thyroid nodules from malignant ones (Fig. 5E-N). More promisingly, miR-21-3p, IL12-p40, CCL25 and sTNF-RI together, with an AUC value of 0.911, displayed the highest potential to assign the malignant nature of a lesion via only utilizing the peripheral plasma samples (Fig. 5O). In addition to AUC values, sensitivity, specificity, positive predictive, negative predictive, and accuracy values are presented in Supplementary File 3. Consequently, we offer a novel plasma biomarker set capable of effectively distinguishing benign thyroid nodules from malignant ones, thereby potentially reducing unnecessary surgical interventions in patients with benign disease. Our integrative approach that combine molecular miRNA and proteomic marker analysis may provide a more robust strategy for accurately differentiation of benign and malignant thyroid diseases (Fig. 6).

## Discussion

Currently, the most commonly used methods in clinical practice for diagnosing TC are ultrasonography, FNAB, histopathological examination, and molecular testing [30]. Although ultrasonography is a non-invasive method, it is insufficient for diagnosis on its own due to the significant overlap in ultrasonographic features between benign and malignant nodules, as well as its limited specificity, which complicates the diagnostic process [31]. Although FNAB is considered the gold standard for evaluating thyroid nodules, it can produce questionable results, with false positive rates as high as 46.4% and false negative rates up to 39.72%, often necessitating repeated procedures [30,32]. In addition, radioactive iodine therapy, chemotherapy, immunotherapy, surgical approaches (including total thyroidectomy), and molecularly targeted therapies are frequently used in the treatment of TC [33]. Cases in which malignancy is detected during a histopathological examination of thyroid tissue which is removed for benign causes, such as multinodular goiter or Basedow’s disease, are classified as “incidental PTC.” In contrast, cases in which a malignant focus is discovered later despite no tumor being detected in clinical or imaging evaluations prior to surgery are classified as “occult PTC.” These two situations show the need for diagnostic approaches that can more accurately and reliably differentiate malignant lesions from benign thyroid diseases before surgical intervention [34].

Galectin-3 (Gal-3), a β-galactoside-binding lectin involved in cell adhesion, proliferation, and apoptosis, is consistently overexpressed in malignant thyroid lesions compared to benign nodules. It is considered one of the most reliable immunohistochemical markers for distinguishing PTC from benign thyroid disease [35]. Although significant differences were observed in FNAB samples, serum Galectin-3 levels did not differ significantly between patients with PTC and healthy individuals [36]. Despite their diagnostic value, tissue-based immunohistochemical markers are limited in their clinical use in routine, inexpensive, rapid, and non-invasive diagnostic processes. Similarly, cytokeratin-19 (CK19), an intermediate filament protein abundantly expressed in PTC cells, has been used as a complementary marker in diagnostic pathology to support cytological and histological findings [37]. Interestingly, the expression pattern of CK-19 is highly controversial. While some studies assert that it is expressed exclusively in malignant lesions, its presence has also been demonstrated in benign lesions [38]. In addition to CK19 and Galectin-3, angiopoietin-1 (Ang-1) and tissue inhibitor of metalloproteinases-1 (TIMP-1) are upregulated in PTC and have attracted attention as serum protein biomarkers for diagnosis of PTC [39]. Given the prolonged turnaround time, invasiveness, and high cost of immunohistochemical and tissue-based assays, increasing attention has shifted toward rapid, reliable, and cost-effective circulating molecular biomarkers.

In recent years, molecular biomarkers obtained from non-invasive samples have attracted significant attention. MiRNAs and cytokines/chemokines, which are particularly abundant in circulating body fluids such as seminal fluid, plasma, blood, and urine, have emerged as promising molecules for diagnosis of early-stage cancers and estimate the prognosis [40].Changes in tissue miRNA levels have been linked to various types of cancers, where they can function as oncogenes or tumor suppressors. Numerous studies demonstrate that miRNAs play a crucial role in cancer initiation, progression, and metastasis [41]. Beyond their independent roles in tumor biology, microRNAs are increasingly recognized as key regulators of inflammatory signaling pathways within the tumor microenvironment [42]. Cytokines and chemokines play crucial roles in shaping the tumor microenvironment and regulating the inflammatory response [43]. Interestingly, It has been demonstrated that microRNAs can directly regulate cytokine expression at the post-transcriptional level, and that inflammatory cytokines can, in turn, modify microRNA expression profiles [44]. Indeed, a study by Patel et al. demonstrated that the pro-inflammatory cytokine IL-6, released by immune cells, enhances the invasive capacity of colorectal cancer cells. Specifically, the study reported that in the presence of IL-6, cancer cells secrete circulating microRNAs miR-21 and miR-29b, which in turn stimulate IL-6 production and activation in immune cells. Additionally, activated immune cells release miR-21 into the tumor microenvironment. Collectively, these findings suggest that the bidirectional interaction between tumor cells and immune cells is mediated through the microRNA–cytokine axis, providing significant mechanistic regulation within the tumor microenvironment [45]. Another study demonstrates that the IL-6–miR-21–PDCD4 axis plays a significant regulatory role in the development of prostate cancer. This molecular interaction has been demonstrated to have the potential to serve as an important biomarker for gene therapy approaches and disease diagnosis [46]. Additionally, cytokine production activates immune cells, which then release growth factors and cytokines into the tumor microenvironment, thereby initiating inflammation [46]. All in all, research demonstrates that many miRNAs directly or indirectly regulate cytokine expression, while cytokines can also affect microRNA biogenesis and stability. This bidirectional regulatory relationship has significant biological implications for inflammation-related diseases, autoimmune disorders, and various types of cancers, making the miRNA–cytokine axis a valuable source of diagnostic biomarkers. In our study, one of the targets of hsa-miR-21-3p is estimated to be IL12-p40. Gene ontology enrichment analyses also revealed that the target genes of hsa-miR-21-3p are associated with the regulation of interferon, cytokine activity, antiviral defense responses, cellular responses to xenobiotic stimuli, and inflammatory processes.

In colorectal cancer, the cytokine network includes important biomarkers that are valuable for both pathogenesis and prognosis, providing a non-invasive and cost-effective diagnostic option. Notably, pro-tumorigenic cytokines such as IL-4, IL-6, IL-8, IL-11, IL-17 A, IL-22, IL-23, IL-33, TNF, TGF-β, and VEGF play significant roles at various stages of colorectal cancer [47]. IL-12, known as natural killer cell-stimulating factor, is a heterodimeric cytokine with a molecular weight of 70–75 kDa, composed of two subunits: 35 kDa (p35) and 40 kDa (p40) [48]. These observations are primarily derived from colorectal cancer models, but similar inflammatory and cytokine-driven mechanisms have also been associated with thyroid carcinogenesis [49].

IL12-p40 is also one of the cytokines that play an important regulatory role in cancer pathogenesis. Previous studies have shown that IL-12 p40 may serve as a biomarker for diagnosing rheumatoid arthritis and osteoporosis [50]. TNF is a key pro-inflammatory cytokine involved in numerous cellular processes, including cell proliferation, differentiation, apoptosis, and the induction of other cytokines. The cellular response triggered by tumor necrosis factors is mediated by Tumor Necrosis Factor Receptor 1 (TNF-R1) and Tumor Necrosis Factor Receptor 2 (TNF-R2). Their soluble forms, sTNF-R1 and sTNF-R2, are regarded as highly promising serum biomarkers in plasma or blood samples due to their extended half-lives and greater stability compared to TNF-α. Serum levels of sTNF-R1 have been shown to be elevated in human colorectal adenomas and may contribute to colorectal cancer development, suggesting its potential as a biomarker [51]. Chemokines, a type of cytokine, play a crucial role in cell migration and the regulation of the tumor microenvironment. They have also been associated with tumor progression in certain malignancies [52]. CCL25 (also known as TECK) is highly expressed in the thymus and plays a crucial role in T cell development [53]. In addition, CCR9 expression is significantly elevated in non-small cell lung cancers (NSCLC). Its natural ligand, CCL25, has also been reported to be highly expressed in multiple studies of NSCLC. Both CCR9 and CCL25 are associated with poor prognosis in lung cancer, making them notable unfavorable prognostic markers [54]. Current data indicate that in many cancers and diseases such as osteoporosis and rheumatoid arthritis, cytokines and chemokines may serve as potential biomarkers that can be evaluated collectively for disease diagnosis.

This study demonstrates that the combined evaluation of hsa-miR-21-3p expression and the plasma levels of sTNF-RI, CCL25, and IL-12p40 in peripheral plasma samples may constitute a highly discriminatory biomarker panel for differentiating benign from malignant lesions in patients with suspected PTCs. This minimally invasive, rapid, and cost-effective approach requires no specialized personnel and can serve as a supportive tool in the clinical diagnostic process. A significant limitation of this study is the small sample size of the cohort. Larger, multicenter studies are necessary to validate these findings. Additionally, longitudinal analyses involving samples collected at multiple time points could more deeply elucidate the relationship between these biomarkers, disease progression, and treatment response, thereby advancing this research further. In this context, the proposed biomarker panel holds promise for improving the diagnosis of TCs in clinical practice in the future.

## Supplementary Information

Below is the link to the electronic supplementary material.

Supplementary Material 1

Supplementary Material 2

Supplementary Material 3

Supplementary Material 4

Supplementary Material 5

Supplementary Material 6

Supplementary Material 7

Supplementary Material 8

## Data Availability

The data that support the findings of this study are available from the corresponding author upon reasonable request.
