# Supplementary material for: Combination of hsa-miR-21-3p/ sTNF-RI/ IL12-p40 /CCL25 serves as a promising panel of diagnostic biomarkers for distinguishing malignant from benign nodules in papillary thyroid cancer
Source: Endocrine. 2026 Apr 27;91(1):146. doi: 10.1007/s12020-026-04612-9 (PMC13121238; doi:10.1007/s12020-026-04612-9)
Supplement: Supplementary file 1 — Supplementary Material 1 [file 12020_2026_4612_MOESM1_ESM.pdf]

Combination of hsa-miR-21-3p/ sTNF-RI/ IL12-p40 /CCL25 serves as a promising panel of diagnostic biomarkers for distinguishing malignant from benign nodules in papillary thyroid cancer.

Abdulmelik Aytatli[1,2], Abdulkadir Sahin[3], Neslisah Barlak[1,2], Betul Gundogdu[4], Arzu Tatar[3], Omer Faruk KARATAS[1,2,*]

Supplementary Figures

Supplementary Figure 1. Validation of candidate microRNAs in benign plasma samples by qRT-PCR

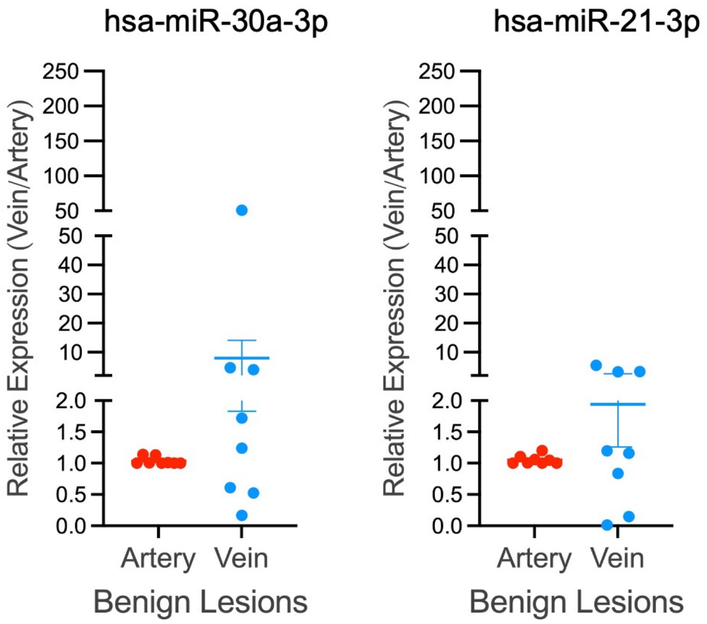

Relative expression of hsa-miR-30a-3p in benign plasma samples from thyroid artery and vein. Each dot represents an individual sample; horizontal bars indicate the mean ± SD. Relative expression of has-miR-21-3p in benign plasma samples from thyroid artery and vein. Each dot represents an individual sample; horizontal bars indicate the mean ± SD.

Supplementary Figure 2. hsa-mir-21-3p is strongly associated with cytokines, cytokine regulatory genes, interferon-alpha production, and genes involved in cellular responses to external stimuli such as viruses and bacteria

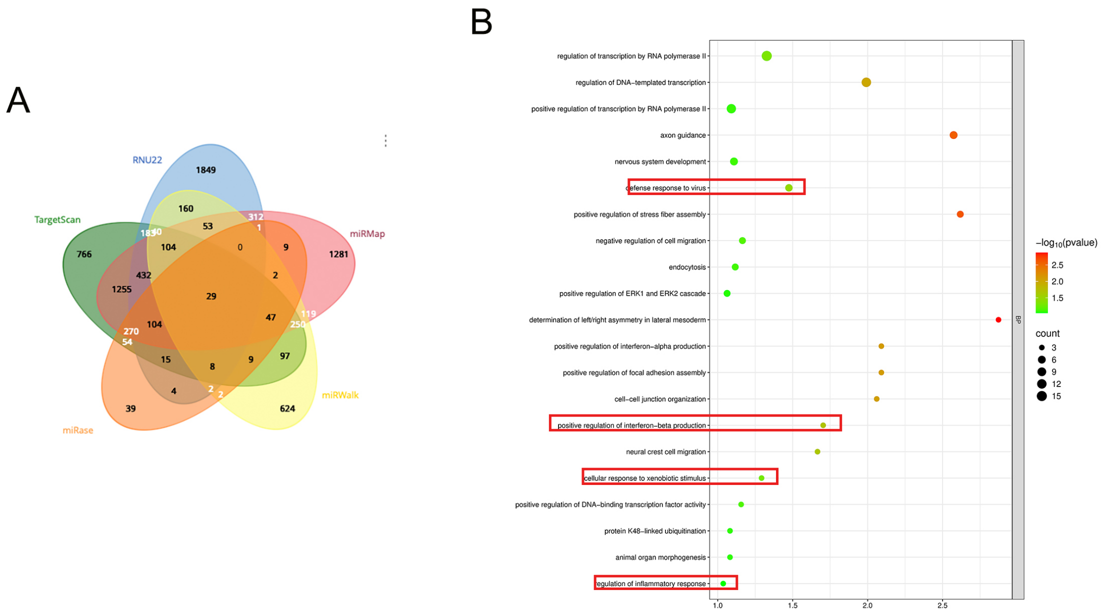

(A) Total numbers of predicted has-miR-21-3p targets. **(B)** GO enrichment analysis of targets of has-miR-21-3p.

Supplementary Figure 3. Protein Array-based profiling of differentially expressed proteins across benign plasma samples groups

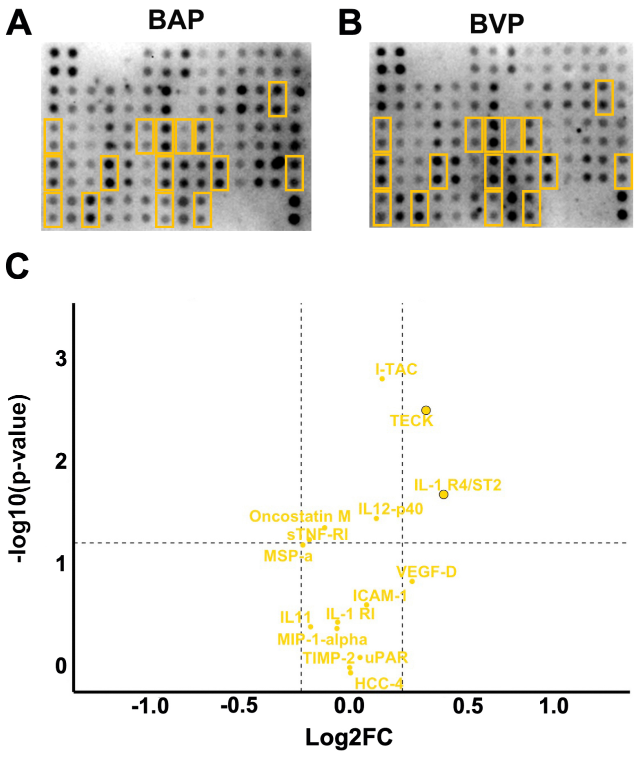

(**A-B)** Human Cytokine Array C7 incubated with six pairs of BAP and BVP plasma samples. Each protein spot was represented in the array by two technical replicates. **(C)** The volcano plot represents the quantification of the cytokine profiles in BAP and BVP plasma samples and is used to summarize the results of the differential analysis. The x-axis shows the $\log_2$ fold change, while the y-axis displays the $-\log_{10}$ (p-value). Each point corresponds to protein analyzed. Statistically significant and increased proteins are indicated by a positive $\log_2$ fold change and a high $-\log_{10}$ (p-value), whereas decreased proteins have a negative $\log_2$ fold change. Genes exceeding the defined threshold values ($p < 0.05$ and, $-0.5 \leq |\log_2$ fold change$| \geq 0.5$) are considered significantly differentially expressed.

Supplementary Figure 4. The percentages of patients with IL-12p40, CCL25, and sTNF-RI concentrations did not differ between benign artery and vein plasma samples

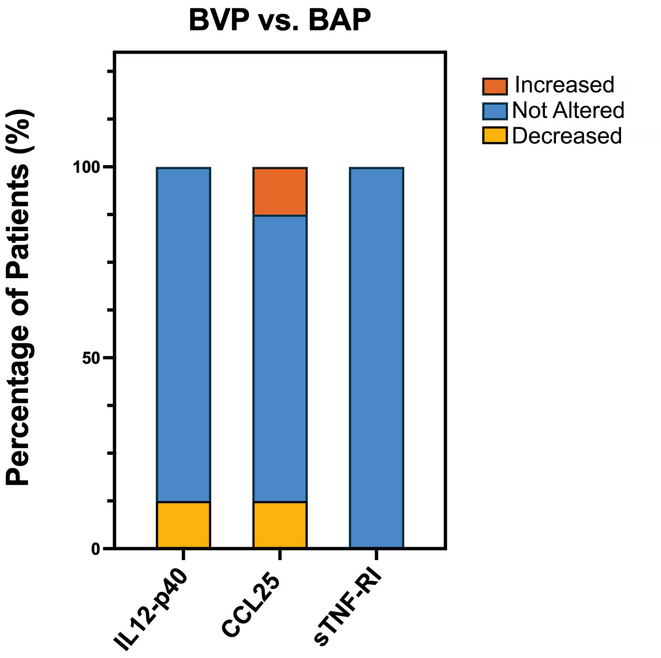

The percentage of patients with IL-12p40, CCL25, and sTNF-RI concentrations in plasma samples from benign arteries and veins.
