# Supplementary material for: Combination of hsa-miR-21-3p/ sTNF-RI/ IL12-p40 /CCL25 serves as a promising panel of diagnostic biomarkers for distinguishing malignant from benign nodules in papillary thyroid cancer
Source: Endocrine. 2026 Apr 27;91(1):146. doi: 10.1007/s12020-026-04612-9 (PMC13121238; doi:10.1007/s12020-026-04612-9)
Supplement: Supplementary file 2 — Supplementary Material 2 [file 12020_2026_4612_MOESM2_ESM.docx]

**Combination of hsa-miR-21-3p/ sTNF-RI/ IL12-p40 /CCL25 serves as a promising panel of diagnostic biomarkers for distinguishing malignant from benign nodules in papillary thyroid cancer.**

Abdulmelik Aytatli^1,2^, Abdulkadir Sahin^3^, Neslisah Barlak^1,2^, Betul Gundogdu^4^, Arzu Tatar^3^, Omer Faruk KARATAS^1,2,*^

**Supplementary File 1.** List of common upregulated and downregulated DE-miRs

1. **Common Upregulated microRNAs (Figure 1D)**

"1" "101111001, GSE191117, GSE113629, GSE103996, GSE73182, GSE63511, GSE159330, hsa-miR-146b-5p, hsa-miR-221-3p, hsa-miR-221-5p, hsa-miR-222-3p, hsa-miR-34a-5p"

"1" "001111011, GSE113629, GSE103996, GSE73182, GSE63511, GSE124653, GSE159330, hsa-miR-146b-5p, hsa-miR-181a-5p, hsa-miR-181b-5p, hsa-miR-21-5p, hsa-miR-221-3p, hsa-miR-221-5p, hsa-miR-34a-5p"

"1" "101111000, GSE191117, GSE113629, GSE103996, GSE73182, GSE63511, hsa-miR-146b-5p, hsa-miR-221-3p, hsa-miR-221-5p, hsa-miR-222-3p, hsa-miR-34a-5p, hsa-miR-551b-3p"

"1" "101110001, GSE191117, GSE113629, GSE103996, GSE73182, GSE159330, hsa-miR-146b-5p, hsa-miR-221-3p, hsa-miR-221-5p, hsa-miR-222-3p, hsa-miR-34a-5p"

"1" "101101001, GSE191117, GSE113629, GSE103996, GSE63511, GSE159330, hsa-miR-146b-5p, hsa-miR-221-3p, hsa-miR-221-5p, hsa-miR-222-3p, hsa-miR-34a-5p"

"1" "101011001, GSE191117, GSE113629, GSE73182, GSE63511, GSE159330, hsa-miR-146b-5p, hsa-miR-221-3p, hsa-miR-221-5p, hsa-miR-222-3p, hsa-miR-34a-5p"

"1" "100111001, GSE191117, GSE103996, GSE73182, GSE63511, GSE159330, hsa-miR-146b-5p, hsa-miR-221-3p, hsa-miR-221-5p, hsa-miR-222-3p, hsa-miR-34a-5p"

"1" "100110011, GSE191117, GSE103996, GSE73182, GSE124653, GSE159330, hsa-miR-146b-5p, hsa-miR-155-5p, hsa-miR-221-3p, hsa-miR-221-5p, hsa-miR-34a-5p"

"1" "011011001, GSE151180, GSE113629, GSE73182, GSE63511, GSE159330, hsa-miR-146b-5p, hsa-miR-21-3p, hsa-miR-21-5p, hsa-miR-221-3p, hsa-miR-222-3p"

"1" "001111010, GSE113629, GSE103996, GSE73182, GSE63511, GSE124653, hsa-miR-146b-5p, hsa-miR-181a-5p, hsa-miR-181b-5p, hsa-miR-21-5p, hsa-miR-221-3p, hsa-miR-221-5p, hsa-miR-34a-5p"

"1" "001111001, GSE113629, GSE103996, GSE73182, GSE63511, GSE159330, hsa-miR-146b-5p, hsa-miR-181a-5p, hsa-miR-181b-5p, hsa-miR-21-5p, hsa-miR-221-3p, hsa-miR-221-5p, hsa-miR-222-3p, hsa-miR-34a-5p"

"1" "001110011, GSE113629, GSE103996, GSE73182, GSE124653, GSE159330, hsa-miR-146b-5p, hsa-miR-181a-5p, hsa-miR-181b-5p, hsa-miR-21-5p, hsa-miR-221-3p, hsa-miR-221-5p, hsa-miR-34a-5p"

"1" "001101011, GSE113629, GSE103996, GSE63511, GSE124653, GSE159330, hsa-miR-146b-3p, hsa-miR-146b-5p, hsa-miR-181a-5p, hsa-miR-181b-5p, hsa-miR-21-5p, hsa-miR-221-3p, hsa-miR-221-5p, hsa-miR-34a-5p"

"1" "001011011, GSE113629, GSE73182, GSE63511, GSE124653, GSE159330, hsa-miR-146b-5p, hsa-miR-181a-5p, hsa-miR-181b-5p, hsa-miR-21-3p, hsa-miR-21-5p, hsa-miR-221-3p, hsa-miR-221-5p, hsa-miR-34a-5p"

"1" "000111011, GSE103996, GSE73182, GSE63511, GSE124653, GSE159330, hsa-miR-146b-5p, hsa-miR-181a-5p, hsa-miR-181b-5p, hsa-miR-21-5p, hsa-miR-221-3p, hsa-miR-221-5p, hsa-miR-34a-5p"

"x"

"1" "101110000, GSE191117, GSE113629, GSE103996, GSE73182, hsa-miR-146b-5p, hsa-miR-221-3p, hsa-miR-221-5p, hsa-miR-222-3p, hsa-miR-34a-5p, hsa-miR-551b-3p"

"1" "101101000, GSE191117, GSE113629, GSE103996, GSE63511, hsa-miR-146b-5p, hsa-miR-221-3p, hsa-miR-221-5p, hsa-miR-222-3p, hsa-miR-34a-5p, hsa-miR-551b-3p"

"1" "101100001, GSE191117, GSE113629, GSE103996, GSE159330, hsa-miR-146b-5p, hsa-miR-221-3p, hsa-miR-221-5p, hsa-miR-222-3p, hsa-miR-34a-5p"

"1" "101011000, GSE191117, GSE113629, GSE73182, GSE63511, hsa-miR-146b-5p, hsa-miR-221-3p, hsa-miR-221-5p, hsa-miR-222-3p, hsa-miR-34a-5p, hsa-miR-551b-3p"

"1" "101010001, GSE191117, GSE113629, GSE73182, GSE159330, hsa-miR-146b-5p, hsa-miR-221-3p, hsa-miR-221-5p, hsa-miR-222-3p, hsa-miR-34a-5p"

"1" "101001001, GSE191117, GSE113629, GSE63511, GSE159330, hsa-miR-146b-5p, hsa-miR-221-3p, hsa-miR-221-5p, hsa-miR-222-3p, hsa-miR-34a-5p"

"1" "100111000, GSE191117, GSE103996, GSE73182, GSE63511, hsa-miR-146b-5p, hsa-miR-221-3p, hsa-miR-221-5p, hsa-miR-222-3p, hsa-miR-34a-5p, hsa-miR-551b-3p"

"1" "100110010, GSE191117, GSE103996, GSE73182, GSE124653, hsa-miR-146b-5p, hsa-miR-155-5p, hsa-miR-221-3p, hsa-miR-221-5p, hsa-miR-34a-5p"

"1" "100110001, GSE191117, GSE103996, GSE73182, GSE159330, hsa-miR-146b-5p, hsa-miR-155-5p, hsa-miR-221-3p, hsa-miR-221-5p, hsa-miR-222-3p, hsa-miR-34a-5p"

"1" "100101001, GSE191117, GSE103996, GSE63511, GSE159330, hsa-miR-146b-5p, hsa-miR-221-3p, hsa-miR-221-5p, hsa-miR-222-3p, hsa-miR-34a-5p"

"1" "100100011, GSE191117, GSE103996, GSE124653, GSE159330, hsa-miR-146b-5p, hsa-miR-155-5p, hsa-miR-221-3p, hsa-miR-221-5p, hsa-miR-34a-5p"

"1" "100011001, GSE191117, GSE73182, GSE63511, GSE159330, hsa-miR-146b-5p, hsa-miR-221-3p, hsa-miR-221-5p, hsa-miR-222-3p, hsa-miR-34a-5p"

"1" "100010011, GSE191117, GSE73182, GSE124653, GSE159330, hsa-miR-146b-5p, hsa-miR-155-5p, hsa-miR-221-3p, hsa-miR-221-5p, hsa-miR-34a-5p"

"1" "011011000, GSE151180, GSE113629, GSE73182, GSE63511, hsa-miR-146b-5p, hsa-miR-21-3p, hsa-miR-21-5p, hsa-miR-221-3p, hsa-miR-222-3p"

"1" "011010001, GSE151180, GSE113629, GSE73182, GSE159330, hsa-miR-146b-5p, hsa-miR-21-3p, hsa-miR-21-5p, hsa-miR-221-3p, hsa-miR-222-3p"

"1" "011001001, GSE151180, GSE113629, GSE63511, GSE159330, hsa-miR-146b-5p, hsa-miR-21-3p, hsa-miR-21-5p, hsa-miR-221-3p, hsa-miR-222-3p"

"1" "010011001, GSE151180, GSE73182, GSE63511, GSE159330, hsa-miR-146b-5p, hsa-miR-21-3p, hsa-miR-21-5p, hsa-miR-221-3p, hsa-miR-222-3p"

"1" "001111000, GSE113629, GSE103996, GSE73182, GSE63511, hsa-miR-146b-5p, hsa-miR-181a-5p, hsa-miR-181b-5p, hsa-miR-21-5p, hsa-miR-221-3p, hsa-miR-221-5p, hsa-miR-222-3p, hsa-miR-34a-5p, hsa-miR-551b-3p"

"1" "001110010, GSE113629, GSE103996, GSE73182, GSE124653, hsa-miR-146b-5p, hsa-miR-15a-5p, hsa-miR-181a-5p, hsa-miR-181b-5p, hsa-miR-21-5p, hsa-miR-221-3p, hsa-miR-221-5p, hsa-miR-34a-5p"

"1" "001110001, GSE113629, GSE103996, GSE73182, GSE159330, hsa-miR-146b-5p, hsa-miR-181a-5p, hsa-miR-181b-5p, hsa-miR-21-5p, hsa-miR-221-3p, hsa-miR-221-5p, hsa-miR-222-3p, hsa-miR-34a-5p"

"1" "001101010, GSE113629, GSE103996, GSE63511, GSE124653, hsa-miR-146b-3p, hsa-miR-146b-5p, hsa-miR-181a-5p, hsa-miR-181b-5p, hsa-miR-21-5p, hsa-miR-221-3p, hsa-miR-221-5p, hsa-miR-34a-5p"

"1" "001101001, GSE113629, GSE103996, GSE63511, GSE159330, hsa-miR-146b-3p, hsa-miR-146b-5p, hsa-miR-181a-2-3p, hsa-miR-181a-5p, hsa-miR-181b-5p, hsa-miR-182-5p, hsa-miR-21-5p, hsa-miR-221-3p, hsa-miR-221-5p, hsa-miR-222-3p, hsa-miR-34a-5p"

"1" "001100011, GSE113629, GSE103996, GSE124653, GSE159330, hsa-miR-146b-3p, hsa-miR-146b-5p, hsa-miR-181a-5p, hsa-miR-181b-5p, hsa-miR-21-5p, hsa-miR-221-3p, hsa-miR-221-5p, hsa-miR-34a-5p"

"1" "001011010, GSE113629, GSE73182, GSE63511, GSE124653, hsa-miR-146b-5p, hsa-miR-181a-5p, hsa-miR-181b-5p, hsa-miR-21-3p, hsa-miR-21-5p, hsa-miR-221-3p, hsa-miR-221-5p, hsa-miR-34a-5p"

"1" "001011001, GSE113629, GSE73182, GSE63511, GSE159330, hsa-miR-146b-5p, hsa-miR-181a-5p, hsa-miR-181b-5p, hsa-miR-21-3p, hsa-miR-21-5p, hsa-miR-221-3p, hsa-miR-221-5p, hsa-miR-222-3p, hsa-miR-34a-5p"

"1" "001010011, GSE113629, GSE73182, GSE124653, GSE159330, hsa-miR-146b-5p, hsa-miR-181a-5p, hsa-miR-181b-5p, hsa-miR-21-3p, hsa-miR-21-5p, hsa-miR-221-3p, hsa-miR-221-5p, hsa-miR-34a-5p"

"1" "001001011, GSE113629, GSE63511, GSE124653, GSE159330, hsa-miR-146b-3p, hsa-miR-146b-5p, hsa-miR-181a-5p, hsa-miR-181b-5p, hsa-miR-21-3p, hsa-miR-21-5p, hsa-miR-221-3p, hsa-miR-221-5p, hsa-miR-34a-5p"

"1" "000111010, GSE103996, GSE73182, GSE63511, GSE124653, hsa-miR-146b-5p, hsa-miR-181a-5p, hsa-miR-181b-5p, hsa-miR-21-5p, hsa-miR-221-3p, hsa-miR-221-5p, hsa-miR-34a-5p"

"1" "000111001, GSE103996, GSE73182, GSE63511, GSE159330, hsa-miR-146b-5p, hsa-miR-181a-5p, hsa-miR-181b-5p, hsa-miR-21-5p, hsa-miR-221-3p, hsa-miR-221-5p, hsa-miR-222-3p, hsa-miR-34a-5p"

"1" "000110011, GSE103996, GSE73182, GSE124653, GSE159330, hsa-miR-146b-5p, hsa-miR-155-5p, hsa-miR-181a-5p, hsa-miR-181b-5p, hsa-miR-21-5p, hsa-miR-221-3p, hsa-miR-221-5p, hsa-miR-34a-5p"

"1" "000101011, GSE103996, GSE63511, GSE124653, GSE159330, hsa-miR-146b-3p, hsa-miR-146b-5p, hsa-miR-181a-5p, hsa-miR-181b-5p, hsa-miR-21-5p, hsa-miR-221-3p, hsa-miR-221-5p, hsa-miR-34a-5p"

"1" "000011011, GSE73182, GSE63511, GSE124653, GSE159330, hsa-miR-146b-5p, hsa-miR-181a-5p, hsa-miR-181b-5p, hsa-miR-21-3p, hsa-miR-21-5p, hsa-miR-221-3p, hsa-miR-221-5p, hsa-miR-34a-5p"

"1" "101100000, GSE191117, GSE113629, GSE103996, hsa-miR-146b-5p, hsa-miR-221-3p, hsa-miR-221-5p, hsa-miR-222-3p, hsa-miR-34a-5p, hsa-miR-551b-3p"

"1" "101010000, GSE191117, GSE113629, GSE73182, hsa-miR-146b-5p, hsa-miR-221-3p, hsa-miR-221-5p, hsa-miR-222-3p, hsa-miR-34a-5p, hsa-miR-551b-3p"

"1" "101001000, GSE191117, GSE113629, GSE63511, hsa-miR-146b-5p, hsa-miR-221-3p, hsa-miR-221-5p, hsa-miR-222-3p, hsa-miR-31-5p, hsa-miR-34a-5p, hsa-miR-551b-3p"

"1" "101000001, GSE191117, GSE113629, GSE159330, hsa-miR-146b-5p, hsa-miR-221-3p, hsa-miR-221-5p, hsa-miR-222-3p, hsa-miR-34a-5p"

"1" "100110000, GSE191117, GSE103996, GSE73182, hsa-miR-146b-5p, hsa-miR-155-5p, hsa-miR-221-3p, hsa-miR-221-5p, hsa-miR-222-3p, hsa-miR-34a-5p, hsa-miR-551b-3p"

"1" "100101000, GSE191117, GSE103996, GSE63511, hsa-miR-146b-5p, hsa-miR-221-3p, hsa-miR-221-5p, hsa-miR-222-3p, hsa-miR-34a-5p, hsa-miR-551b-3p"

"1" "100100010, GSE191117, GSE103996, GSE124653, hsa-miR-142-3p, hsa-miR-146b-5p, hsa-miR-155-5p, hsa-miR-221-3p, hsa-miR-221-5p, hsa-miR-34a-5p"

"1" "100100001, GSE191117, GSE103996, GSE159330, hsa-miR-146b-5p, hsa-miR-155-5p, hsa-miR-221-3p, hsa-miR-221-5p, hsa-miR-222-3p, hsa-miR-34a-5p"

"1" "100011000, GSE191117, GSE73182, GSE63511, hsa-miR-146b-5p, hsa-miR-221-3p, hsa-miR-221-5p, hsa-miR-222-3p, hsa-miR-34a-5p, hsa-miR-551b-3p"

"1" "100010010, GSE191117, GSE73182, GSE124653, hsa-miR-146b-5p, hsa-miR-155-5p, hsa-miR-221-3p, hsa-miR-221-5p, hsa-miR-34a-5p"

"1" "100010001, GSE191117, GSE73182, GSE159330, hsa-miR-146b-5p, hsa-miR-155-5p, hsa-miR-221-3p, hsa-miR-221-5p, hsa-miR-222-3p, hsa-miR-34a-5p"

"1" "100001001, GSE191117, GSE63511, GSE159330, hsa-miR-146b-5p, hsa-miR-221-3p, hsa-miR-221-5p, hsa-miR-222-3p, hsa-miR-34a-5p"

"1" "100000011, GSE191117, GSE124653, GSE159330, hsa-miR-146b-5p, hsa-miR-155-5p, hsa-miR-221-3p, hsa-miR-221-5p, hsa-miR-34a-5p"

"1" "011010000, GSE151180, GSE113629, GSE73182, hsa-miR-146b-5p, hsa-miR-21-3p, hsa-miR-21-5p, hsa-miR-221-3p, hsa-miR-222-3p"

"1" "011001000, GSE151180, GSE113629, GSE63511, hsa-miR-146b-5p, hsa-miR-21-3p, hsa-miR-21-5p, hsa-miR-221-3p, hsa-miR-222-3p, hsa-miR-31-5p"

"1" "011000001, GSE151180, GSE113629, GSE159330, hsa-miR-146b-5p, hsa-miR-21-3p, hsa-miR-21-5p, hsa-miR-221-3p, hsa-miR-222-3p"

"1" "010011000, GSE151180, GSE73182, GSE63511, hsa-miR-146b-5p, hsa-miR-21-3p, hsa-miR-21-5p, hsa-miR-221-3p, hsa-miR-222-3p"

"1" "010010001, GSE151180, GSE73182, GSE159330, hsa-miR-146b-5p, hsa-miR-21-3p, hsa-miR-21-5p, hsa-miR-221-3p, hsa-miR-222-3p"

"1" "010001001, GSE151180, GSE63511, GSE159330, hsa-miR-146b-5p, hsa-miR-21-3p, hsa-miR-21-5p, hsa-miR-221-3p, hsa-miR-222-3p"

"1" "001110000, GSE113629, GSE103996, GSE73182, hsa-miR-146b-5p, hsa-miR-15a-5p, hsa-miR-181a-5p, hsa-miR-181b-5p, hsa-miR-21-5p, hsa-miR-221-3p, hsa-miR-221-5p, hsa-miR-222-3p, hsa-miR-34a-5p, hsa-miR-34b-5p, hsa-miR-551b-3p"

"1" "001101000, GSE113629, GSE103996, GSE63511, hsa-miR-146b-3p, hsa-miR-146b-5p, hsa-miR-181a-2-3p, hsa-miR-181a-5p, hsa-miR-181b-5p, hsa-miR-182-5p, hsa-miR-21-5p, hsa-miR-221-3p, hsa-miR-221-5p, hsa-miR-222-3p, hsa-miR-34a-5p, hsa-miR-551b-3p"

"1" "001100010, GSE113629, GSE103996, GSE124653, hsa-miR-146b-3p, hsa-miR-146b-5p, hsa-miR-15a-5p, hsa-miR-181a-5p, hsa-miR-181b-5p, hsa-miR-21-5p, hsa-miR-221-3p, hsa-miR-221-5p, hsa-miR-34a-5p"

"1" "001100001, GSE113629, GSE103996, GSE159330, hsa-miR-146b-3p, hsa-miR-146b-5p, hsa-miR-181a-2-3p, hsa-miR-181a-5p, hsa-miR-181b-5p, hsa-miR-182-5p, hsa-miR-21-5p, hsa-miR-221-3p, hsa-miR-221-5p, hsa-miR-222-3p, hsa-miR-34a-5p"

"1" "001011000, GSE113629, GSE73182, GSE63511, hsa-miR-146b-5p, hsa-miR-181a-5p, hsa-miR-181b-5p, hsa-miR-21-3p, hsa-miR-21-5p, hsa-miR-221-3p, hsa-miR-221-5p, hsa-miR-222-3p, hsa-miR-34a-5p, hsa-miR-551b-3p"

"1" "001010010, GSE113629, GSE73182, GSE124653, hsa-miR-141-3p, hsa-miR-146b-5p, hsa-miR-15a-5p, hsa-miR-181a-5p, hsa-miR-181b-5p, hsa-miR-21-3p, hsa-miR-21-5p, hsa-miR-221-3p, hsa-miR-221-5p, hsa-miR-34a-5p"

"1" "001010001, GSE113629, GSE73182, GSE159330, hsa-miR-146b-5p, hsa-miR-181a-5p, hsa-miR-181b-5p, hsa-miR-21-3p, hsa-miR-21-5p, hsa-miR-221-3p, hsa-miR-221-5p, hsa-miR-222-3p, hsa-miR-34a-5p"

"1" "001001010, GSE113629, GSE63511, GSE124653, hsa-miR-146b-3p, hsa-miR-146b-5p, hsa-miR-181a-5p, hsa-miR-181b-5p, hsa-miR-21-3p, hsa-miR-21-5p, hsa-miR-221-3p, hsa-miR-221-5p, hsa-miR-34a-5p"

"1" "001001001, GSE113629, GSE63511, GSE159330, hsa-let-7e-5p, hsa-miR-146b-3p, hsa-miR-146b-5p, hsa-miR-181a-2-3p, hsa-miR-181a-5p, hsa-miR-181b-5p, hsa-miR-182-5p, hsa-miR-21-3p, hsa-miR-21-5p, hsa-miR-221-3p, hsa-miR-221-5p, hsa-miR-222-3p, hsa-miR-34a-5p"

"1" "001000110, GSE113629, GSE116196, GSE124653, hsa-miR-146b-3p, hsa-miR-146b-5p, hsa-miR-221-5p, hsa-miR-34a-5p, hsa-miR-424-5p"

"1" "001000011, GSE113629, GSE124653, GSE159330, hsa-miR-146b-3p, hsa-miR-146b-5p, hsa-miR-181a-3p, hsa-miR-181a-5p, hsa-miR-181b-5p, hsa-miR-21-3p, hsa-miR-21-5p, hsa-miR-221-3p, hsa-miR-221-5p, hsa-miR-34a-5p"

"1" "000111000, GSE103996, GSE73182, GSE63511, hsa-miR-146b-5p, hsa-miR-181a-5p, hsa-miR-181b-5p, hsa-miR-21-5p, hsa-miR-221-3p, hsa-miR-221-5p, hsa-miR-222-3p, hsa-miR-34a-5p, hsa-miR-551b-3p"

"1" "000110010, GSE103996, GSE73182, GSE124653, hsa-miR-146b-5p, hsa-miR-155-5p, hsa-miR-15a-5p, hsa-miR-181a-5p, hsa-miR-181b-5p, hsa-miR-21-5p, hsa-miR-221-3p, hsa-miR-221-5p, hsa-miR-34a-5p"

"1" "000110001, GSE103996, GSE73182, GSE159330, hsa-miR-146b-5p, hsa-miR-155-5p, hsa-miR-181a-5p, hsa-miR-181b-5p, hsa-miR-21-5p, hsa-miR-221-3p, hsa-miR-221-5p, hsa-miR-222-3p, hsa-miR-34a-5p"

"1" "000101010, GSE103996, GSE63511, GSE124653, hsa-miR-146b-3p, hsa-miR-146b-5p, hsa-miR-181a-5p, hsa-miR-181b-5p, hsa-miR-21-5p, hsa-miR-221-3p, hsa-miR-221-5p, hsa-miR-34a-5p"

"1" "000101001, GSE103996, GSE63511, GSE159330, hsa-miR-146b-3p, hsa-miR-146b-5p, hsa-miR-181a-2-3p, hsa-miR-181a-5p, hsa-miR-181b-5p, hsa-miR-182-5p, hsa-miR-21-5p, hsa-miR-221-3p, hsa-miR-221-5p, hsa-miR-222-3p, hsa-miR-34a-5p"

"1" "000100011, GSE103996, GSE124653, GSE159330, hsa-miR-146b-3p, hsa-miR-146b-5p, hsa-miR-155-5p, hsa-miR-181a-5p, hsa-miR-181b-5p, hsa-miR-21-5p, hsa-miR-221-3p, hsa-miR-221-5p, hsa-miR-34a-5p"

"1" "000011010, GSE73182, GSE63511, GSE124653, hsa-miR-146b-5p, hsa-miR-181a-5p, hsa-miR-181b-5p, hsa-miR-21-3p, hsa-miR-21-5p, hsa-miR-221-3p, hsa-miR-221-5p, hsa-miR-34a-5p"

"1" "000011001, GSE73182, GSE63511, GSE159330, hsa-miR-146b-5p, hsa-miR-181a-5p, hsa-miR-181b-5p, hsa-miR-21-3p, hsa-miR-21-5p, hsa-miR-221-3p, hsa-miR-221-5p, hsa-miR-222-3p, hsa-miR-34a-5p"

"1" "000010011, GSE73182, GSE124653, GSE159330, hsa-miR-146b-5p, hsa-miR-155-5p, hsa-miR-181a-5p, hsa-miR-181b-5p, hsa-miR-21-3p, hsa-miR-21-5p, hsa-miR-221-3p, hsa-miR-221-5p, hsa-miR-34a-5p"

"1" "000001011, GSE63511, GSE124653, GSE159330, hsa-miR-146b-3p, hsa-miR-146b-5p, hsa-miR-181a-5p, hsa-miR-181b-5p, hsa-miR-21-3p, hsa-miR-21-5p, hsa-miR-221-3p, hsa-miR-221-5p, hsa-miR-34a-5p"

"1" "101000000, GSE191117, GSE113629, hsa-miR-1295a, hsa-miR-146b-5p, hsa-miR-221-3p, hsa-miR-221-5p, hsa-miR-222-3p, hsa-miR-31-5p, hsa-miR-34a-5p, hsa-miR-551b-3p"

"1" "100100000, GSE191117, GSE103996, hsa-miR-142-3p, hsa-miR-146b-5p, hsa-miR-155-5p, hsa-miR-221-3p, hsa-miR-221-5p, hsa-miR-222-3p, hsa-miR-34a-5p, hsa-miR-551b-3p"

"1" "100010000, GSE191117, GSE73182, hsa-miR-146b-5p, hsa-miR-155-5p, hsa-miR-221-3p, hsa-miR-221-5p, hsa-miR-222-3p, hsa-miR-34a-5p, hsa-miR-551b-3p"

"1" "100001000, GSE191117, GSE63511, hsa-miR-146b-5p, hsa-miR-221-3p, hsa-miR-221-5p, hsa-miR-222-3p, hsa-miR-31-5p, hsa-miR-34a-5p, hsa-miR-551b-3p"

"1" "100000010, GSE191117, GSE124653, hsa-miR-142-3p, hsa-miR-146a-5p, hsa-miR-146b-5p, hsa-miR-155-5p, hsa-miR-221-3p, hsa-miR-221-5p, hsa-miR-34a-5p"

"1" "100000001, GSE191117, GSE159330, hsa-miR-146b-5p, hsa-miR-155-5p, hsa-miR-221-3p, hsa-miR-221-5p, hsa-miR-222-3p, hsa-miR-34a-5p"

"1" "011000000, GSE151180, GSE113629, hsa-miR-146b-5p, hsa-miR-21-3p, hsa-miR-21-5p, hsa-miR-221-3p, hsa-miR-222-3p, hsa-miR-31-5p, hsa-miR-4446-3p, hsa-miR-4713-3p, hsa-miR-4769-5p, hsa-miR-513b-5p, hsa-miR-574-5p, hsa-miR-6875-5p"

"1" "010010000, GSE151180, GSE73182, hsa-miR-146b-5p, hsa-miR-21-3p, hsa-miR-21-5p, hsa-miR-221-3p, hsa-miR-222-3p"

"1" "010001000, GSE151180, GSE63511, hsa-miR-146b-5p, hsa-miR-21-3p, hsa-miR-21-5p, hsa-miR-221-3p, hsa-miR-222-3p, hsa-miR-31-5p"

"1" "010000001, GSE151180, GSE159330, hsa-miR-146b-5p, hsa-miR-21-3p, hsa-miR-21-5p, hsa-miR-221-3p, hsa-miR-222-3p"

"1" "001100000, GSE113629, GSE103996, hsa-miR-146b-3p, hsa-miR-146b-5p, hsa-miR-15a-5p, hsa-miR-181a-2-3p, hsa-miR-181a-5p, hsa-miR-181b-5p, hsa-miR-182-5p, hsa-miR-202-3p, hsa-miR-21-5p, hsa-miR-221-3p, hsa-miR-221-5p, hsa-miR-222-3p, hsa-miR-34a-5p, hsa-miR-34b-5p, hsa-miR-551b-3p"

"1" "001010000, GSE113629, GSE73182, hsa-miR-141-3p, hsa-miR-146b-5p, hsa-miR-15a-5p, hsa-miR-181a-5p, hsa-miR-181b-5p, hsa-miR-21-3p, hsa-miR-21-5p, hsa-miR-221-3p, hsa-miR-221-5p, hsa-miR-222-3p, hsa-miR-34a-5p, hsa-miR-34b-5p, hsa-miR-551b-3p"

"1" "001001000, GSE113629, GSE63511, hsa-let-7e-5p, hsa-miR-146b-3p, hsa-miR-146b-5p, hsa-miR-181a-2-3p, hsa-miR-181a-5p, hsa-miR-181b-5p, hsa-miR-182-5p, hsa-miR-21-3p, hsa-miR-21-5p, hsa-miR-221-3p, hsa-miR-221-5p, hsa-miR-222-3p, hsa-miR-31-3p, hsa-miR-31-5p, hsa-miR-34a-5p, hsa-miR-3613-5p, hsa-miR-551b-3p, hsa-miR-96-5p"

"1" "001000100, GSE113629, GSE116196, hsa-miR-122-5p, hsa-miR-146b-3p, hsa-miR-146b-5p, hsa-miR-221-5p, hsa-miR-222-5p, hsa-miR-34a-5p, hsa-miR-375-5p, hsa-miR-424-5p"

"1" "001000010, GSE113629, GSE124653, hsa-let-7i-3p, hsa-let-7i-5p, hsa-miR-101-3p, hsa-miR-106b-5p, hsa-miR-141-3p, hsa-miR-146b-3p, hsa-miR-146b-5p, hsa-miR-15a-5p, hsa-miR-181a-3p, hsa-miR-181a-5p, hsa-miR-181b-3p, hsa-miR-181b-5p, hsa-miR-19b-3p, hsa-miR-20a-5p, hsa-miR-21-3p, hsa-miR-21-5p, hsa-miR-221-3p, hsa-miR-221-5p, hsa-miR-27a-3p, hsa-miR-27b-3p, hsa-miR-29a-3p, hsa-miR-29b-3p, hsa-miR-29c-3p, hsa-miR-32-5p, hsa-miR-340-5p, hsa-miR-34a-5p, hsa-miR-374a-3p, hsa-miR-374a-5p, hsa-miR-424-5p, hsa-miR-503-5p, hsa-miR-542-3p, hsa-miR-590-3p"

"1" "001000001, GSE113629, GSE159330, hsa-let-7e-5p, hsa-miR-146b-3p, hsa-miR-146b-5p, hsa-miR-181a-2-3p, hsa-miR-181a-3p, hsa-miR-181a-5p, hsa-miR-181b-5p, hsa-miR-182-5p, hsa-miR-21-3p, hsa-miR-21-5p, hsa-miR-221-3p, hsa-miR-221-5p, hsa-miR-222-3p, hsa-miR-34a-5p, hsa-miR-92b-3p"

"1" "000110000, GSE103996, GSE73182, hsa-miR-142-5p, hsa-miR-146b-5p, hsa-miR-155-5p, hsa-miR-15a-5p, hsa-miR-181a-5p, hsa-miR-181b-5p, hsa-miR-21-5p, hsa-miR-221-3p, hsa-miR-221-5p, hsa-miR-222-3p, hsa-miR-34a-5p, hsa-miR-34b-5p, hsa-miR-551b-3p"

"1" "000101000, GSE103996, GSE63511, hsa-miR-146b-3p, hsa-miR-146b-5p, hsa-miR-181a-2-3p, hsa-miR-181a-5p, hsa-miR-181b-5p, hsa-miR-182-5p, hsa-miR-21-5p, hsa-miR-221-3p, hsa-miR-221-5p, hsa-miR-222-3p, hsa-miR-34a-5p, hsa-miR-551b-3p"

"1" "000100010, GSE103996, GSE124653, hsa-miR-142-3p, hsa-miR-146b-3p, hsa-miR-146b-5p, hsa-miR-155-5p, hsa-miR-15a-5p, hsa-miR-181a-5p, hsa-miR-181b-5p, hsa-miR-21-5p, hsa-miR-221-3p, hsa-miR-221-5p, hsa-miR-34a-5p"

"1" "000100001, GSE103996, GSE159330, hsa-miR-146b-3p, hsa-miR-146b-5p, hsa-miR-155-5p, hsa-miR-181a-2-3p, hsa-miR-181a-5p, hsa-miR-181b-5p, hsa-miR-182-5p, hsa-miR-21-5p, hsa-miR-221-3p, hsa-miR-221-5p, hsa-miR-222-3p, hsa-miR-34a-5p"

"1" "000011000, GSE73182, GSE63511, hsa-miR-146b-5p, hsa-miR-181a-5p, hsa-miR-181b-5p, hsa-miR-21-3p, hsa-miR-21-5p, hsa-miR-221-3p, hsa-miR-221-5p, hsa-miR-222-3p, hsa-miR-34a-5p, hsa-miR-551b-3p"

"1" "000010010, GSE73182, GSE124653, hsa-miR-141-3p, hsa-miR-146b-5p, hsa-miR-155-5p, hsa-miR-15a-5p, hsa-miR-181a-5p, hsa-miR-181b-5p, hsa-miR-21-3p, hsa-miR-21-5p, hsa-miR-221-3p, hsa-miR-221-5p, hsa-miR-34a-5p"

"1" "000010001, GSE73182, GSE159330, hsa-miR-146b-5p, hsa-miR-155-5p, hsa-miR-181a-5p, hsa-miR-181b-5p, hsa-miR-21-3p, hsa-miR-21-5p, hsa-miR-221-3p, hsa-miR-221-5p, hsa-miR-222-3p, hsa-miR-34a-5p"

"1" "000001010, GSE63511, GSE124653, hsa-miR-146b-3p, hsa-miR-146b-5p, hsa-miR-181a-5p, hsa-miR-181b-5p, hsa-miR-21-3p, hsa-miR-21-5p, hsa-miR-221-3p, hsa-miR-221-5p, hsa-miR-34a-5p"

"1" "000001001, GSE63511, GSE159330, hsa-let-7e-5p, hsa-miR-146b-3p, hsa-miR-146b-5p, hsa-miR-181a-2-3p, hsa-miR-181a-5p, hsa-miR-181b-5p, hsa-miR-182-5p, hsa-miR-183-5p, hsa-miR-21-3p, hsa-miR-21-5p, hsa-miR-221-3p, hsa-miR-221-5p, hsa-miR-222-3p, hsa-miR-34a-5p"

"1" "000000110, GSE116196, GSE124653, hsa-miR-146b-3p, hsa-miR-146b-5p, hsa-miR-221-5p, hsa-miR-34a-5p, hsa-miR-424-5p"

"1" "000000011, GSE124653, GSE159330, hsa-miR-146b-3p, hsa-miR-146b-5p, hsa-miR-155-5p, hsa-miR-181a-3p, hsa-miR-181a-5p, hsa-miR-181b-5p, hsa-miR-21-3p, hsa-miR-21-5p, hsa-miR-221-3p, hsa-miR-221-5p, hsa-miR-34a-5p"

1. **Common Downregulated mikroRNAs (Figure 1E)**

x"

"1" "010111001, GSE151180, GSE103996, GSE73182, GSE63511, GSE159330, hsa-miR-100-5p, hsa-miR-138-5p, hsa-miR-144-3p, hsa-miR-199b-5p, hsa-miR-204-5p, hsa-miR-30a-3p, hsa-miR-451a, hsa-miR-99a-5p"

"x"

"1" "011101000, GSE151180, GSE113629, GSE103996, GSE63511, hsa-miR-144-3p, hsa-miR-144-5p, hsa-miR-204-5p, hsa-miR-451a, hsa-miR-486-5p, hsa-miR-7-2-3p, hsa-miR-7-5p"

"x"

"1" "011001001, GSE151180, GSE113629, GSE63511, GSE159330, hsa-miR-144-3p, hsa-miR-144-5p, hsa-miR-204-5p, hsa-miR-363-3p, hsa-miR-451a"

"x"

"1" "010111000, GSE151180, GSE103996, GSE73182, GSE63511, hsa-miR-100-5p, hsa-miR-138-5p, hsa-miR-144-3p, hsa-miR-199b-5p, hsa-miR-204-5p, hsa-miR-30a-3p, hsa-miR-451a, hsa-miR-7-5p, hsa-miR-99a-5p"

"x"

"1" "010110001, GSE151180, GSE103996, GSE73182, GSE159330, hsa-miR-100-5p, hsa-miR-126-3p, hsa-miR-130a-3p, hsa-miR-138-5p, hsa-miR-144-3p, hsa-miR-199b-5p, hsa-miR-204-5p, hsa-miR-30a-3p, hsa-miR-451a, hsa-miR-99a-5p"

"x"

"1" "010101001, GSE151180, GSE103996, GSE63511, GSE159330, hsa-miR-100-5p, hsa-miR-138-5p, hsa-miR-144-3p, hsa-miR-144-5p, hsa-miR-199b-5p, hsa-miR-204-5p, hsa-miR-30a-3p, hsa-miR-30a-5p, hsa-miR-451a, hsa-miR-99a-5p"

"x"

"1" "010011001, GSE151180, GSE73182, GSE63511, GSE159330, hsa-miR-100-5p, hsa-miR-138-5p, hsa-miR-144-3p, hsa-miR-199b-5p, hsa-miR-204-5p, hsa-miR-30a-3p, hsa-miR-451a, hsa-miR-99a-5p"

"x"

"1" "010001011, GSE151180, GSE63511, GSE124653, GSE159330, hsa-miR-144-5p, hsa-miR-145-5p, hsa-miR-363-3p, hsa-miR-451a, hsa-miR-652-3p"

"x"

"1" "000111001, GSE103996, GSE73182, GSE63511, GSE159330, hsa-miR-100-5p, hsa-miR-138-5p, hsa-miR-144-3p, hsa-miR-195-5p, hsa-miR-199b-5p, hsa-miR-204-5p, hsa-miR-30a-3p, hsa-miR-451a, hsa-miR-99a-5p"

"x"

"1" "011100000, GSE151180, GSE113629, GSE103996, hsa-miR-144-3p, hsa-miR-144-5p, hsa-miR-204-5p, hsa-miR-451a, hsa-miR-486-5p, hsa-miR-7-2-3p, hsa-miR-7-5p, hsa-miR-718"

"x"

"1" "011001000, GSE151180, GSE113629, GSE63511, hsa-miR-144-3p, hsa-miR-144-5p, hsa-miR-204-5p, hsa-miR-363-3p, hsa-miR-451a, hsa-miR-486-5p, hsa-miR-7-2-3p, hsa-miR-7-5p"

"x"

"1" "011000001, GSE151180, GSE113629, GSE159330, hsa-miR-144-3p, hsa-miR-144-5p, hsa-miR-204-5p, hsa-miR-363-3p, hsa-miR-451a"

"x"

"1" "010110000, GSE151180, GSE103996, GSE73182, hsa-let-7g-5p, hsa-miR-100-5p, hsa-miR-126-3p, hsa-miR-130a-3p, hsa-miR-138-5p, hsa-miR-144-3p, hsa-miR-199b-5p, hsa-miR-204-5p, hsa-miR-218-5p, hsa-miR-26b-5p, hsa-miR-30a-3p, hsa-miR-30c-5p, hsa-miR-451a, hsa-miR-7-5p, hsa-miR-99a-5p"

"x"

"1" "010101000, GSE151180, GSE103996, GSE63511, hsa-miR-100-5p, hsa-miR-138-5p, hsa-miR-144-3p, hsa-miR-144-5p, hsa-miR-199b-5p, hsa-miR-204-5p, hsa-miR-30a-3p, hsa-miR-30a-5p, hsa-miR-451a, hsa-miR-486-5p, hsa-miR-7-2-3p, hsa-miR-7-5p, hsa-miR-99a-5p"

"x"

"1" "010100001, GSE151180, GSE103996, GSE159330, hsa-miR-100-5p, hsa-miR-126-3p, hsa-miR-130a-3p, hsa-miR-138-5p, hsa-miR-144-3p, hsa-miR-144-5p, hsa-miR-199b-5p, hsa-miR-204-5p, hsa-miR-30a-3p, hsa-miR-30a-5p, hsa-miR-451a, hsa-miR-99a-5p"

"x"

"1" "010011000, GSE151180, GSE73182, GSE63511, hsa-miR-100-5p, hsa-miR-138-5p, hsa-miR-144-3p, hsa-miR-199b-5p, hsa-miR-204-5p, hsa-miR-30a-3p, hsa-miR-451a, hsa-miR-7-5p, hsa-miR-99a-5p"

"x"

"1" "010010001, GSE151180, GSE73182, GSE159330, hsa-miR-100-5p, hsa-miR-126-3p, hsa-miR-130a-3p, hsa-miR-138-5p, hsa-miR-144-3p, hsa-miR-199b-5p, hsa-miR-204-5p, hsa-miR-30a-3p, hsa-miR-451a, hsa-miR-99a-5p"

"x"

"1" "010001010, GSE151180, GSE63511, GSE124653, hsa-miR-144-5p, hsa-miR-145-5p, hsa-miR-363-3p, hsa-miR-451a, hsa-miR-486-5p, hsa-miR-652-3p"

"x"

"1" "010001001, GSE151180, GSE63511, GSE159330, hsa-miR-100-5p, hsa-miR-138-5p, hsa-miR-144-3p, hsa-miR-144-5p, hsa-miR-145-5p, hsa-miR-152-3p, hsa-miR-199b-5p, hsa-miR-204-5p, hsa-miR-30a-3p, hsa-miR-30a-5p, hsa-miR-363-3p, hsa-miR-451a, hsa-miR-652-3p, hsa-miR-874-3p, hsa-miR-99a-5p"

"x"

"1" "010000011, GSE151180, GSE124653, GSE159330, hsa-miR-144-5p, hsa-miR-145-5p, hsa-miR-363-3p, hsa-miR-451a, hsa-miR-652-3p"

"x"

"1" "001101000, GSE113629, GSE103996, GSE63511, hsa-miR-144-3p, hsa-miR-144-5p, hsa-miR-204-5p, hsa-miR-451a, hsa-miR-486-5p, hsa-miR-7-2-3p, hsa-miR-7-5p"

"x"

"1" "001001001, GSE113629, GSE63511, GSE159330, hsa-miR-139-5p, hsa-miR-144-3p, hsa-miR-144-5p, hsa-miR-204-5p, hsa-miR-363-3p, hsa-miR-451a, hsa-miR-455-5p"

"x"

"1" "000111000, GSE103996, GSE73182, GSE63511, hsa-miR-100-5p, hsa-miR-101-3p, hsa-miR-138-5p, hsa-miR-144-3p, hsa-miR-195-5p, hsa-miR-199a-3p, hsa-miR-199b-5p, hsa-miR-204-5p, hsa-miR-214-3p, hsa-miR-30a-3p, hsa-miR-451a, hsa-miR-7-5p, hsa-miR-99a-5p"

"x"

"1" "000110001, GSE103996, GSE73182, GSE159330, hsa-miR-100-5p, hsa-miR-126-3p, hsa-miR-130a-3p, hsa-miR-138-5p, hsa-miR-144-3p, hsa-miR-195-5p, hsa-miR-199b-5p, hsa-miR-204-5p, hsa-miR-30a-3p, hsa-miR-451a, hsa-miR-99a-5p"

"x"

"1" "000101001, GSE103996, GSE63511, GSE159330, hsa-miR-100-5p, hsa-miR-138-5p, hsa-miR-144-3p, hsa-miR-144-5p, hsa-miR-195-5p, hsa-miR-199b-5p, hsa-miR-204-5p, hsa-miR-30a-3p, hsa-miR-30a-5p, hsa-miR-451a, hsa-miR-99a-5p"

"x"

"1" "000011001, GSE73182, GSE63511, GSE159330, hsa-miR-100-5p, hsa-miR-10b-5p, hsa-miR-138-5p, hsa-miR-144-3p, hsa-miR-195-5p, hsa-miR-199b-5p, hsa-miR-204-5p, hsa-miR-30a-3p, hsa-miR-451a, hsa-miR-497-5p, hsa-miR-99a-5p"

"x"

"1" "000001011, GSE63511, GSE124653, GSE159330, hsa-miR-144-5p, hsa-miR-145-5p, hsa-miR-16-2-3p, hsa-miR-363-3p, hsa-miR-451a, hsa-miR-652-3p"

"x"

"1" "011000000, GSE151180, GSE113629, hsa-miR-144-3p, hsa-miR-144-5p, hsa-miR-204-5p, hsa-miR-363-3p, hsa-miR-451a, hsa-miR-486-5p, hsa-miR-575, hsa-miR-7-2-3p, hsa-miR-7-5p, hsa-miR-718"

"x"

"1" "010100000, GSE151180, GSE103996, hsa-let-7g-5p, hsa-miR-100-5p, hsa-miR-126-3p, hsa-miR-130a-3p, hsa-miR-138-5p, hsa-miR-144-3p, hsa-miR-144-5p, hsa-miR-199b-5p, hsa-miR-204-5p, hsa-miR-218-5p, hsa-miR-26b-5p, hsa-miR-30a-3p, hsa-miR-30a-5p, hsa-miR-30c-5p, hsa-miR-451a, hsa-miR-4739, hsa-miR-486-5p, hsa-miR-7-2-3p, hsa-miR-7-5p, hsa-miR-718, hsa-miR-99a-5p"

"x"

"1" "010010000, GSE151180, GSE73182, hsa-let-7a-5p, hsa-let-7f-5p, hsa-let-7g-5p, hsa-miR-100-5p, hsa-miR-126-3p, hsa-miR-130a-3p, hsa-miR-138-5p, hsa-miR-144-3p, hsa-miR-199b-5p, hsa-miR-204-5p, hsa-miR-218-5p, hsa-miR-26b-5p, hsa-miR-30a-3p, hsa-miR-30c-5p, hsa-miR-365a-3p, hsa-miR-451a, hsa-miR-7-5p, hsa-miR-99a-5p"

"x"

"1" "010001000, GSE151180, GSE63511, hsa-miR-100-5p, hsa-miR-10a-5p, hsa-miR-138-5p, hsa-miR-144-3p, hsa-miR-144-5p, hsa-miR-145-5p, hsa-miR-152-3p, hsa-miR-199b-5p, hsa-miR-204-5p, hsa-miR-30a-3p, hsa-miR-30a-5p, hsa-miR-363-3p, hsa-miR-451a, hsa-miR-486-5p, hsa-miR-652-3p, hsa-miR-660-5p, hsa-miR-7-2-3p, hsa-miR-7-5p, hsa-miR-874-3p, hsa-miR-99a-5p"

"x"

"1" "010000010, GSE151180, GSE124653, hsa-miR-144-5p, hsa-miR-145-5p, hsa-miR-15b-5p, hsa-miR-193b-3p, hsa-miR-363-3p, hsa-miR-451a, hsa-miR-486-5p, hsa-miR-652-3p"

"x"

"1" "010000001, GSE151180, GSE159330, hsa-miR-100-5p, hsa-miR-126-3p, hsa-miR-126-5p, hsa-miR-130a-3p, hsa-miR-138-5p, hsa-miR-144-3p, hsa-miR-144-5p, hsa-miR-145-5p, hsa-miR-152-3p, hsa-miR-199b-5p, hsa-miR-204-5p, hsa-miR-30a-3p, hsa-miR-30a-5p, hsa-miR-363-3p, hsa-miR-451a, hsa-miR-652-3p, hsa-miR-874-3p, hsa-miR-99a-5p"

"x"

"1" "001100000, GSE113629, GSE103996, hsa-miR-144-3p, hsa-miR-144-5p, hsa-miR-204-5p, hsa-miR-3663-3p, hsa-miR-451a, hsa-miR-486-5p, hsa-miR-7-2-3p, hsa-miR-7-5p, hsa-miR-718"

"x"

"1" "001001000, GSE113629, GSE63511, hsa-miR-139-5p, hsa-miR-144-3p, hsa-miR-144-5p, hsa-miR-204-5p, hsa-miR-363-3p, hsa-miR-451a, hsa-miR-455-5p, hsa-miR-486-5p, hsa-miR-7-2-3p, hsa-miR-7-5p, hsa-miR-874-5p"

"x"

"1" "001000001, GSE113629, GSE159330, hsa-miR-139-5p, hsa-miR-144-3p, hsa-miR-144-5p, hsa-miR-204-5p, hsa-miR-363-3p, hsa-miR-451a, hsa-miR-455-5p"

"x"

"1" "000110000, GSE103996, GSE73182, hsa-let-7g-5p, hsa-miR-1, hsa-miR-100-5p, hsa-miR-101-3p, hsa-miR-126-3p, hsa-miR-130a-3p, hsa-miR-138-5p, hsa-miR-144-3p, hsa-miR-195-5p, hsa-miR-199a-3p, hsa-miR-199a-5p, hsa-miR-199b-5p, hsa-miR-204-5p, hsa-miR-214-3p, hsa-miR-218-5p, hsa-miR-26b-5p, hsa-miR-30a-3p, hsa-miR-30b-5p, hsa-miR-30c-5p, hsa-miR-451a, hsa-miR-7-5p, hsa-miR-99a-5p"

"x"

"1" "000101000, GSE103996, GSE63511, hsa-miR-100-5p, hsa-miR-101-3p, hsa-miR-138-5p, hsa-miR-143-3p, hsa-miR-144-3p, hsa-miR-144-5p, hsa-miR-195-5p, hsa-miR-199a-3p, hsa-miR-199b-5p, hsa-miR-204-5p, hsa-miR-214-3p, hsa-miR-30a-3p, hsa-miR-30a-5p, hsa-miR-451a, hsa-miR-486-5p, hsa-miR-7-2-3p, hsa-miR-7-5p, hsa-miR-99a-5p"

"x"

"1" "000100001, GSE103996, GSE159330, hsa-miR-100-5p, hsa-miR-126-3p, hsa-miR-130a-3p, hsa-miR-138-5p, hsa-miR-144-3p, hsa-miR-144-5p, hsa-miR-152, hsa-miR-192-5p, hsa-miR-195-5p, hsa-miR-199b-5p, hsa-miR-204-5p, hsa-miR-30a-3p, hsa-miR-30a-5p, hsa-miR-451a, hsa-miR-99a-5p"

"x"

"1" "000011000, GSE73182, GSE63511, hsa-miR-100-5p, hsa-miR-101-3p, hsa-miR-10b-5p, hsa-miR-138-5p, hsa-miR-144-3p, hsa-miR-195-5p, hsa-miR-199a-3p, hsa-miR-199b-5p, hsa-miR-204-5p, hsa-miR-214-3p, hsa-miR-30a-3p, hsa-miR-451a, hsa-miR-497-5p, hsa-miR-7-5p, hsa-miR-99a-5p"

"x"

"1" "000010001, GSE73182, GSE159330, hsa-miR-100-5p, hsa-miR-10b-5p, hsa-miR-126-3p, hsa-miR-130a-3p, hsa-miR-138-5p, hsa-miR-144-3p, hsa-miR-195-5p, hsa-miR-199b-5p, hsa-miR-204-5p, hsa-miR-30a-3p, hsa-miR-451a, hsa-miR-497-5p, hsa-miR-99a-5p"

"x"

"1" "000001010, GSE63511, GSE124653, hsa-miR-144-5p, hsa-miR-145-5p, hsa-miR-16-2-3p, hsa-miR-324-3p, hsa-miR-363-3p, hsa-miR-451a, hsa-miR-486-5p, hsa-miR-6087, hsa-miR-652-3p"

"x"

"1" "000001001, GSE63511, GSE159330, hsa-miR-100-5p, hsa-miR-106b-3p, hsa-miR-10b-5p, hsa-miR-138-5p, hsa-miR-139-5p, hsa-miR-144-3p, hsa-miR-144-5p, hsa-miR-145-5p, hsa-miR-152-3p, hsa-miR-16-2-3p, hsa-miR-195-5p, hsa-miR-199b-5p, hsa-miR-19a-3p, hsa-miR-204-5p, hsa-miR-30a-3p, hsa-miR-30a-5p, hsa-miR-363-3p, hsa-miR-451a, hsa-miR-455-5p, hsa-miR-497-5p, hsa-miR-652-3p, hsa-miR-708-5p, hsa-miR-874-3p, hsa-miR-99a-5p"

"x"

"1" "000000011, GSE124653, GSE159330, hsa-miR-144-5p, hsa-miR-145-5p, hsa-miR-16-2-3p, hsa-miR-363-3p, hsa-miR-451a, hsa-miR-652-3p"

"x"

"1" "100000000, GSE191117, hsa-miR-1180-3p, hsa-miR-135a-5p, hsa-miR-152-3p, hsa-miR-1910-5p, hsa-miR-200a-3p, hsa-miR-200b-3p, hsa-miR-200c-3p, hsa-miR-204-5p, hsa-miR-3151-5p, hsa-miR-7-5p"
