# Supplementary material for: Combination of hsa-miR-21-3p/ sTNF-RI/ IL12-p40 /CCL25 serves as a promising panel of diagnostic biomarkers for distinguishing malignant from benign nodules in papillary thyroid cancer
Source: Endocrine. 2026 Apr 27;91(1):146. doi: 10.1007/s12020-026-04612-9 (PMC13121238; doi:10.1007/s12020-026-04612-9)
Supplement: Supplementary file 3 — Supplementary Material 3 [file 12020_2026_4612_MOESM3_ESM.pdf]

Combination of hsa-miR-21-3p/ sTNF-RI/ IL12-p40 /CCL25 serves as a promising panel of diagnostic biomarkers for distinguishing malignant from benign nodules in papillary thyroid cancer.

Abdulmelik Aytatli1,2, Abdulkadir Sahin3, Neslisah Barlak1,2, Betul Gundogdu4, Arzu Tatar3, Omer Faruk KARATAS1,2,*

Supplementary File 2. Expression of microRNAs in normal tissues and organs that are commonly upregulated or downregulated, as identified by RNA-Seq and microarray analyses reported in the literature. This data was obtained from the miRGAtor database.

Downregulated in Organs

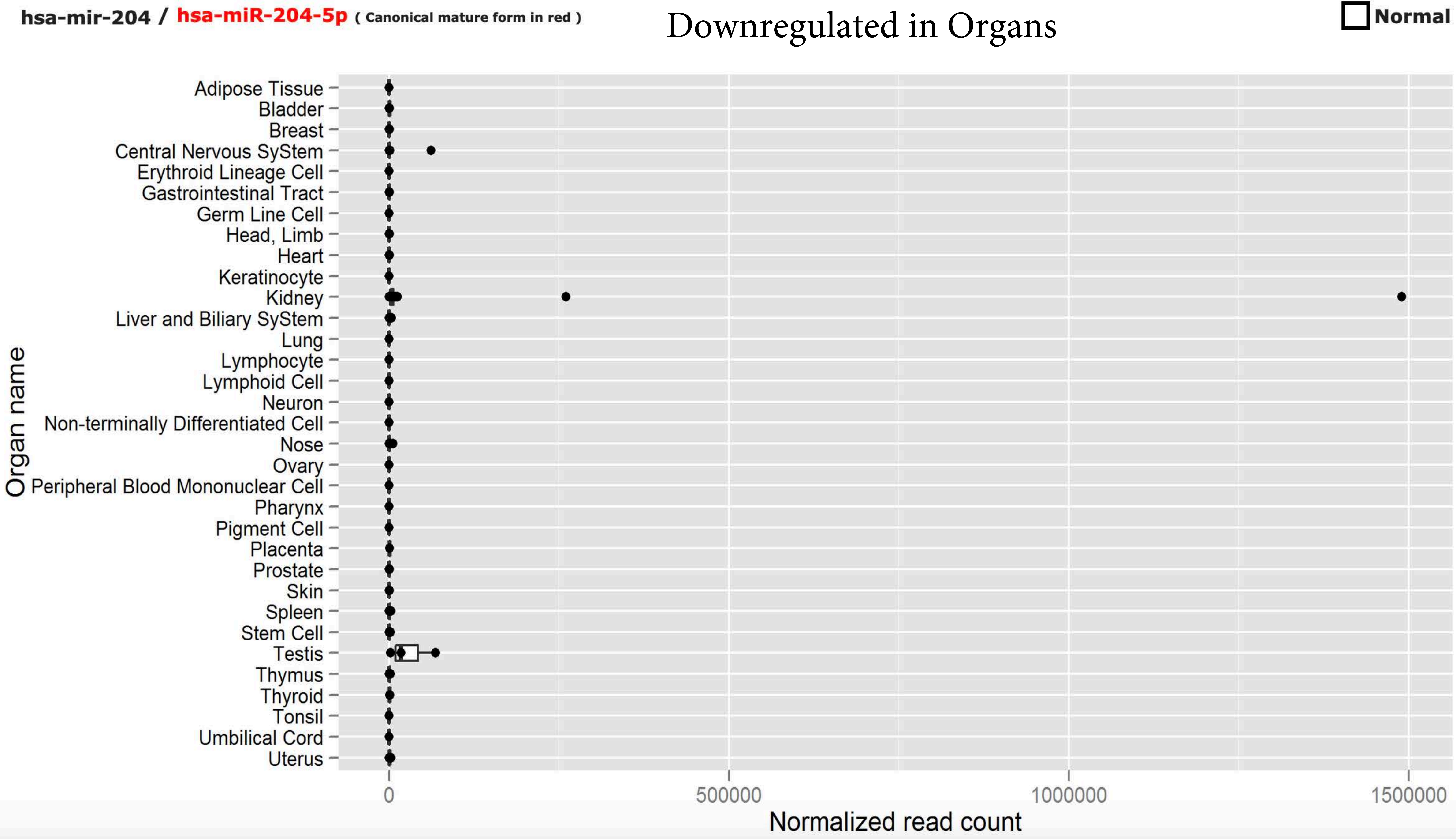

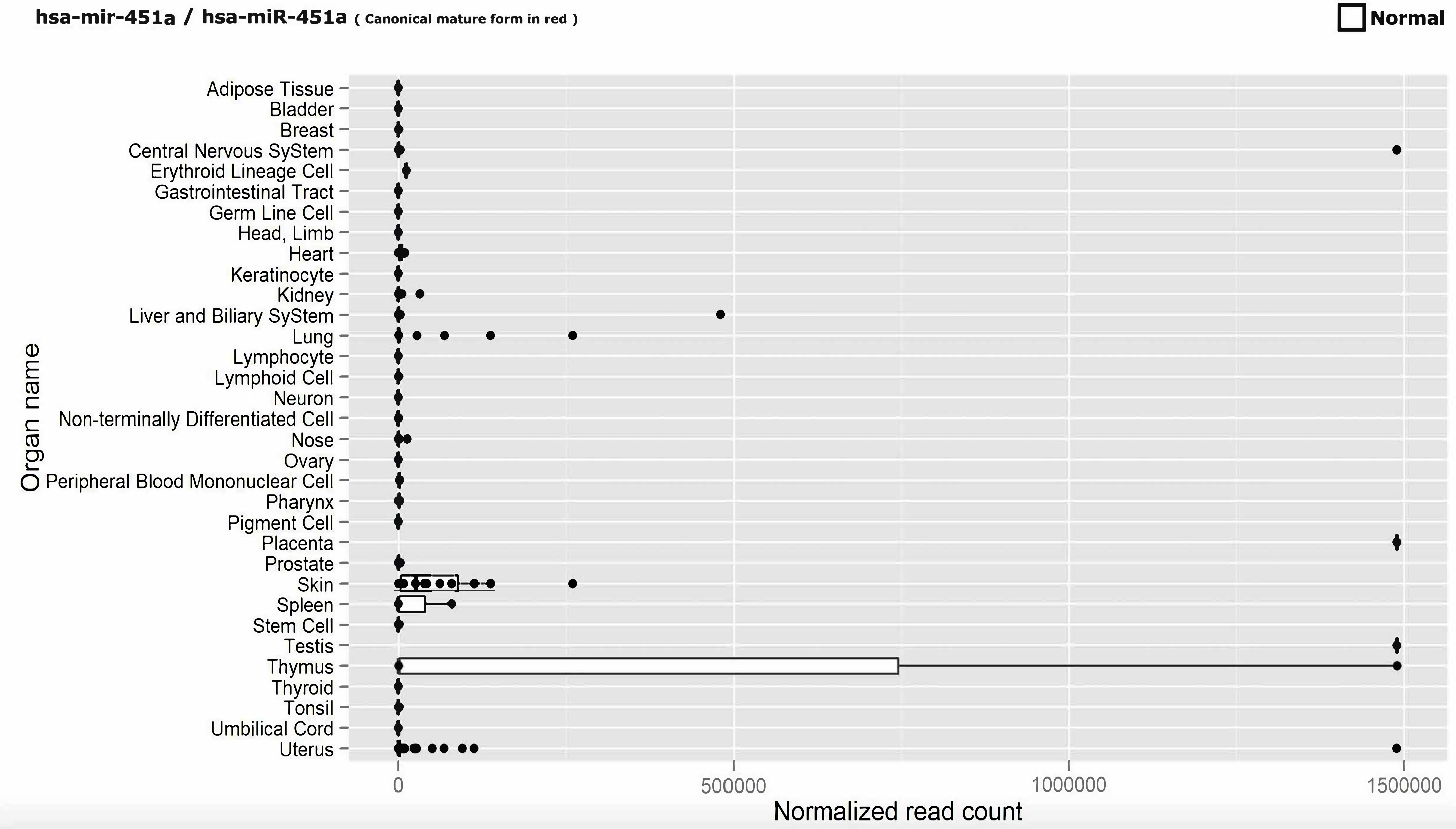

hsa-mir-451a / hsa-miR-451a (Canonical mature form in red)
Normal
Organ name
Adipose Tissue
Bladder
Breast
Central Nervous SyStem
Erythroid Lineage Cell
Gastrointestinal Tract
Germ Line Cell
Head, Limb
Heart
Keratinocyte
Kidney
Liver and Biliary SyStem
Lung
Lymphocyte
Lymphoid Cell
Neuron
Non-terminally Differentiated Cell
Nose
Ovary
Peripheral Blood Mononuclear Cell
Pharynx
Pigment Cell
Placenta
Prostate
Skin
Spleen
Stem Cell
Testis
Thymus
Thyroid
Tonsil
Umbilical Cord
Uterus
0
500000
1000000
1500000
Normalized read count

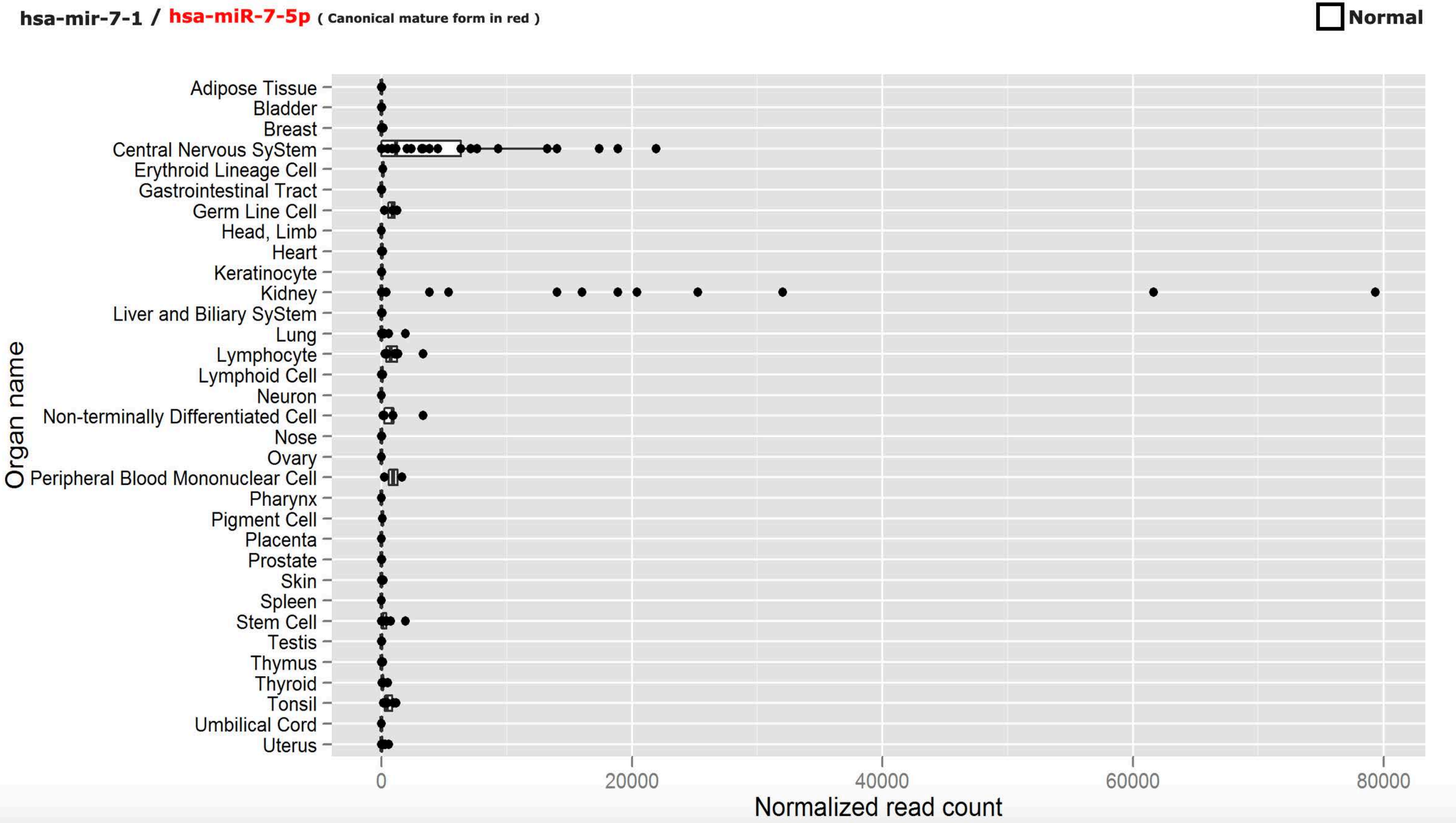
hsa-mir-7-1 / hsa-miR-7-5p (Canonical mature form in red)
Normal
Organ name
Adipose Tissue
Bladder
Breast
Central Nervous SyStem
Erythroid Lineage Cell
Gastrointestinal Tract
Germ Line Cell
Head, Limb
Heart
Keratinocyte
Kidney
Liver and Biliary SyStem
Lung
Lymphocyte
Lymphoid Cell
Neuron
Non-terminally Differentiated Cell
Nose
Ovary
Peripheral Blood Mononuclear Cell
Pharynx
Pigment Cell
Placenta
Prostate
Skin
Spleen
Stem Cell
Testis
Thymus
Thyroid
Tonsil
Umbilical Cord
Uterus
0
20000
40000
60000
80000
Normalized read count

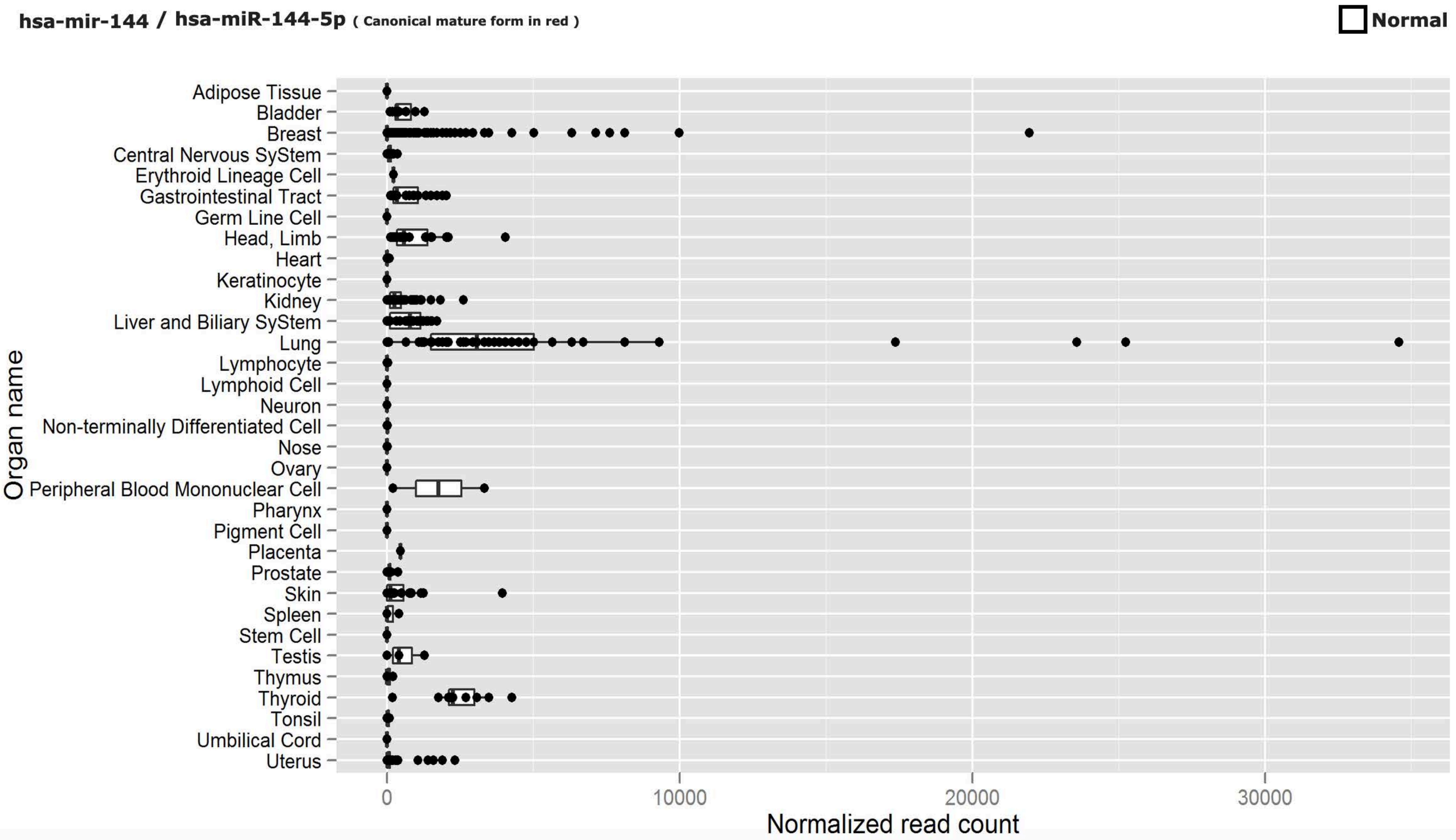
hsa-mir-144 / hsa-miR-144-5p (Canonical mature form in red)
Normal
Adipose Tissue
Bladder
Breast
Central Nervous SyStem
Erythroid Lineage Cell
Gastrointestinal Tract
Germ Line Cell
Head, Limb
Heart
Keratinocyte
Kidney
Liver and Biliary SyStem
Lung
Lymphocyte
Lymphoid Cell
Neuron
Non-terminally Differentiated Cell
Nose
Ovary
Peripheral Blood Mononuclear Cell
Pharynx
Pigment Cell
Placenta
Prostate
Skin
Spleen
Stem Cell
Testis
Thymus
Thyroid
Tonsil
Umbilical Cord
Uterus
Organ name
0
10000
20000
30000
Normalized read count

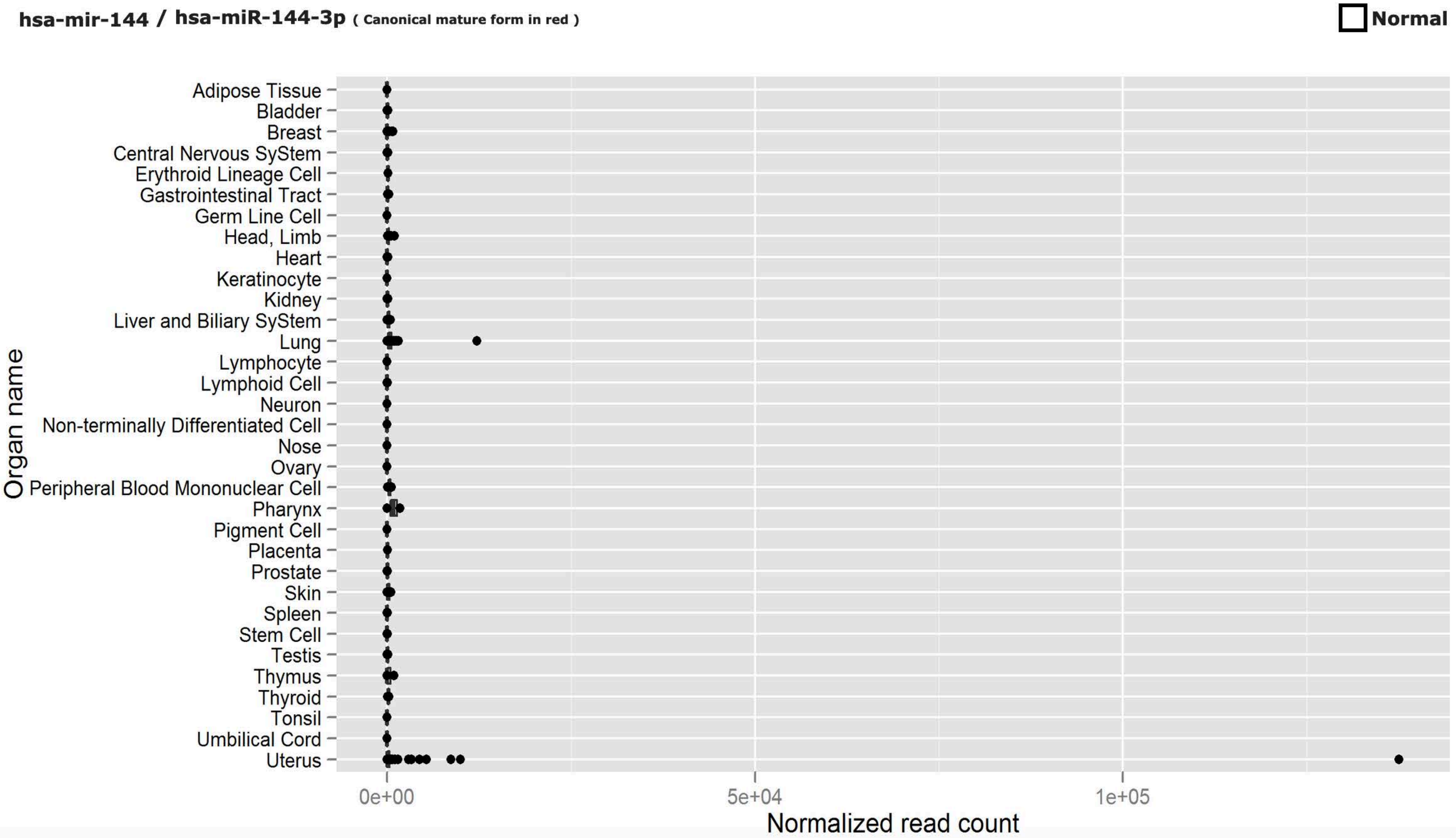
hsa-mir-144 / hsa-miR-144-3p (Canonical mature form in red)
Normal
Organ name
Adipose Tissue
Bladder
Breast
Central Nervous SyStem
Erythroid Lineage Cell
Gastrointestinal Tract
Germ Line Cell
Head, Limb
Heart
Keratinocyte
Kidney
Liver and Biliary SyStem
Lung
Lymphocyte
Lymphoid Cell
Neuron
Non-terminally Differentiated Cell
Nose
Ovary
Peripheral Blood Mononuclear Cell
Pharynx
Pigment Cell
Placenta
Prostate
Skin
Spleen
Stem Cell
Testis
Thymus
Thyroid
Tonsil
Umbilical Cord
Uterus
0e+00
5e+04
1e+05
Normalized read count

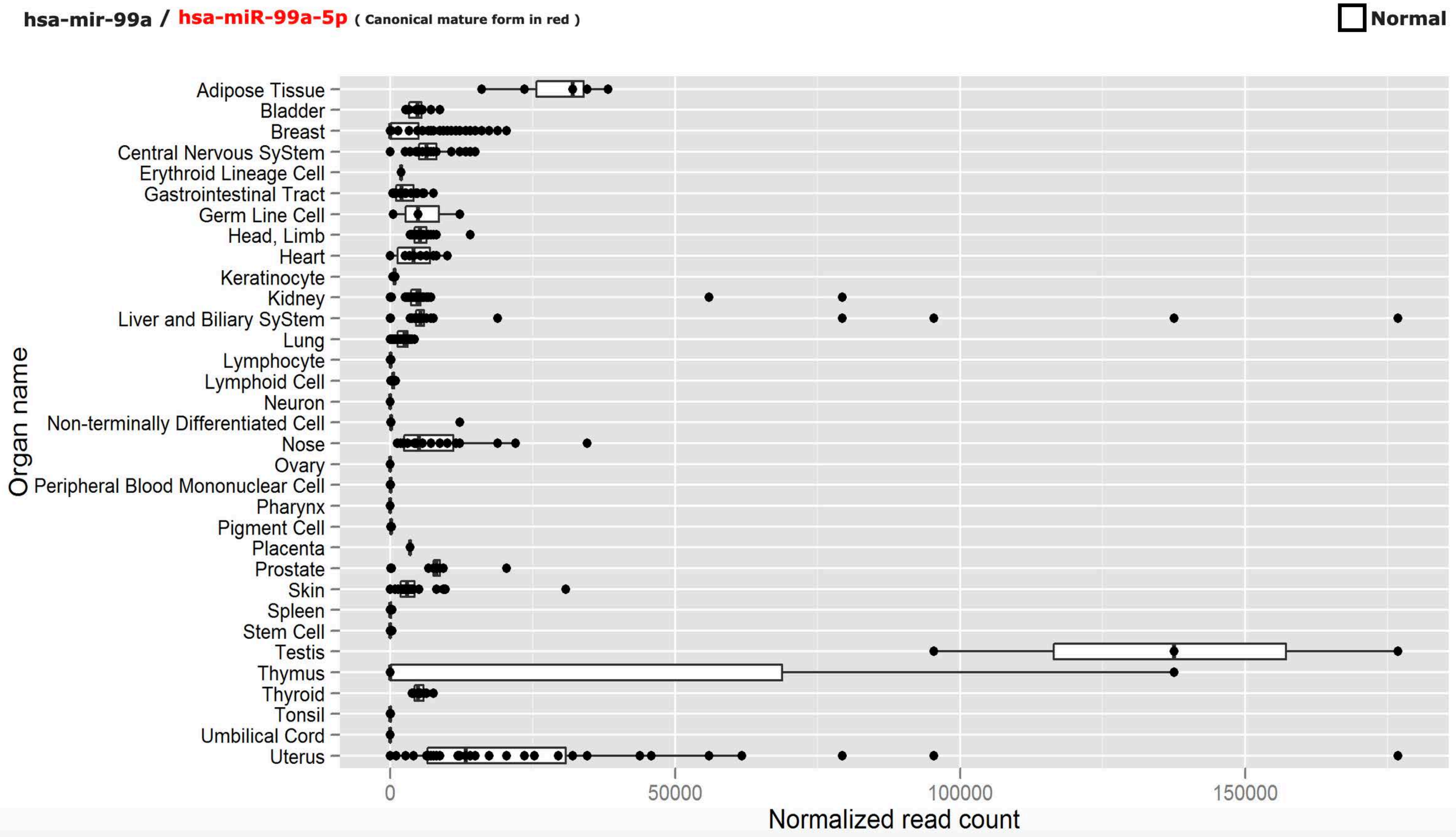

hsa-mir-99a / hsa-miR-99a-5p (Canonical mature form in red)
Normal
Adipose Tissue
Bladder
Breast
Central Nervous SyStem
Erythroid Lineage Cell
Gastrointestinal Tract
Germ Line Cell
Head, Limb
Heart
Keratinocyte
Kidney
Liver and Biliary SyStem
Lung
Lymphocyte
Lymphoid Cell
Neuron
Non-terminally Differentiated Cell
Nose
Ovary
Peripheral Blood Mononuclear Cell
Pharynx
Pigment Cell
Placenta
Prostate
Skin
Spleen
Stem Cell
Testis
Thymus
Thyroid
Tonsil
Umbilical Cord
Uterus
Organ name
0
50000
100000
150000
Normalized read count

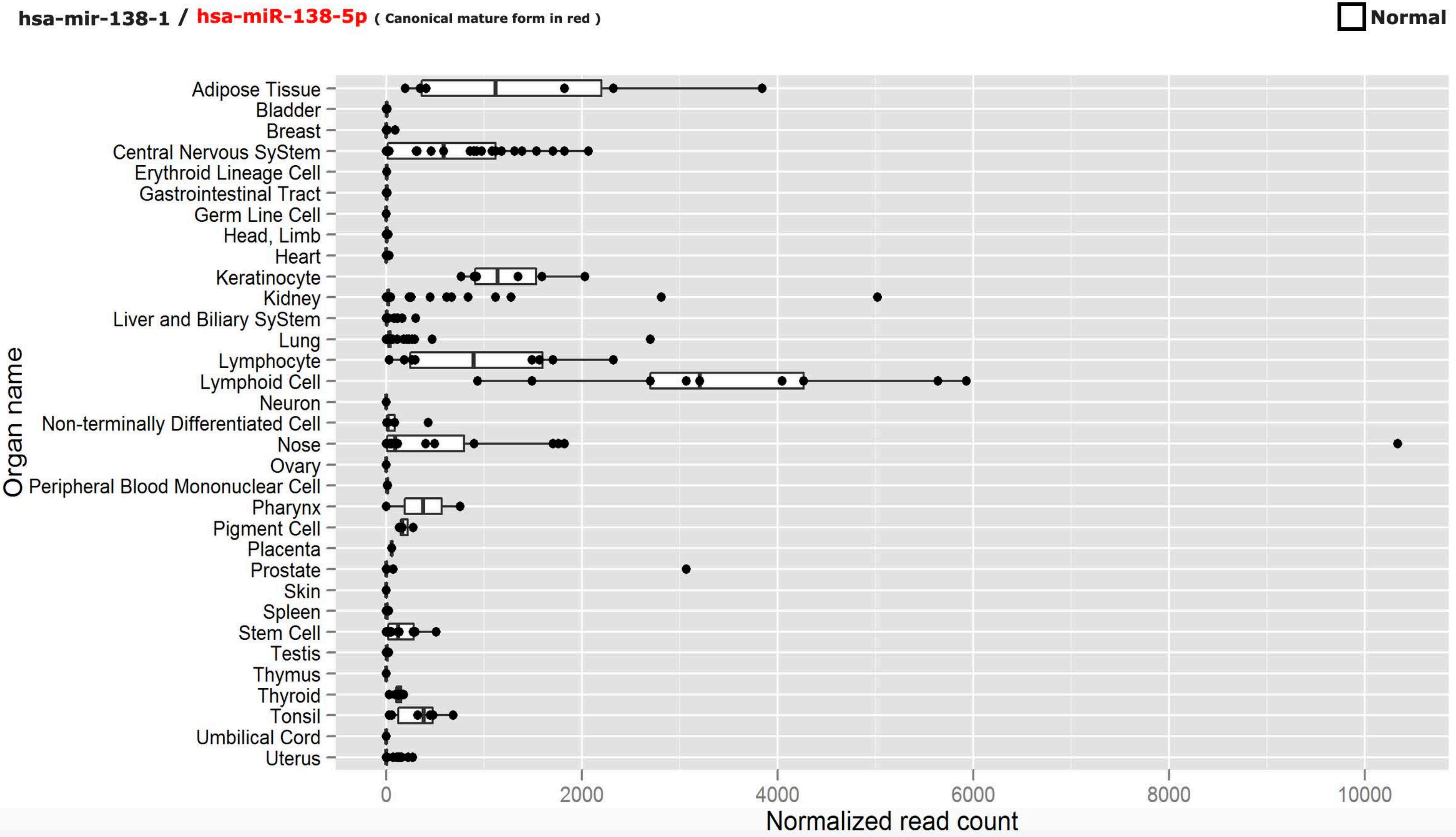
hsa-mir-138-1 / hsa-miR-138-5p (Canonical mature form in red)
Normal
Organ name
Adipose Tissue
Bladder
Breast
Central Nervous SyStem
Erythroid Lineage Cell
Gastrointestinal Tract
Germ Line Cell
Head, Limb
Heart
Keratinocyte
Kidney
Liver and Biliary SyStem
Lung
Lymphocyte
Lymphoid Cell
Neuron
Non-terminally Differentiated Cell
Nose
Ovary
Peripheral Blood Mononuclear Cell
Pharynx
Pigment Cell
Placenta
Prostate
Skin
Spleen
Stem Cell
Testis
Thymus
Thyroid
Tonsil
Umbilical Cord
Uterus
0
2000
4000
6000
8000
10000
Normalized read count

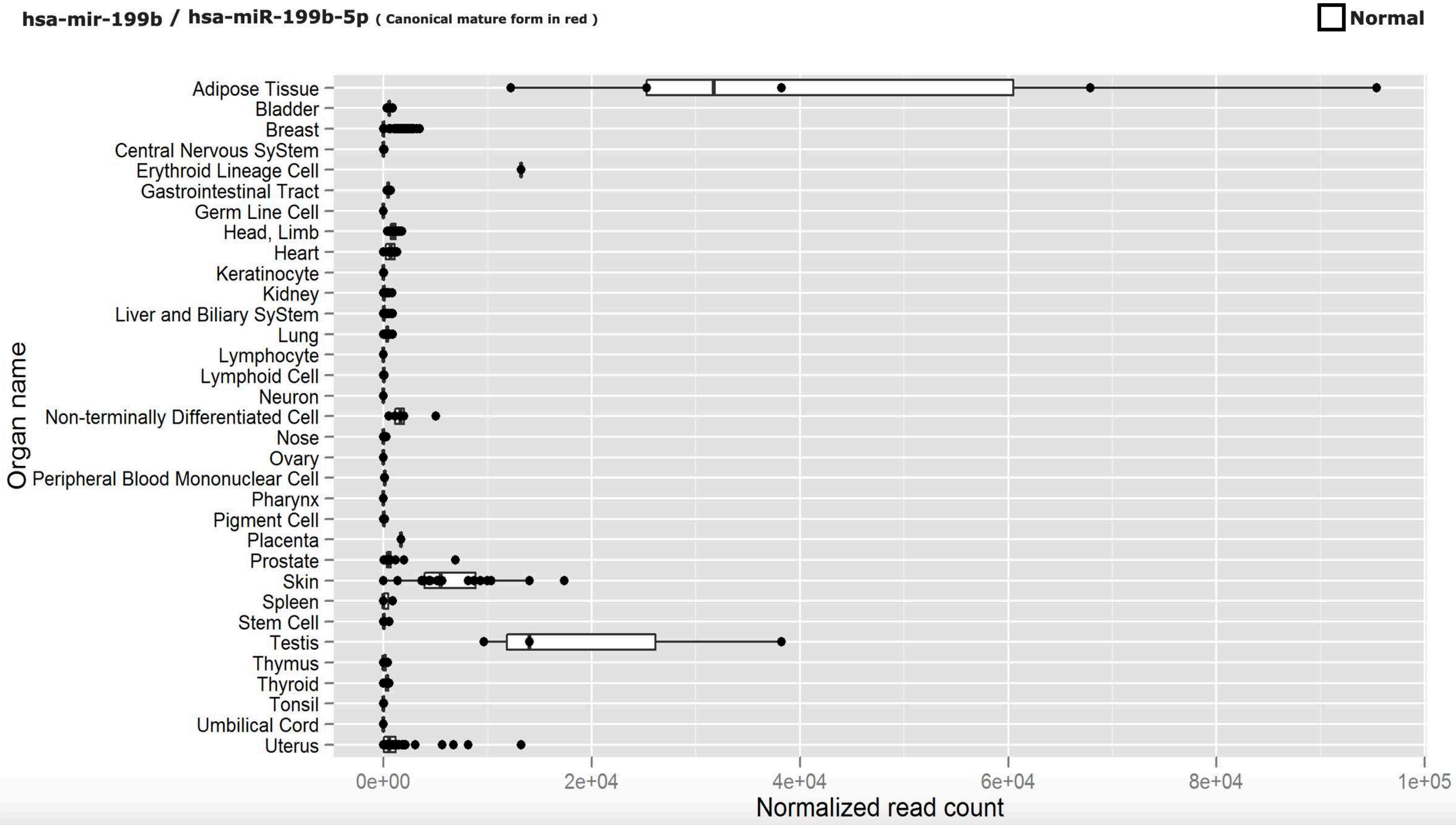
hsa-mir-199b / hsa-miR-199b-5p (Canonical mature form in red)
Normal
Organ name
Adipose Tissue
Bladder
Breast
Central Nervous SyStem
Erythroid Lineage Cell
Gastrointestinal Tract
Germ Line Cell
Head, Limb
Heart
Keratinocyte
Kidney
Liver and Biliary SyStem
Lung
Lymphocyte
Lymphoid Cell
Neuron
Non-terminally Differentiated Cell
Nose
Ovary
Peripheral Blood Mononuclear Cell
Pharynx
Pigment Cell
Placenta
Prostate
Skin
Spleen
Stem Cell
Testis
Thymus
Thyroid
Tonsil
Umbilical Cord
Uterus
0e+00
2e+04
4e+04
6e+04
8e+04
1e+05
Normalized read count

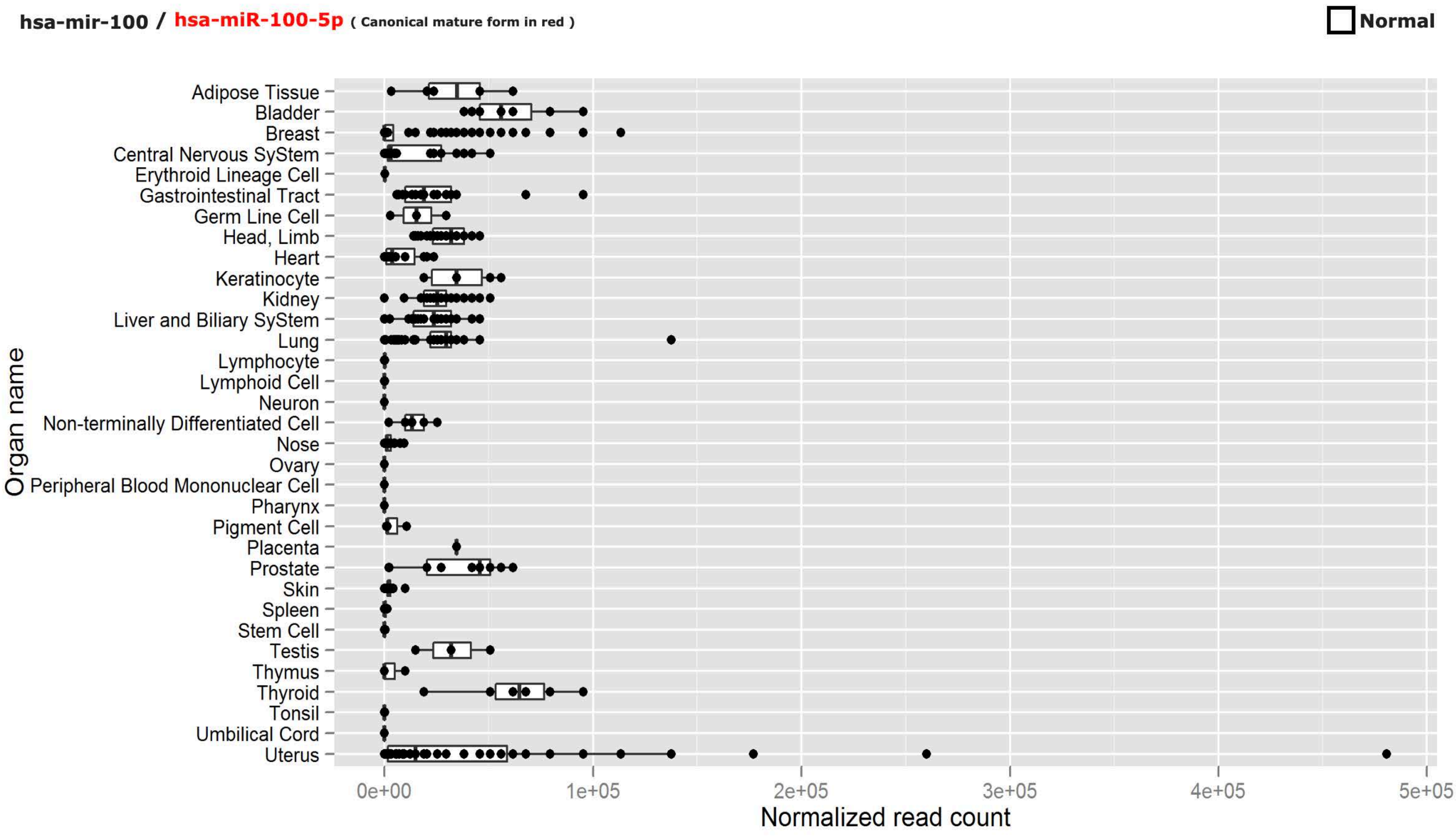
hsa-mir-100 / hsa-miR-100-5p (Canonical mature form in red)
Normal
Organ name
Adipose Tissue
Bladder
Breast
Central Nervous SyStem
Erythroid Lineage Cell
Gastrointestinal Tract
Germ Line Cell
Head, Limb
Heart
Keratinocyte
Kidney
Liver and Biliary SyStem
Lung
Lymphocyte
Lymphoid Cell
Neuron
Non-terminally Differentiated Cell
Nose
Ovary
Peripheral Blood Mononuclear Cell
Pharynx
Pigment Cell
Placenta
Prostate
Skin
Spleen
Stem Cell
Testis
Thymus
Thyroid
Tonsil
Umbilical Cord
Uterus
0e+00
1e+05
2e+05
3e+05
4e+05
5e+05
Normalized read count

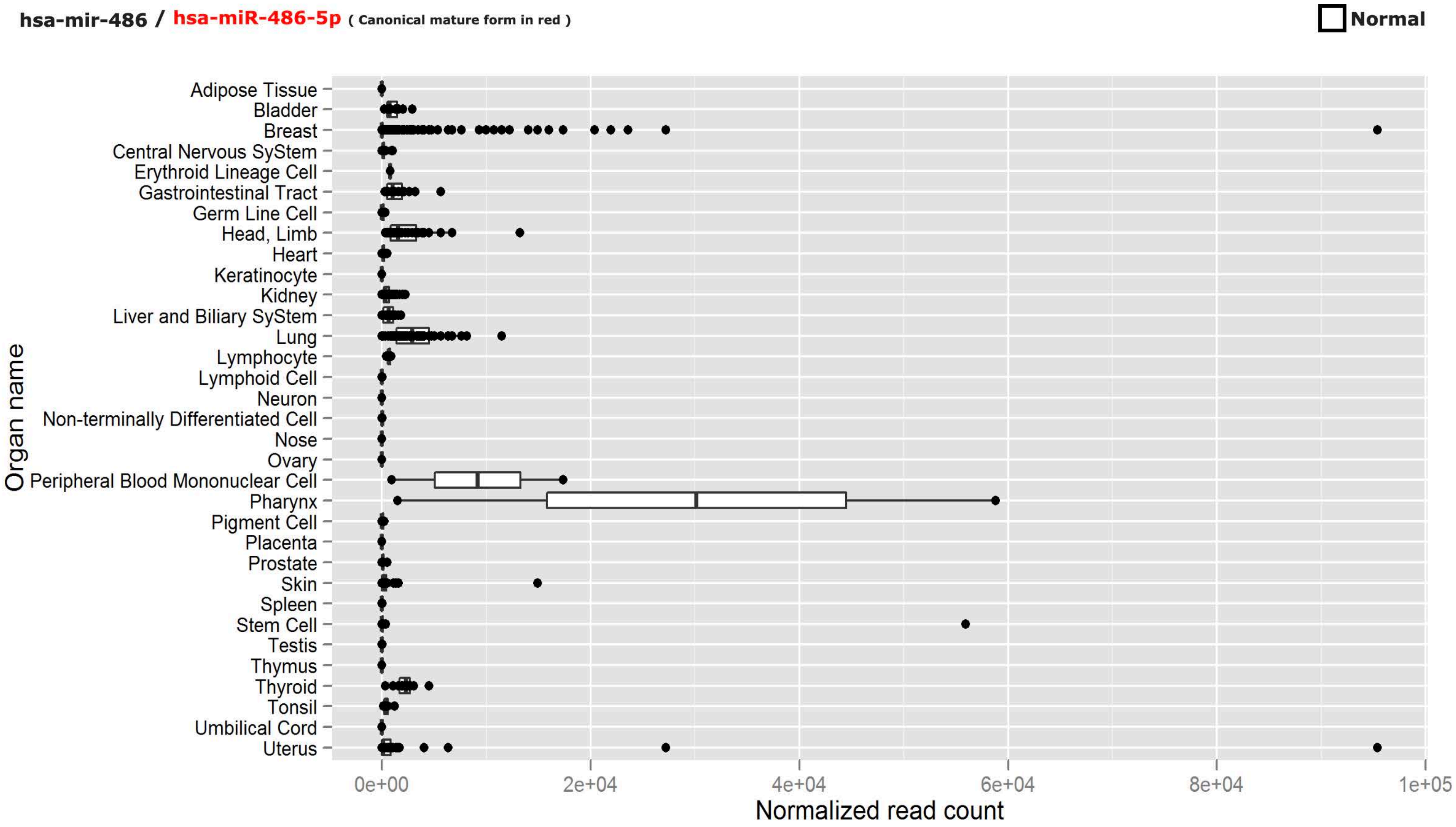
hsa-mir-486 / hsa-miR-486-5p (Canonical mature form in red)
Normal
Organ name
Adipose Tissue
Bladder
Breast
Central Nervous SyStem
Erythroid Lineage Cell
Gastrointestinal Tract
Germ Line Cell
Head, Limb
Heart
Keratinocyte
Kidney
Liver and Biliary SyStem
Lung
Lymphocyte
Lymphoid Cell
Neuron
Non-terminally Differentiated Cell
Nose
Ovary
Peripheral Blood Mononuclear Cell
Pharynx
Pigment Cell
Placenta
Prostate
Skin
Spleen
Stem Cell
Testis
Thymus
Thyroid
Tonsil
Umbilical Cord
Uterus
0e+00
2e+04
4e+04
6e+04
8e+04
1e+05
Normalized read count

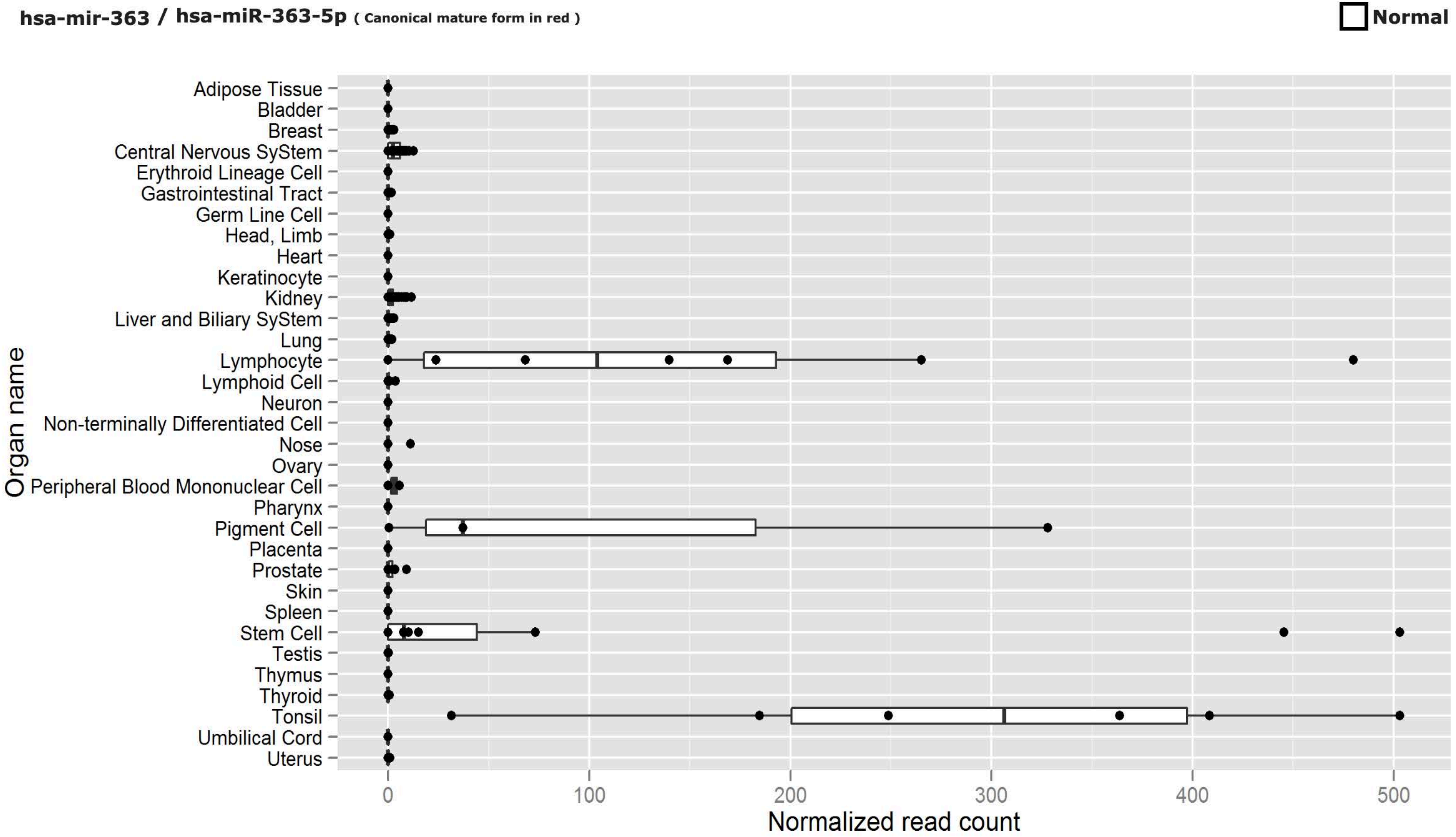
hsa-mir-363 / hsa-miR-363-5p (Canonical mature form in red)
Normal
Organ name
Adipose Tissue
Bladder
Breast
Central Nervous SyStem
Erythroid Lineage Cell
Gastrointestinal Tract
Germ Line Cell
Head, Limb
Heart
Keratinocyte
Kidney
Liver and Biliary SyStem
Lung
Lymphocyte
Lymphoid Cell
Neuron
Non-terminally Differentiated Cell
Nose
Ovary
Peripheral Blood Mononuclear Cell
Pharynx
Pigment Cell
Placenta
Prostate
Skin
Spleen
Stem Cell
Testis
Thymus
Thyroid
Tonsil
Umbilical Cord
Uterus
0
100
200
300
400
500
Normalized read count

Upregulated in Organs

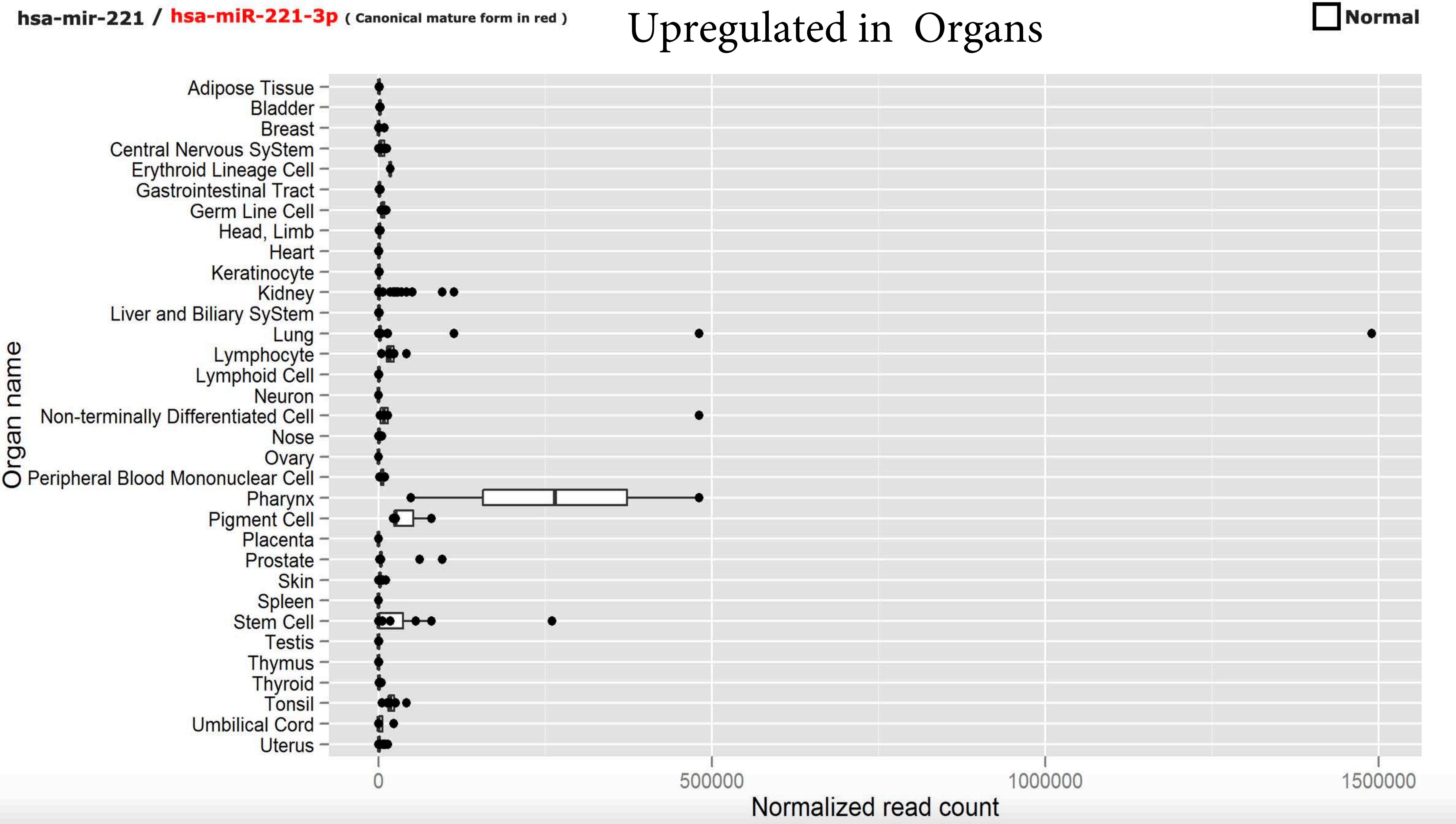

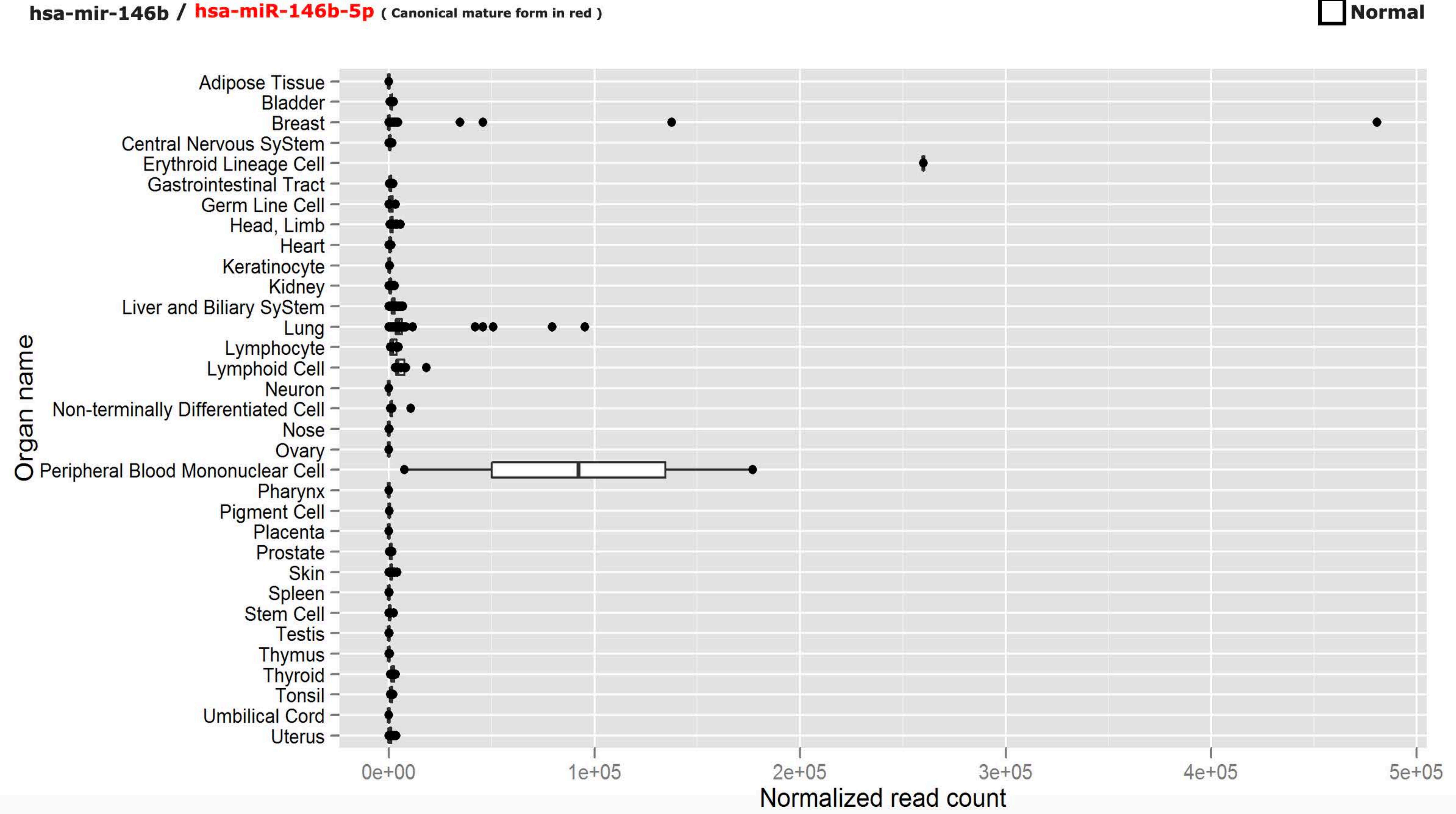
hsa-mir-146b / hsa-miR-146b-5p (Canonical mature form in red)
Normal
Adipose Tissue
Bladder
Breast
Central Nervous SyStem
Erythroid Lineage Cell
Gastrointestinal Tract
Germ Line Cell
Head, Limb
Heart
Keratinocyte
Kidney
Liver and Biliary SyStem
Lung
Lymphocyte
Lymphoid Cell
Neuron
Non-terminally Differentiated Cell
Nose
Ovary
Peripheral Blood Mononuclear Cell
Pharynx
Pigment Cell
Placenta
Prostate
Skin
Spleen
Stem Cell
Testis
Thymus
Thyroid
Tonsil
Umbilical Cord
Uterus
Organ name
0e+00
1e+05
2e+05
3e+05
4e+05
5e+05
Normalized read count

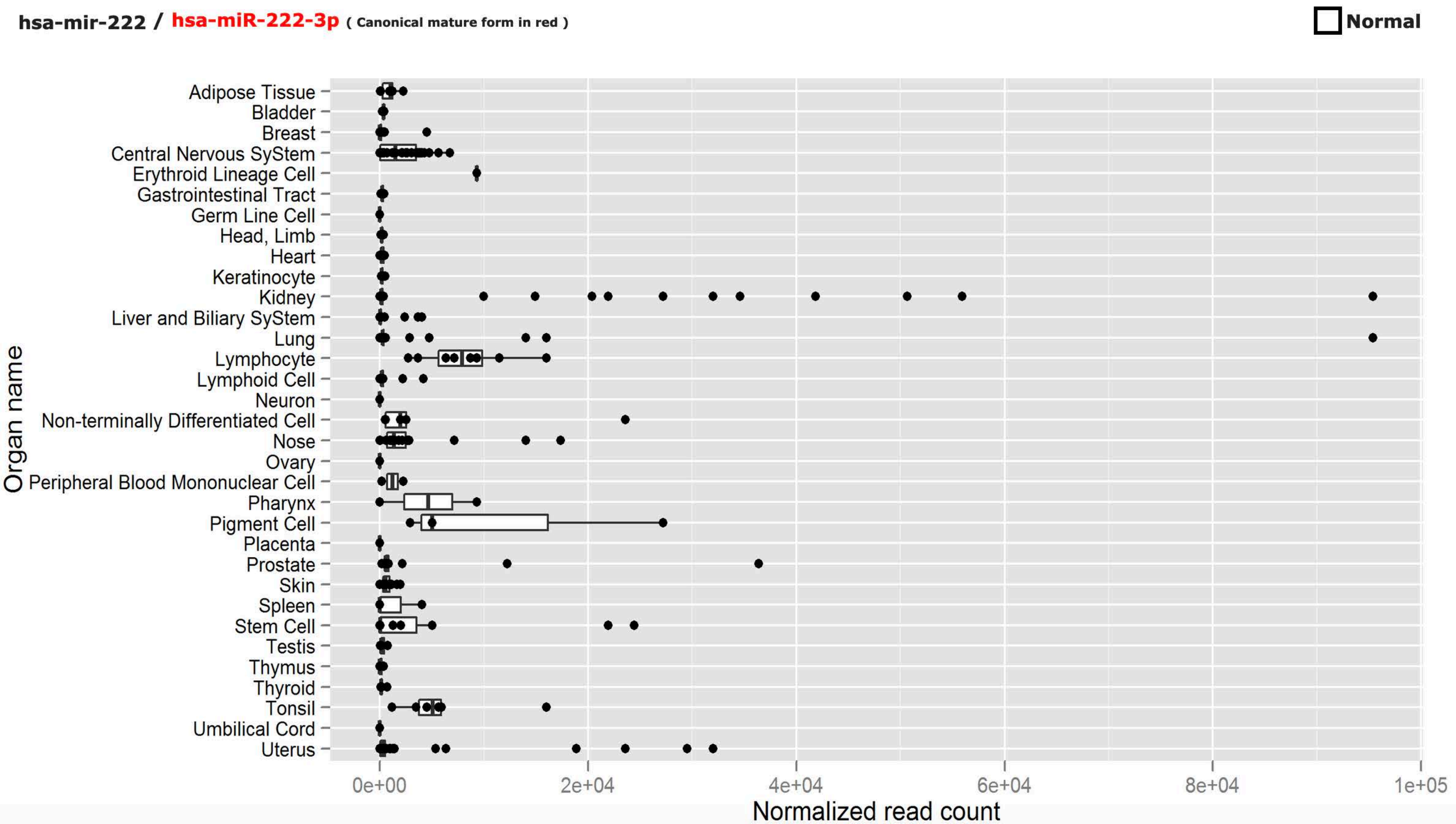

hsa-mir-222 / hsa-miR-222-3p (Canonical mature form in red)
Normal
Adipose Tissue
Bladder
Breast
Central Nervous SyStem
Erythroid Lineage Cell
Gastrointestinal Tract
Germ Line Cell
Head, Limb
Heart
Keratinocyte
Kidney
Liver and Biliary SyStem
Lung
Lymphocyte
Lymphoid Cell
Neuron
Non-terminally Differentiated Cell
Nose
Ovary
Peripheral Blood Mononuclear Cell
Pharynx
Pigment Cell
Placenta
Prostate
Skin
Spleen
Stem Cell
Testis
Thymus
Thyroid
Tonsil
Umbilical Cord
Uterus
Organ name
0e+00
2e+04
4e+04
6e+04
8e+04
1e+05
Normalized read count

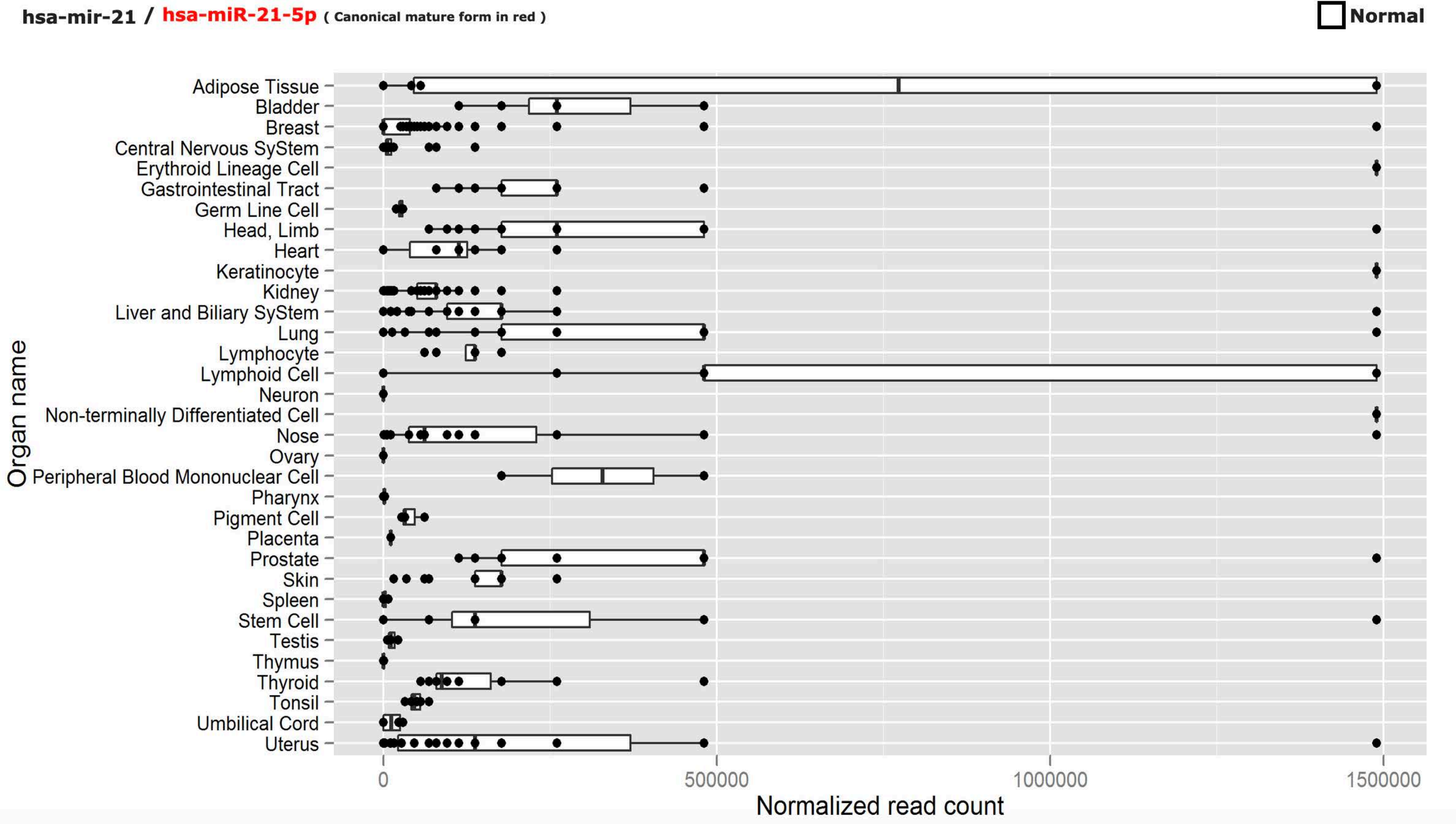

hsa-mir-21 / hsa-miR-21-5p (Canonical mature form in red)
Normal
Organ name
Adipose Tissue
Bladder
Breast
Central Nervous SyStem
Erythroid Lineage Cell
Gastrointestinal Tract
Germ Line Cell
Head, Limb
Heart
Keratinocyte
Kidney
Liver and Biliary SyStem
Lung
Lymphocyte
Lymphoid Cell
Neuron
Non-terminally Differentiated Cell
Nose
Ovary
Peripheral Blood Mononuclear Cell
Pharynx
Pigment Cell
Placenta
Prostate
Skin
Spleen
Stem Cell
Testis
Thymus
Thyroid
Tonsil
Umbilical Cord
Uterus
0
500000
1000000
1500000
Normalized read count

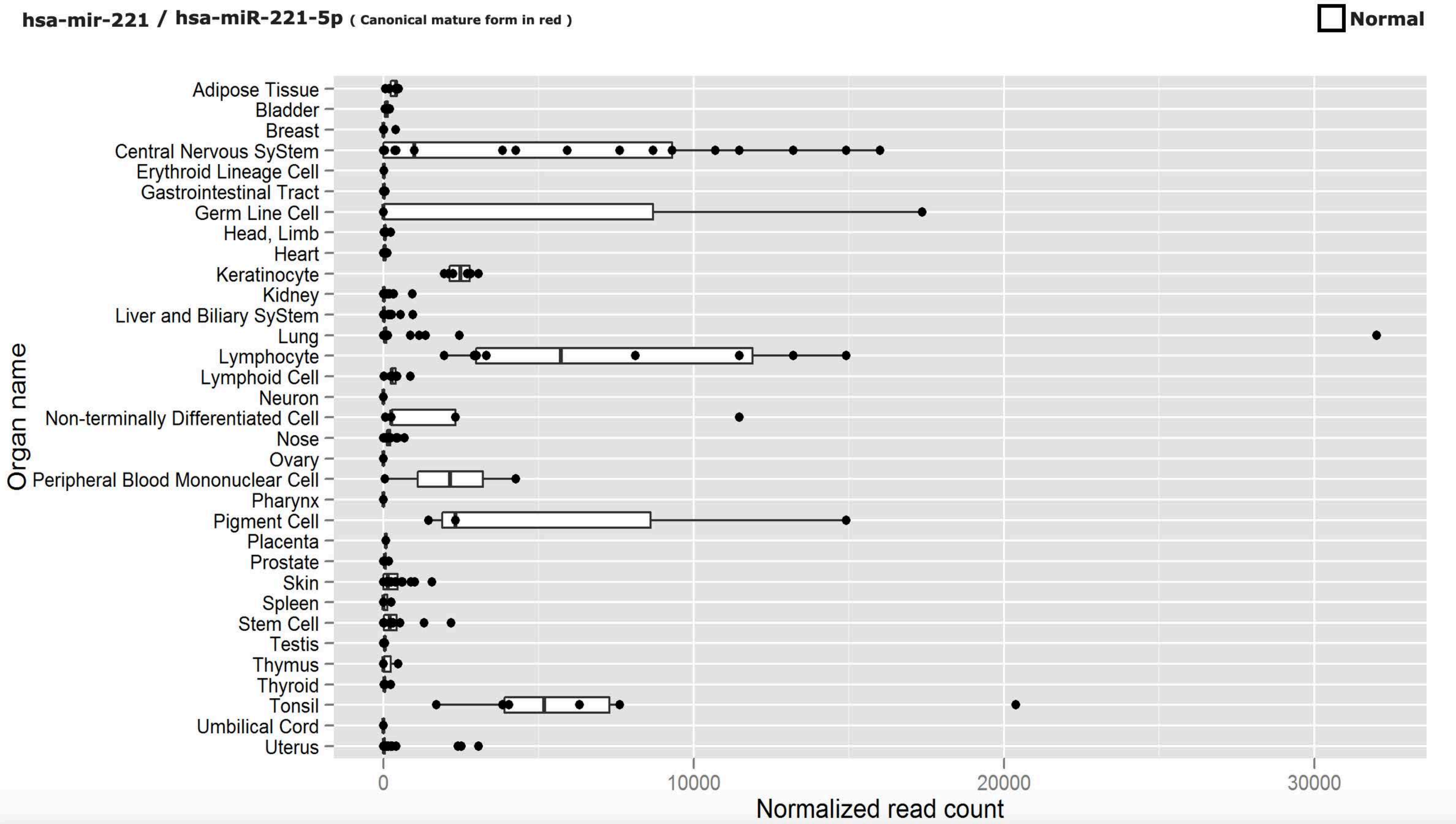
hsa-mir-221 / hsa-miR-221-5p (Canonical mature form in red)
Normal
Organ name
Adipose Tissue
Bladder
Breast
Central Nervous SyStem
Erythroid Lineage Cell
Gastrointestinal Tract
Germ Line Cell
Head, Limb
Heart
Keratinocyte
Kidney
Liver and Biliary SyStem
Lung
Lymphocyte
Lymphoid Cell
Neuron
Non-terminally Differentiated Cell
Nose
Ovary
Peripheral Blood Mononuclear Cell
Pharynx
Pigment Cell
Placenta
Prostate
Skin
Spleen
Stem Cell
Testis
Thymus
Thyroid
Tonsil
Umbilical Cord
Uterus
0
10000
20000
30000
Normalized read count

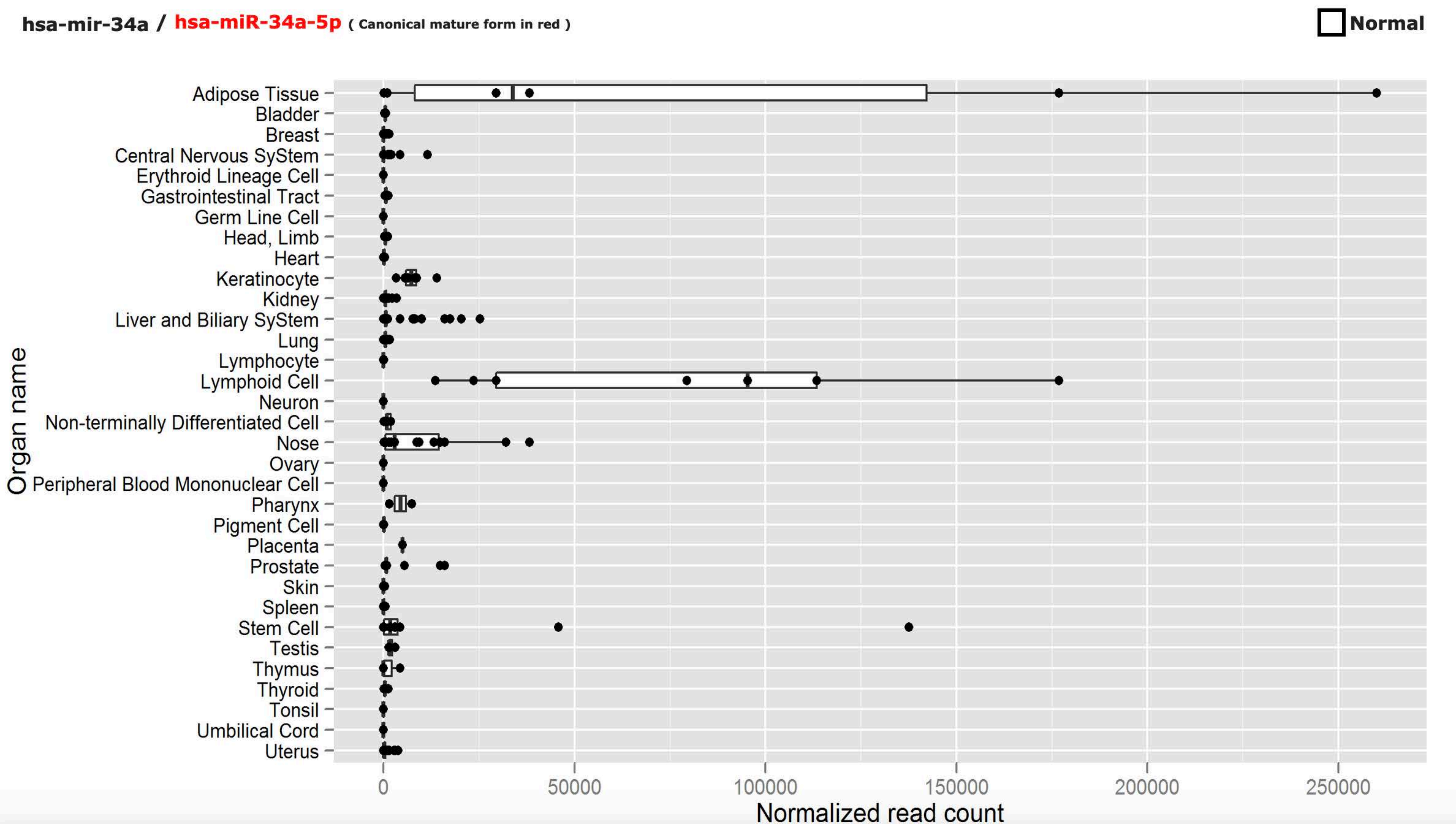
hsa-mir-34a / hsa-miR-34a-5p (Canonical mature form in red)
Normal
Organ name
Adipose Tissue
Bladder
Breast
Central Nervous SyStem
Erythroid Lineage Cell
Gastrointestinal Tract
Germ Line Cell
Head, Limb
Heart
Keratinocyte
Kidney
Liver and Biliary SyStem
Lung
Lymphocyte
Lymphoid Cell
Neuron
Non-terminally Differentiated Cell
Nose
Ovary
Peripheral Blood Mononuclear Cell
Pharynx
Pigment Cell
Placenta
Prostate
Skin
Spleen
Stem Cell
Testis
Thymus
Thyroid
Tonsil
Umbilical Cord
Uterus
0
50000
100000
150000
200000
250000
Normalized read count

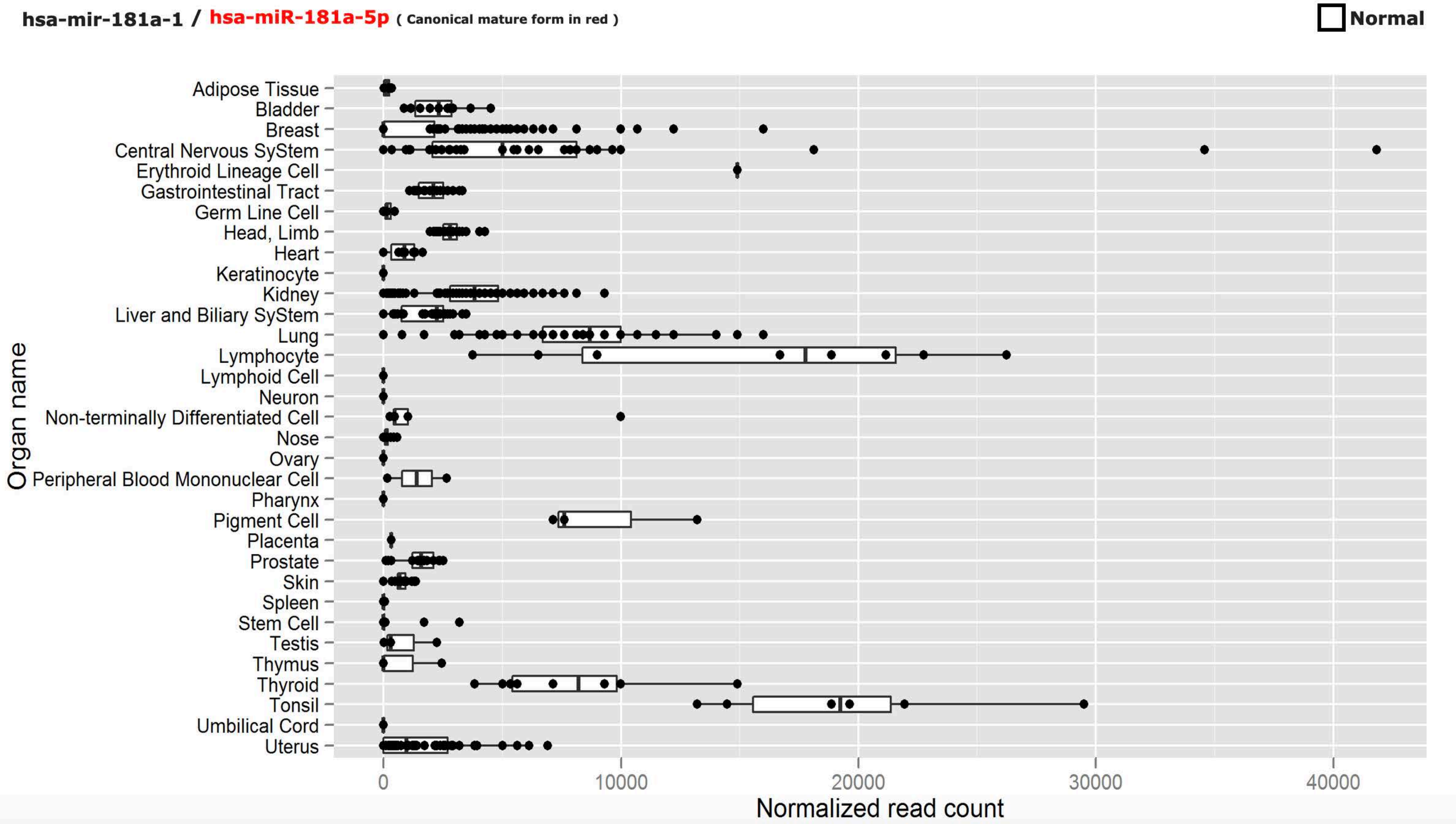

hsa-mir-181a-1 / hsa-miR-181a-5p (Canonical mature form in red)
Normal
Organ name
Adipose Tissue
Bladder
Breast
Central Nervous SyStem
Erythroid Lineage Cell
Gastrointestinal Tract
Germ Line Cell
Head, Limb
Heart
Keratinocyte
Kidney
Liver and Biliary SyStem
Lung
Lymphocyte
Lymphoid Cell
Neuron
Non-terminally Differentiated Cell
Nose
Ovary
Peripheral Blood Mononuclear Cell
Pharynx
Pigment Cell
Placenta
Prostate
Skin
Spleen
Stem Cell
Testis
Thymus
Thyroid
Tonsil
Umbilical Cord
Uterus
0
10000
20000
30000
40000
Normalized read count

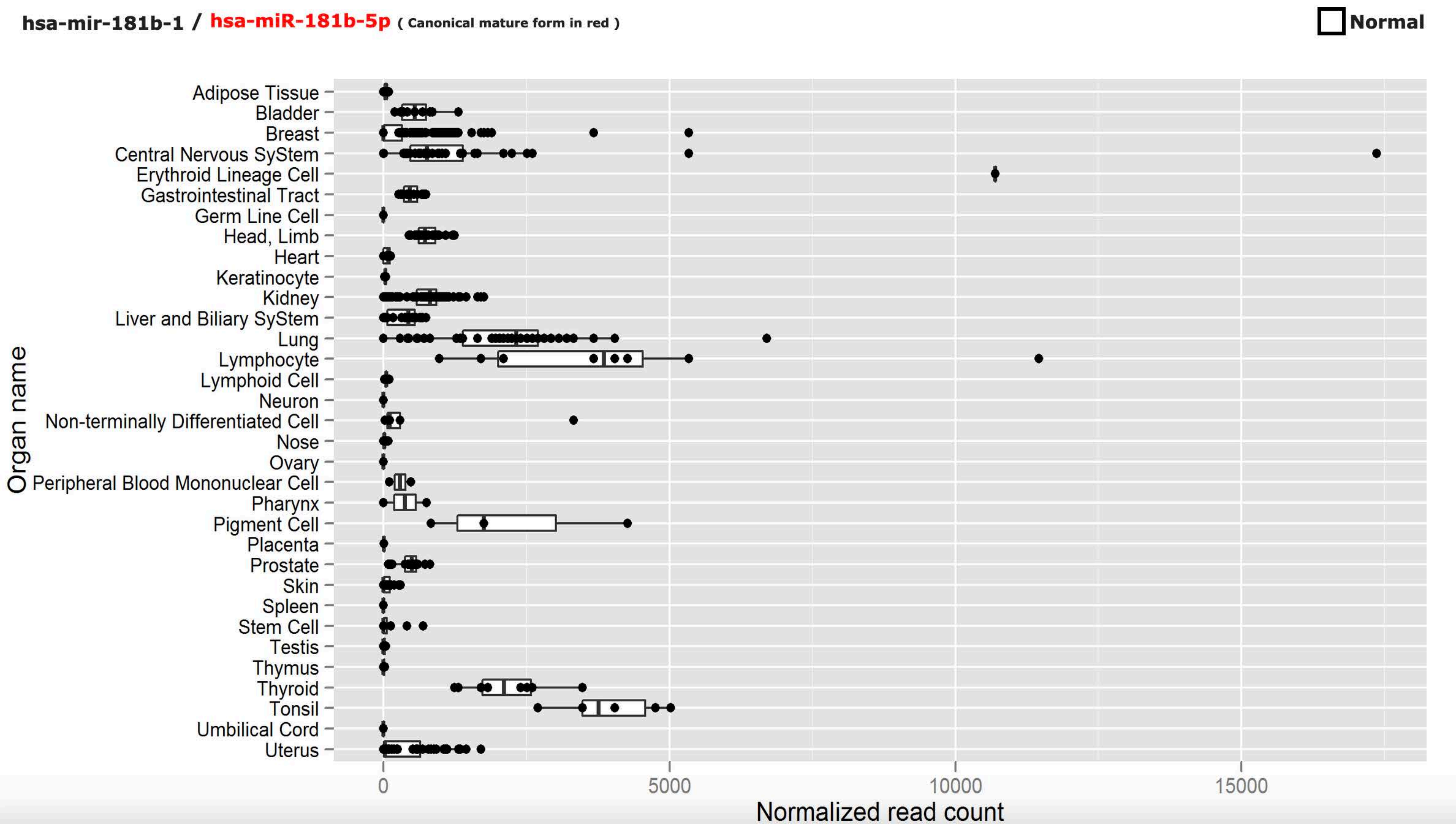
hsa-mir-181b-1 / hsa-miR-181b-5p (Canonical mature form in red)
Normal
Organ name
Adipose Tissue
Bladder
Breast
Central Nervous SyStem
Erythroid Lineage Cell
Gastrointestinal Tract
Germ Line Cell
Head, Limb
Heart
Keratinocyte
Kidney
Liver and Biliary SyStem
Lung
Lymphocyte
Lymphoid Cell
Neuron
Non-terminally Differentiated Cell
Nose
Ovary
Peripheral Blood Mononuclear Cell
Pharynx
Pigment Cell
Placenta
Prostate
Skin
Spleen
Stem Cell
Testis
Thymus
Thyroid
Tonsil
Umbilical Cord
Uterus
0
5000
10000
15000
Normalized read count

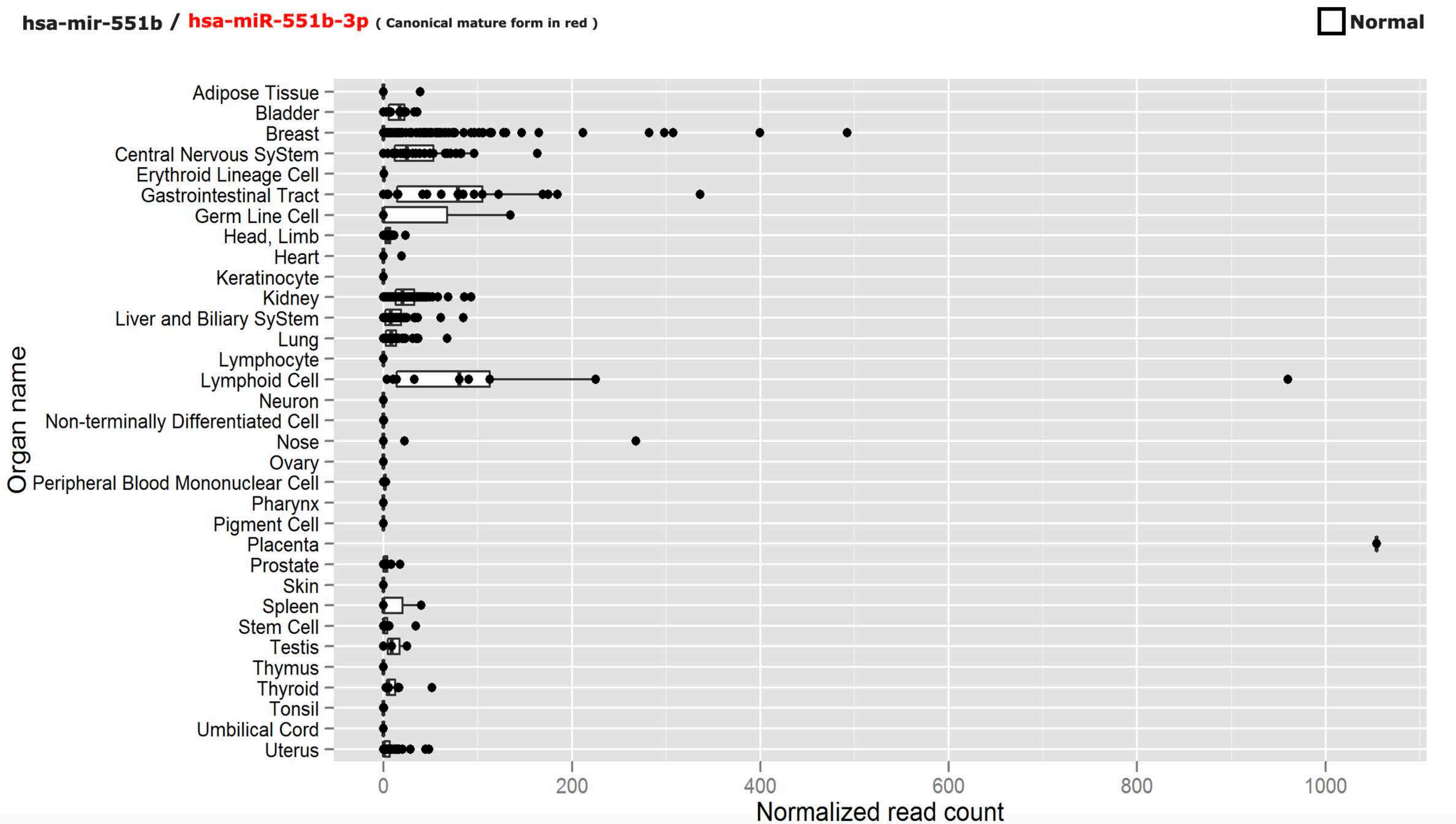
hsa-mir-551b / hsa-miR-551b-3p (Canonical mature form in red)
Normal
Organ name
Adipose Tissue
Bladder
Breast
Central Nervous SyStem
Erythroid Lineage Cell
Gastrointestinal Tract
Germ Line Cell
Head, Limb
Heart
Keratinocyte
Kidney
Liver and Biliary SyStem
Lung
Lymphocyte
Lymphoid Cell
Neuron
Non-terminally Differentiated Cell
Nose
Ovary
Peripheral Blood Mononuclear Cell
Pharynx
Pigment Cell
Placenta
Prostate
Skin
Spleen
Stem Cell
Testis
Thymus
Thyroid
Tonsil
Umbilical Cord
Uterus
0
200
400
600
800
1000
Normalized read count

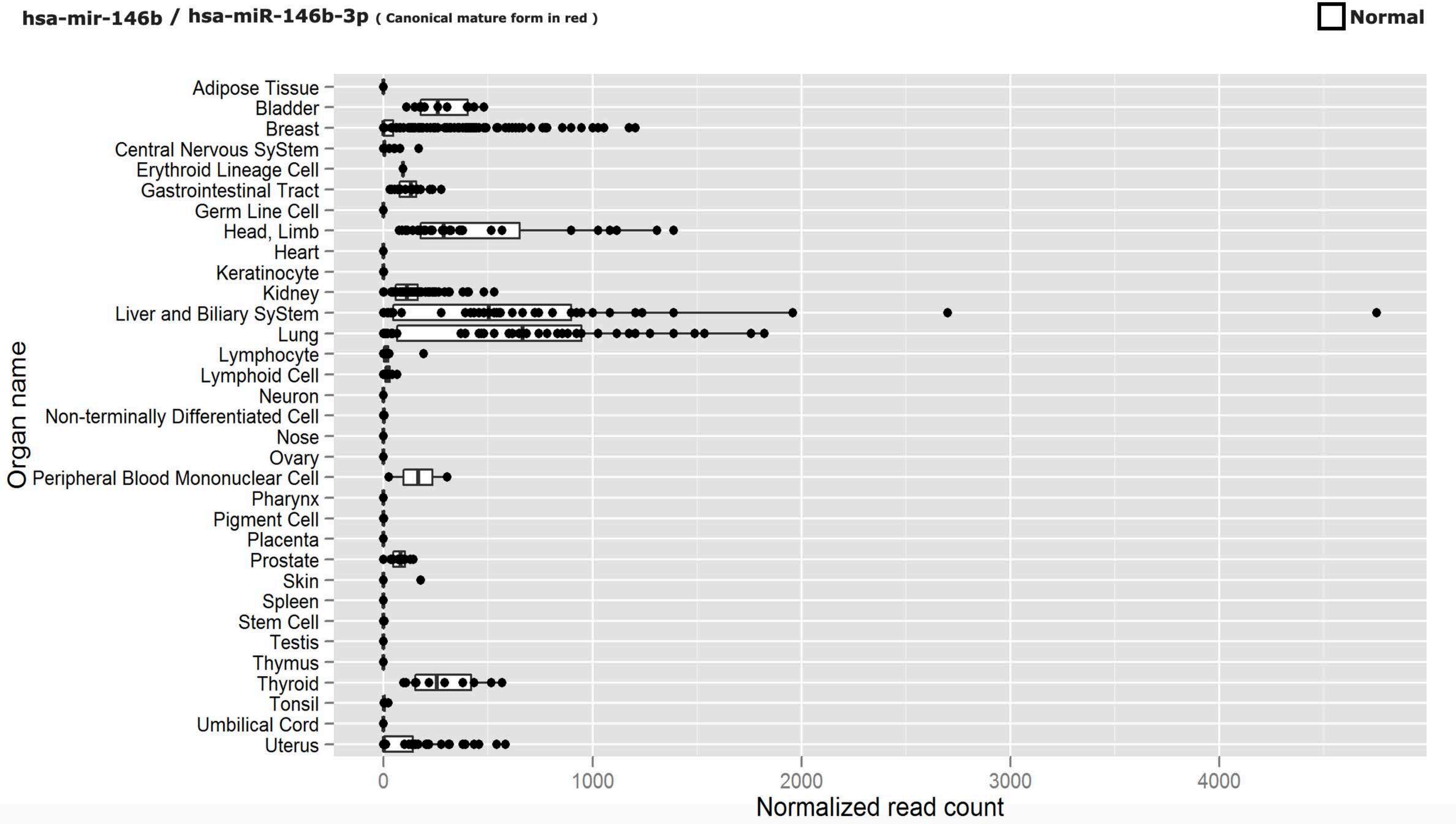
hsa-mir-146b / hsa-miR-146b-3p (Canonical mature form in red)
Normal
Adipose Tissue
Bladder
Breast
Central Nervous SyStem
Erythroid Lineage Cell
Gastrointestinal Tract
Germ Line Cell
Head, Limb
Heart
Keratinocyte
Kidney
Liver and Biliary SyStem
Lung
Lymphocyte
Lymphoid Cell
Neuron
Non-terminally Differentiated Cell
Nose
Ovary
Peripheral Blood Mononuclear Cell
Pharynx
Pigment Cell
Placenta
Prostate
Skin
Spleen
Stem Cell
Testis
Thymus
Thyroid
Tonsil
Umbilical Cord
Uterus
Organ name
0
1000
2000
3000
4000
Normalized read count

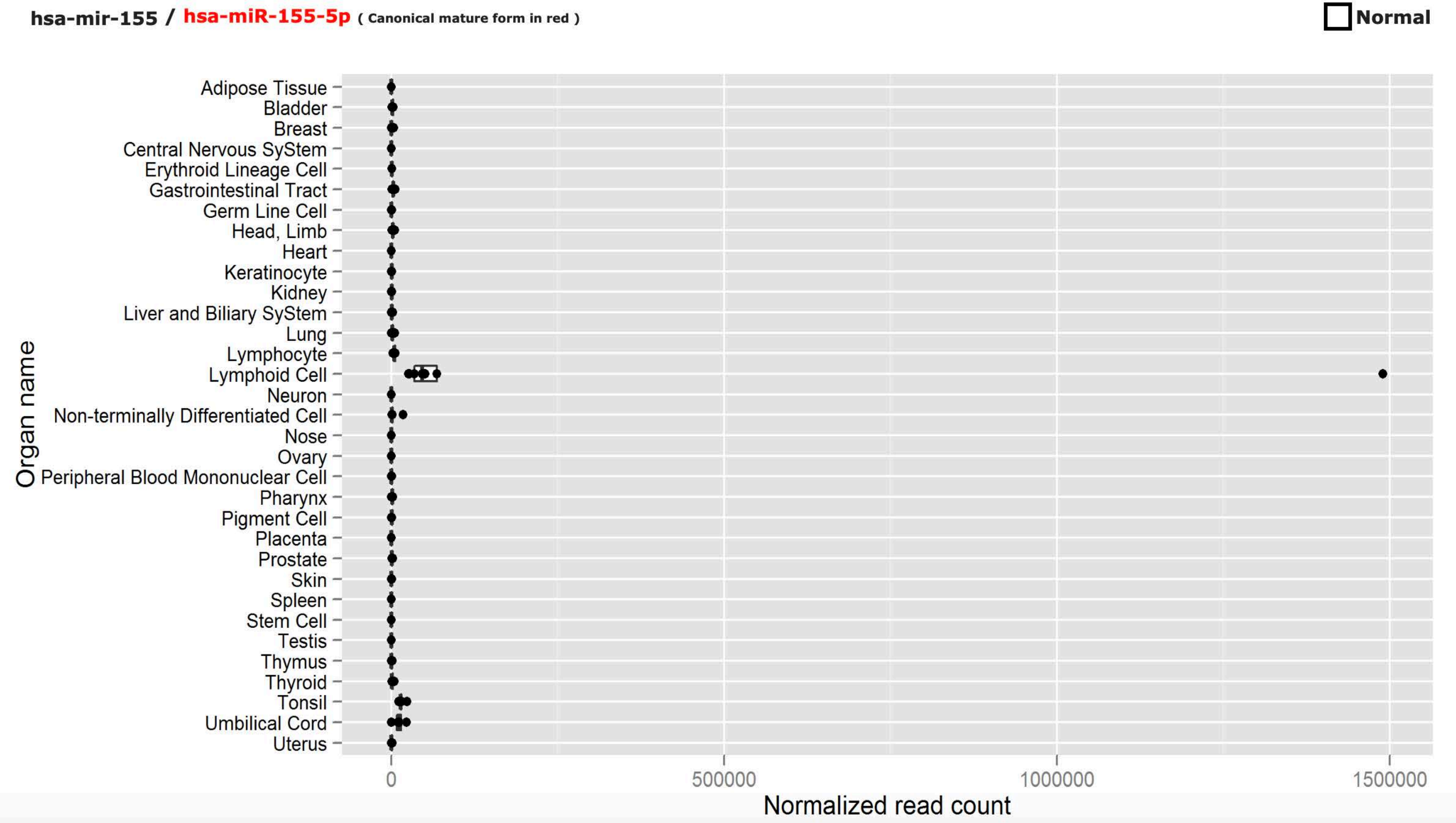
hsa-mir-155 / hsa-miR-155-5p (Canonical mature form in red)
Normal
Organ name
Adipose Tissue
Bladder
Breast
Central Nervous SyStem
Erythroid Lineage Cell
Gastrointestinal Tract
Germ Line Cell
Head, Limb
Heart
Keratinocyte
Kidney
Liver and Biliary SyStem
Lung
Lymphocyte
Lymphoid Cell
Neuron
Non-terminally Differentiated Cell
Nose
Ovary
Peripheral Blood Mononuclear Cell
Pharynx
Pigment Cell
Placenta
Prostate
Skin
Spleen
Stem Cell
Testis
Thymus
Thyroid
Tonsil
Umbilical Cord
Uterus
0
500000
1000000
1500000
Normalized read count

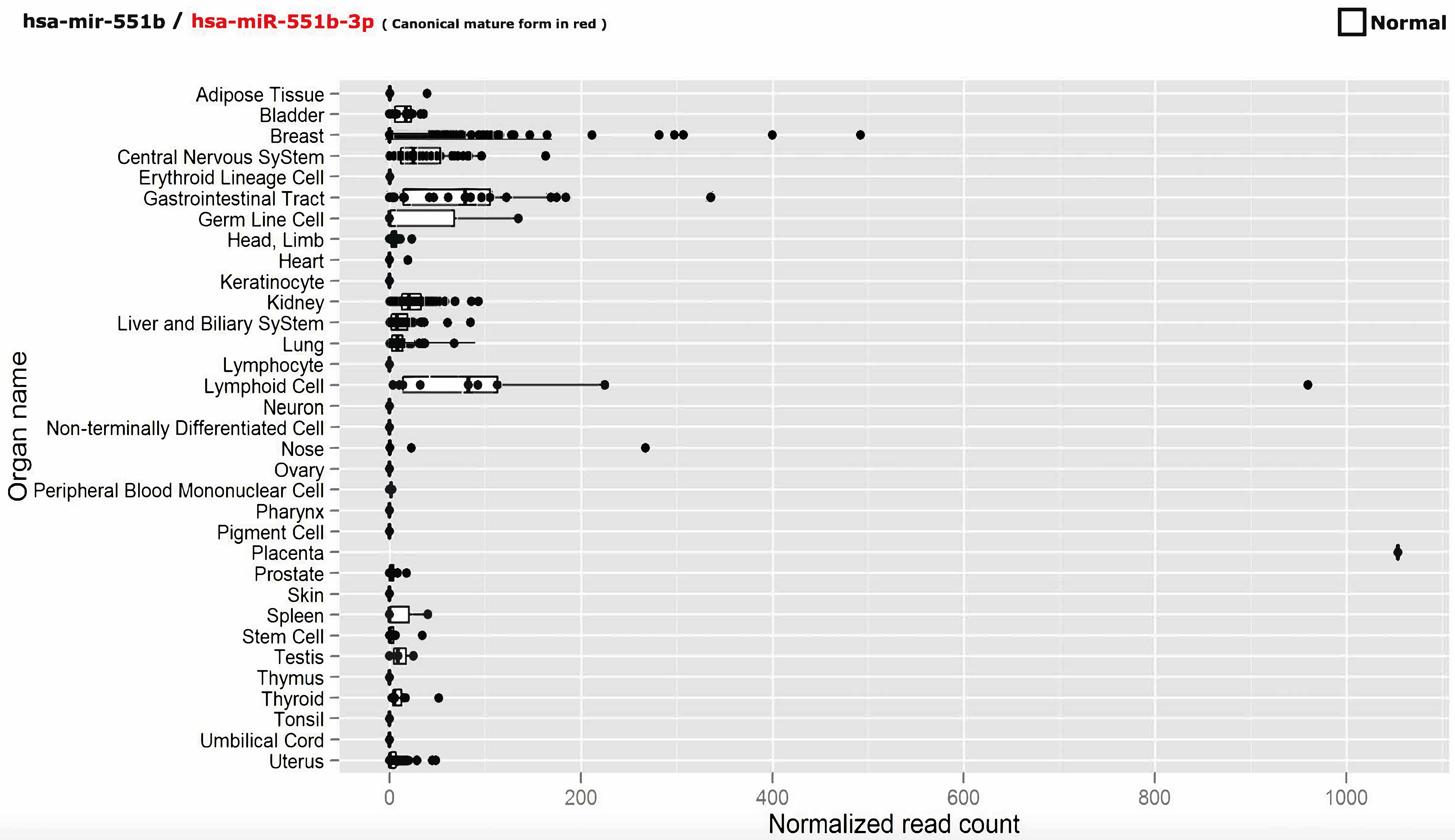
hsa-mir-551b / hsa-miR-551b-3p (Canonical mature form in red)
Normal
Organ name
Adipose Tissue
Bladder
Breast
Central Nervous SyStem
Erythroid Lineage Cell
Gastrointestinal Tract
Germ Line Cell
Head, Limb
Heart
Keratinocyte
Kidney
Liver and Biliary SyStem
Lung
Lymphocyte
Lymphoid Cell
Neuron
Non-terminally Differentiated Cell
Nose
Ovary
Peripheral Blood Mononuclear Cell
Pharynx
Pigment Cell
Placenta
Prostate
Skin
Spleen
Stem Cell
Testis
Thymus
Thyroid
Tonsil
Umbilical Cord
Uterus
0
200
400
600
800
1000
Normalized read count
