# Supplementary material for: Combination of hsa-miR-21-3p/ sTNF-RI/ IL12-p40 /CCL25 serves as a promising panel of diagnostic biomarkers for distinguishing malignant from benign nodules in papillary thyroid cancer
Source: Endocrine. 2026 Apr 27;91(1):146. doi: 10.1007/s12020-026-04612-9 (PMC13121238; doi:10.1007/s12020-026-04612-9)
Supplement: Supplementary file 5 — Supplementary Material 5 [file 12020_2026_4612_MOESM5_ESM.pdf]

Supplementary Table S1. The map of the Human Protein Array C7

POS	POS	NEG	NEG	Blank	Acrp30	AgRP	Angiopoietin-2	Amphiregulin	axl	bFGF	Beta-NGF	BTC	CCL28
POS	POS	NEG	NEG	Blank	Acrp30	AgRP	Angiopoietin-2	Amphiregulin	axl	bFGF	Beta-NGF	BTC	CCL28
CTACK	dtk	EGF-R	ENA-78	Fas/TNFRSF6	FGF-4	FGF-9	G-CSF	GITR ligand	GITR	GRO	GRO-alpha	HCC-4	HGF
CTACK	dtk	EGF-R	ENA-78	Fas/TNFRSF6	FGF-4	FGF-9	G-CSF	GITR ligand	GITR	GRO	GRO-alpha	HCC-4	HGF
ICAM-1	ICAM-3	IGF-BP-3	IGF-BP-6	IGF-I SR	IL-1 R4/ST2	IL-1 RI	IL11	IL12-p40	IL12-p70	IL17	IL-2 Ra	IL-6 R	IL8
ICAM-1	ICAM-3	IGF-BP-3	IGF-BP-6	IGF-I SR	IL-1 R4/ST2	IL-1 RI	IL11	IL12-p40	IL12-p70	IL17	IL-2 Ra	IL-6 R	IL8
I-TAC	Lymphotactin	MIF	MIP-1-alpha	MIP-1-beta	MIP-3-beta	MSP-a	NT-4	Osteoprotegerin	Oncostatin M	PIGF	sgp130	sTNF RII	sTNF-RI
I-TAC	Lymphotactin	MIF	MIP-1-alpha	MIP-1-beta	MIP-3-beta	MSP-a	NT-4	Osteoprotegerin	Oncostatin M	PIGF	sgp130	sTNF RII	sTNF-RI
TECK	TIMP-1	TIMP-2	TPO	TRAIL-R3	TRAIL-R4	uPAR	VEGF	VEGF-D	Blank	Blank	Blank	Blank	POS
TECK	TIMP-1	TIMP-2	TPO	TRAIL-R3	TRAIL-R4	uPAR	VEGF	VEGF-D	Blank	Blank	Blank	Blank	POS

POS Positive Control
NEG Negative Control

Combination of hsa-miR-21-3p/ sTNF-RI/ IL12-p40 /CCL25 serves as a promising panel of diagnostic biomarkers for distinguishing malignant from benign nodules in papillary thyroid cancer.

Abdulmelik Aytatli1,2, Abdulkadir Sahin3, Neslisah Barlak1,2, Betul Gundogdu4, Arzu Tatar3, Omer Faruk KARATAS1,2,*
