# Supplementary material for: Combination of hsa-miR-21-3p/ sTNF-RI/ IL12-p40 /CCL25 serves as a promising panel of diagnostic biomarkers for distinguishing malignant from benign nodules in papillary thyroid cancer
Source: Endocrine. 2026 Apr 27;91(1):146. doi: 10.1007/s12020-026-04612-9 (PMC13121238; doi:10.1007/s12020-026-04612-9)
Supplement: Supplementary file 7 — Supplementary Material 7 [file 12020_2026_4612_MOESM7_ESM.pdf]

Combination of hsa-miR-21-3p/ sTNF-RI/ IL12-p40 /CCL25 serves as a promising panel of diagnostic biomarkers for distinguishing malignant from benign nodules in papillary thyroid cancer.

Abdulmelik Aytatli1,2, Abdulkadir Sahin3, Neslisah Barlak1,2, Betul Gundogdu4, Arzu Tatar3, Omer Faruk KARATAS1,2,*

Supplementary Table S3. The Protein Array analysis was performed on all groups. The table includes the Log2FC and p-value.

	Malign			Benign		
	Vein/Artery(Fold Change)	Log2FC	p-Value	Vein/Artery(Fold Change)	Log2FC	p-Value
Acrp30	0.69007337	-0.5351783	0.00110577	0.714107117	-0.4857876	0.13818435
AgRP	0.494174119	-1.0169086	0.00030721	0.582078453	-0.7807145	0.09687697
Angiopoietin-2	0.733383877	-0.4473595	0.0277638	0.594252618	-0.7508517	0.04548693
Amphiregulin	0.872945903	-0.1960358	0.6516571	0.677772695	-0.5611266	0.10446225
axl	1.060394405	0.08460096	0.48358884	0.788415231	-0.3429724	0.07358946
bFGF	0.954259314	-0.0675467	0.73169011	0.631834685	-0.662381	0.0925202
Beta-NGF	0.688874697	-0.5376865	0.09944134	0.514292531	-0.9593389	0.00037016
BTC	0.675426598	-0.5661291	0.23572669	0.591265381	-0.7581223	0.00068868
CCL28	0.661204574	-0.5968314	0.16748414	0.489565006	-1.0304277	0.08581508
CTACK	1.001208427	0.00174234	0.98385536	1.140219371	0.18931142	0.01992924
dtk	1.63650129	0.71061474	0.00128073	1.356866713	0.44027901	0.00657736
EGF-R	0.794802016	-0.3313326	0.08518857	0.95796379	-0.061957	0.6290179
ENA-78	0.808811835	-0.306124	0.05365126	0.970550833	-0.0431243	0.00202837
Fas/TNFRSF6	0.842123748	-0.2478958	0.06310415	0.978405251	-0.0314959	0.75800398
FGF-4	0.753183856	-0.408926	0.06794244	1.065992106	0.09219675	0.55720284
FGF-9	0.801735563	-0.3188016	0.09245911	0.905761309	-0.1427972	0.26859583
G-CSF	0.74	-0.4344028	0.01670086	0.724304373	-0.465332	0.29778805
GITR ligand	0.808665306	-0.3063854	0.23841534	1.027406873	0.03900763	0.73395635
GITR	1.416170346	0.50199481	0.0537059	0.99661153	-0.0048968	0.95233591
GRO	0.715611806	-0.4827509	0.00922283	0.622650685	-0.6835051	0.00119033
GRO-alpha	0.957597736	-0.0625084	0.37235246	0.975021978	-0.0364934	0.58695753
HCC-4	1.287914318	0.36503662	0.02845016	1.057418466	0.08054643	0.44166637
HGF	1.009526557	0.01367886	0.89974717	0.474728004	-1.0748269	0.07857095
ICAM-1	0.620243486	-0.6890934	2.1223E-05	1.126045367	0.17126495	0.08309667
ICAM-3	0.798957952	-0.3238085	0.07023042	1.086491864	0.11967737	0.26797377
IGF-BP-3	0.928319214	-0.1073071	0.55631346	1.255962748	0.32879367	0.00819765
IGF-BP-6	1.025116331	0.03578764	0.00474363	1.04603714	0.06493408	0.46885642
IGF-I SR	1.089818071	0.12408732	0.39033019	1.192753365	0.25429576	0.08834285
IL-1 R4/ST2	0.679573343	-0.5572988	0.00521348	1.458306065	0.54429354	0.00241118
IL-1 RI	0.732645291	-0.4488132	0.00013563	1.018616255	0.02661065	0.72175351
IL11	0.443763859	-1.1721359	4.4021E-05	0.936433533	-0.0947515	0.68318313
IL12-p40	0.78768406	-0.344311	0.00017992	1.162805226	0.21760946	0.00987744
IL12-p70	0.421700371	-1.2457098	0.00232601	0.717736693	-0.4784734	0.10401365
IL17	0.418088874	-1.2581184	0.00045409	0.548108688	-0.8674661	0.00086208
IL-2 Ra	0.516834797	-0.9522249	0.00204201	0.681031842	-0.5542058	0.00515368
IL-6 R	0.716789658	-0.4803783	0.01570749	0.981971633	-0.0262467	0.83453837
IL8	0.675018424	-0.5670012	0.00025233	0.57937769	-0.787424	0.00060063
I-TAC	1.280011628	0.35615692	0.03936181	1.186604746	0.24683946	0.00582707
Lymphotactin	0.917670042	-0.1239526	0.28078678	1.039207966	0.0554844	0.75445146
MIF	1.198259405	0.26094026	0.05138808	1.126019243	0.17123148	0.00163485
MIP-1-alpha	1.195780838	0.257953	0.01427126	1.019627098	0.02804162	0.80213439
MIP-1-beta	1.810608583	0.8564747	0.00028703	1.468942607	0.55477803	0.0097682
MIP-3-beta	1.179702924	0.2384236	0.01278509	1.307115305	0.38638641	0.15117512
MSP-a	1.3689595	0.45307977	0.00102267	0.900840949	-0.1506557	0.0855267
NT-4	1.167890404	0.2239049	5.4334E-05	0.836254685	-0.2579857	0.00867443
Osteoprotegerin	0.975174616	-0.0362675	0.68606059	0.881663146	-0.1817005	0.11058348
Oncostatin M	1.381172913	0.46589395	0.03185924	0.974209252	-0.0376964	0.52868795
PIGF	0.776491976	-0.3649571	0.12753906	0.677475849	-0.5617586	0.00405149
sgp130	1.107031593	0.1466964	0.50527966	0.723400957	-0.4671326	0.00988353
sTNF RII	0.949818848	-0.0742757	0.76719287	0.667297285	-0.5835985	0.01686862
sTNF-RI	1.520020538	0.60409082	0.00012042	0.92015104	-0.1200574	0.02449421
TECK	0.706849379	-0.5005253	0.01955463	1.375758661	0.46022741	4.6294E-05
TIMP-1	0.928684053	-0.1067402	0.06935742	1.734709948	0.79469446	0.00056852
TIMP-2	0.828727564	-0.2710302	0.00048809	1.060760115	0.08509844	0.20361162
TPO	1.022121777	0.03156709	0.8397402	0.982723996	-0.0251418	0.85168803
TRAIL-R3	0.860282539	-0.2171175	0.18205884	1.053003934	0.07451083	0.61067075
TRAIL-R4	1.243690472	0.31462747	0.06092331	1.035612989	0.05048496	0.42272053
uPAR	0.602893581	-0.7300247	0.00137665	1.095743627	0.13191029	0.11512221
VEGF	0.749523	-0.4159553	0.00887684	1.61077619	0.68775605	0.0038659
VEGF-D	0.663803336	-0.5911722	0.0047137	1.324057683	0.40496598	0.10360455

	Peripheral Blood Serum			Peripheral Blood Serum		
	Malign/Benign(Fold Change)	Log2FC	p-Value	Post Op./ Pre Op. (Fold Change)	Log2FC	p-Value
Acrp30	0.634308779	-0.6567428	0.05556698	0.834738943	-0.260603	0.33078336
AgRP	0.443601128	-1.1726651	0.05227547	0.573006942	-0.8033755	0.00239382
Angiopoietin-2	0.601260412	-0.7339381	0.00821029	0.737019248	-0.4402258	0.04458127
Amphiregulin	0.991648739	-0.0120989	0.92569437	1.044198968	0.06239664	0.45361136
axl	0.8773914	-0.1887075	0.27071833	0.743656221	-0.4272923	0.06498945
bFGF	1.024315011	0.03465946	0.73417039	0.994808636	-0.0075091	0.86472493
Beta-NGF	0.492496037	-1.021816	0.00095567	0.676017159	-0.5648682	0.00063087
BTC	0.253535169	-1.9797422	0.00013109	0.756304101	-0.4029617	0.011692
CCL28	0.189947682	-2.396326	1.3716E-06	0.345379367	-1.5337462	0.00010562
CTACK	1.014568586	0.0208664	0.58563001	0.830017329	-0.2687866	0.00335309
dtk	0.693686093	-0.5276451	0.00019754	0.90510845	-0.1438374	0.00015659
EGF-R	0.370138835	-1.4338616	8.8551E-10	0.765490286	-0.385544	0.0695953
ENA-78	0.358478111	-1.4800431	2.4372E-05	0.509599388	-0.9725645	1.2081E-06
Fas/TNFRSF6	0.50927832	-0.9734738	0.00027839	0.604166739	-0.7269813	0.00753432
FGF-4	0.505579882	-0.983989	0.00114212	0.912072696	-0.1327793	0.00330024
FGF-9	0.943321832	-0.084178	0.13936454	0.675500909	-0.5659704	0.00028258
G-CSF	1.108108108	0.14809864	0.62330071	0.438850204	-1.1881995	0.03213827
GITR ligand	0.856114428	-0.2241245	0.18121635	0.83175658	-0.2657667	0.0682556
GITR	1.123711803	0.16827208	0.11868715	0.63943436	-0.6451318	1.9758E-05
GRO	0.789723961	-0.3405796	0.00147735	0.404905725	-1.3043421	4.549E-05
GRO-alpha	0.960687482	-0.0578609	0.02853442	0.628044657	-0.6710609	1.8882E-06
HCC-4	1.324348654	0.40528298	0.0111646	0.749445501	-0.4161045	0.008123
HGF	1.237924329	0.30792313	0.10233141	0.612971372	-0.7061084	1.6266E-05
ICAM-1	0.45224344	-1.1448285	0.00017313	0.770767983	-0.3756315	0.00055376
ICAM-3	0.651770813	-0.6175633	0.0006272	0.575967865	-0.7959398	0.00218212
IGF-BP-3	0.615721416	-0.6996503	0.000654	0.937000116	-0.0938789	0.72324834
IGF-BP-6	0.440781132	-1.1818656	0.00698022	0.92919081	-0.1059532	0.61673598
IGF-I SR	0.760801994	-0.3944071	0.00617777	0.888243024	-0.1709736	0.28980207
IL-1 R4/ST2	0.530160275	-0.9154995	0.00014461	0.853552592	-0.228448	0.2231909
IL-1 RI	0.848814388	-0.236479	0.00366696	0.751006735	-0.4131022	0.00940239
IL11	0.305067061	-1.7128017	0.01023966	0.724683905	-0.4645762	0.09209693
IL12-p40	0.671523824	-0.5744895	8.8281E-05	1.026470047	0.03769153	0.41561559
IL12-p70	0.230456417	-2.1174342	0.0963942	0.74237311	-0.4297836	0.16283313
IL17	0.73528572	-0.4436231	0.03265585	0.578698499	-0.7891162	0.03867947
IL-2 Ra	1.05818909	0.08159745	0.13410631	0.699193947	-0.5162354	0.00047523
IL-6 R	1.247218374	0.31871409	0.01569754	1.007030174	0.01010691	0.94308826
IL8	1.302052134	0.38078722	0.1409337	0.953654978	-0.0684607	0.33179374
I-TAC	0.628134789	-0.6708539	0.02258539	0.956980339	-0.0634388	0.41722441
Lymphotactin	0.61822333	-0.6938	0.00669895	0.994590632	-0.0078253	0.93410152
MIF	0.619408507	-0.6910369	0.00507435	0.914594324	-0.1287961	0.15526907
MIP-1-alpha	0.88172268	-0.1816031	0.34772735	1.023765578	0.0338854	0.75714934
MIP-1-beta	1.115036266	0.15709063	0.48372751	1.460143352	0.54611001	0.01590139
MIP-3-beta	0.602509975	-0.730943	0.16098073	1.306652668	0.3858757	0.05085575
MSP-a	0.957920934	-0.0620215	0.76645839	1.012093843	0.01734307	0.87507781
NT-4	0.840020831	-0.251503	0.1891832	0.835095861	-0.2599863	0.11927326
Osteoprotegerin	0.825016118	-0.2775058	0.15703035	0.710365412	-0.4933668	0.0716335
Oncostatin M	1.024866926	0.03543659	0.79906791	0.748046152	-0.4188008	0.01547419
PIGF	0.657333403	-0.6053028	0.01682998	0.462819495	-1.1114785	0.00015355
sgp130	0.900055383	-0.1519143	0.29851113	0.664880405	-0.5888332	0.00039061
sTNF RII	0.724556877	-0.4648292	0.0440957	0.53881051	-0.8921501	0.02977834
sTNF-RI	1.716620817	0.7795714	0.00458505	0.594730065	-0.7496931	0.00180178
TECK	0.611536155	-0.7094903	0.0047367	1.309573388	0.38909691	0.02524011
TIMP-1	1.21352249	0.27920085	0.01422097	0.742901696	-0.4287568	0.10259722
TIMP-2	1.115161761	0.157253	0.14564997	0.714324934	-0.4853476	0.05868823
TPO	0.987156271	-0.0186496	0.90388633	0.752256187	-0.410704	0.02721301
TRAIL-R3	0.520389666	-0.9423358	0.00254628	0.898769652	-0.1539767	0.05779497
TRAIL-R4	1.230856522	0.2996626	0.00763694	0.644796358	-0.6330845	0.04544097
uPAR	0.841729319	-0.2485717	0.21370866	0.390379652	-1.3570502	0.00612875
VEGF	1.121528445	0.16546621	0.00341681	0.893072522	-0.1631508	0.36748401
VEGF-D	0.847186807	-0.239248	0.0467071	1.771096796	0.82464306	0.07795735
