# Supplementary material for: Combination of hsa-miR-21-3p/ sTNF-RI/ IL12-p40 /CCL25 serves as a promising panel of diagnostic biomarkers for distinguishing malignant from benign nodules in papillary thyroid cancer
Source: Endocrine. 2026 Apr 27;91(1):146. doi: 10.1007/s12020-026-04612-9 (PMC13121238; doi:10.1007/s12020-026-04612-9)
Supplement: Supplementary file 8 — Supplementary Material 8 [file 12020_2026_4612_MOESM8_ESM.docx]

| **Count** | **Sample** | **IL12-p40** | **sTNF-RI** | **CCL25** | **hsa-miR-21-3p** |
| --- | --- | --- | --- | --- | --- |
| 1 | Benign | 144.598039 | 17.5006859 | 159.747126 | 10.4468783 |
| 2 | Benign | 188.127451 | 24.4348422 | 212.16092 | 15.03236399 |
| 3 | Benign | 173.568627 | 22.0068587 | 227.505747 | 5.351710219 |
| 4 | Benign | 175.333333 | 24.8875171 | 231.988506 | 7.086140153 |
| 5 | Benign | 181.95098 | 12.6749275 | 308.712644 | 3.82378127 |
| 6 | Benign | 154.745098 | 23.5089163 | 196.384 | 1 |
| 7 | Benign | 147.245098 | 21.5336077 | 186.816092 | 9.031715238 |
| 8 | Benign | 176.964444 | 20.9986283 | 178.022989 | 5.278031643 |
| 9 | Benign | 144.097778 | 13.7488406 | 218.88 | 1.244011653 |
| 10 | Benign | 685.888889 | 13.9966667 | 206.72 | 1.647182035 |
| 11 | Benign | 159.564444 | 12.8814493 | 216.448 | 7.862564788 |
| 12 | Benign | 153.12 | 11.8110333 | 193.952 | 4.707626949 |
| 1 | Malign | 165.77451 | 23.5089163 | 191.643678 | 10.55606329 |
| 2 | Malign | 141.068627 | 20.1344307 | 213.367816 | 4.658934346 |
| 3 | Malign | 172.245098 | 24.6406036 | 211.298851 | 35.38338091 |
| 4 | Malign | 204.303922 | 22.8093278 | 242.16092 | 9.952093226 |
| 5 | Malign | 170.186275 | 24.1262003 | 214.91954 | 14.27085631 |
| 6 | Malign | 206.509804 | 24.5994513 | 188.885057 | 44.63179732 |
| 7 | Malign | 163.568627 | 21.9657064 | 190.264368 | 11.71268557 |
| 8 | Malign | 182.098039 | 19.8257888 | 221.988506 | 11.19668692 |
| 9 | Malign | 162.539216 | 20.8340192 | 212.505747 | 219.0323266 |
| 10 | Malign | 133.715686 | 27.0274348 | 170.781609 | 11.39240156 |
| 11 | Malign | 142.686275 | 21.7805213 | 203.195402 | 18.25221945 |
| 12 | Malign | 154.745098 | 19.7229081 | 316.471264 | 22.54913208 |
| 13 | Malign | 119.598039 | 19.7846365 | 230.091954 | 3.434261746 |
| 14 | Malign | 134.892157 | 26.3484225 | 217.16092 | 9.747282109 |
| 15 | Malign | 243.862745 | 23.2208505 | 296.643678 | 5.098242509 |
| 16 | Malign | 125.921569 | 24.9903978 | 242.678161 | 6.750526369 |
| 17 | Malign | 135.921569 | 31.739369 | 220.609195 | 6.041890342 |
| 18 | Malign | 242.245098 | 27.1920439 | 155.091954 | 18.06343048 |
| 19 | Malign | 146.95098 | 39.4348422 | 214.574713 | 213.7825074 |
| 20 | Malign | 233.568627 | 22.4183813 | 174.229885 | 18.37917368 |
| 21 | Malign | 147.245098 | 24.4759945 | 329.057471 | 4.547267893 |
| 22 | Malign | 161.215686 | 23.6941015 | 173.022989 | 11.08087574 |
| 23 | Malign | 122.539216 | 18.8792867 | 179.91954 | 222.8609442 |
| 24 | Malign | 151.509804 | 36.3484225 | 158.54023 | 4.141059695 |
| 25 | Malign | 124.892157 | 21.1838134 | 193.195402 | 5.010657754 |
| 26 | Malign | 135.039216 | 19.3525377 | 180.091954 | 176.0693527 |
| 27 | Malign | 145.333333 | 20.2578875 | 221.298851 | 118.1929358 |
| 28 | Malign | 134.156863 | 21.2249657 | 178.54023 | 148.0560875 |
| 29 | Malign | 141.362745 | 20.5871056 |  | 3.458148925 |
| 30 | Malign | 152.980392 | 22.6035665 |  |  |
| 31 | Malign | 271.215686 | 18.303155 |  |  |
| 32 | Malign | 152.392157 |  |  |  |
| 33 | Malign | 180.627451 |  |  |  |
